# Insights into the evolutionary history of tubercle bacilli as disclosed by genetic rearrangements within a PE_PGRS duplicated gene pair

**DOI:** 10.1186/1471-2148-6-107

**Published:** 2006-12-12

**Authors:** Anis Karboul, Nicolaas C Gey van Pittius, Amine Namouchi, Véronique Vincent, Christophe Sola, Nalin Rastogi, Philip Suffys, Michel Fabre, Angel Cataldi, Richard C Huard, Natalia Kurepina, Barry Kreiswirth, John L Ho, M Cristina Gutierrez, Helmi Mardassi

**Affiliations:** 1 Unit of Typing and Genetics of Mycobacteria, Institut Pasteur de Tunis, 13 Place Pasteur, 1002 Tunis-Belvédère, Tunis, Tunisie; 2 DST/NRF Centre of Excellence in Biomedical Tuberculosis Research, MRC Centre for Molecular and Cellular Biology, Department of Biomedical Sciences, Faculty of Health Sciences, Stellenbosch University, South Africa; 3 Laboratoire de Référence des Mycobactéries, Institut Pasteur, Paris, France; 4 Unité de la Tuberculose et des Mycobactéries, Institut Pasteur de Guadeloupe, Guadeloupe; 5 Laboratory of Molecular Biology and Diagnosis of Infectious Diseases, Oswaldo Cruz Institute, Brazil; 6 Laboratoire de Biologie Clinique, HIA Percy, Clamart, France; 7 Instituto de Biotecnologia, INTA, Castelar, Buenos Aires, Argentina; 8 Clinical Microbiology Service and the Department of Pathology, Columbia University Medical Center, New York-Presbyterian Hospital, New York, NY, USA; 9 Public Health Research Institute (PHRI), Newark, New Jersey, USA; 10 Division of International Medicine and Infectious Diseases, Weill Medical College of Cornell University, New York, NY, USA; 11 Unit of Mycobacterial genetics, Institut Pasteur, Paris, France

## Abstract

**Background:**

The highly homologous PE_PGRS (Proline-glutamic acid_polymorphic GC-rich repetitive sequence) genes are members of the PE multigene family which is found only in mycobacteria. PE genes are particularly abundant within the genomes of pathogenic mycobacteria where they seem to have expanded as a result of gene duplication events. PE_PGRS genes are characterized by their high GC content and extensive repetitive sequences, making them prone to recombination events and genetic variability.

**Results:**

Comparative sequence analysis of *Mycobacterium tuberculosis *genes PE_PGRS17 (Rv0978c) and PE_PGRS18 (Rv0980c) revealed a striking genetic variation associated with this typical tandem duplicate. In comparison to the *M. tuberculosis *reference strain H37Rv, the variation (named the 12/40 polymorphism) consists of an in-frame 12-bp insertion invariably accompanied by a set of 40 single nucleotide polymorphisms (SNPs) that occurs either in PE_PGRS17 or in both genes. Sequence analysis of the paralogous genes in a representative set of worldwide distributed tubercle bacilli isolates revealed data which supported previously proposed evolutionary scenarios for the *M. tuberculosis *complex (MTBC) and confirmed the very ancient origin of "*M. canettii*" and other smooth tubercle bacilli. Strikingly, the identified polymorphism appears to be coincident with the emergence of the post-bottleneck successful clone from which the MTBC expanded. Furthermore, the findings provide direct and clear evidence for the natural occurrence of gene conversion in mycobacteria, which appears to be restricted to modern *M. tuberculosis *strains.

**Conclusion:**

This study provides a new perspective to explore the molecular events that accompanied the evolution, clonal expansion, and recent diversification of tubercle bacilli.

## Background

*Mycobacterium tuberculosis *complex strains (MTBC) are the causative agents of tuberculosis (TB), a disease that has a considerable detrimental impact on human and animal health worldwide [1]. This group of slow growing pathogens includes the classical *M. tuberculosis*, *M. bovis*, *M. africanum*, *M. microti*, as well as the newly recognized MTBC members, *M. pinnipedii *and *M. caprae *species. *M. tuberculosis *remains one of the most successful and adaptable pathogens known to mankind despite the availability of a vaccine and effective antimicrobial agents. This adaptability certainly reflects a very ancient and prolific evolutionary history.

With the availability of complete mycobacterial genome sequences, whole-genome comparative sequence analyses were possible and resulted in the identification of sequence polymorphisms, that greatly inform our understanding of the evolutionary process of the MTBC [2-14]. It is now assumed that *M. tuberculosis *(the major etiological agent of human TB) and *M. bovis *(having a wide host range) both arose from a common ancestor [15,16]. It has also become apparent that the *M. africanum*-*M. microti *lineage represents a phylogenetic bridge between *M. tuberculosis *and *M. bovis*, whereas "*M. canettii*", a rare phenotypically unusual tubercle bacillus, appears to be closest to the common progenitor of the MTBC [17,18]. Recent studies confirmed that "*M. canettii*" and other smooth tubercle bacilli are representatives of pre-bottleneck lineages and that the progenitor species (the so-called *M. prototuberculosis*), from which the MTBC emerged, might have coexisted with early hominids [19,20].

Completion of the genome sequence of *M. tuberculosis *strain H37Rv [2], revealed that a major source of genetic variation in this species could be associated with two large gene families encoding acidic, asparagine- or glycine-rich proteins referred to as PE (n = 99) and PPE (n = 68). These multigene families represent approximately 10% of the coding capacity of the genome and are characterized by their high GC content and extensive repetitive structure. Both families have been divided into subgroups, of which the PE_PGRS subfamily (n = 61) of the PE family is particularly polymorphic and found to be enriched in essential genes [21]. Although the function of the members of this gene subfamily is currently unknown, the PE_PGRS genes are strongly suspected to be associated with antigenic and genetic variability as well as virulence [22-31]. It is thought that members of the PE/PPE multigene families might frequently undergo genetic remodelling by gene duplication, recombination, and/or strand slippage mechanisms because of the presence of a large number of repeat sequences within these genes [2]. In the current study, we focused on a prominent polymorphism motif that occurs within two adjacent PE_PGRS genes, and provide evidence for its association with both early and recent evolutionary events leading to a new PE_PGRS-based perspective to dissect the evolution of tubercle bacilli.

## Results

### Comparative sequence analysis of contiguous PE genes

**Figure 1:**
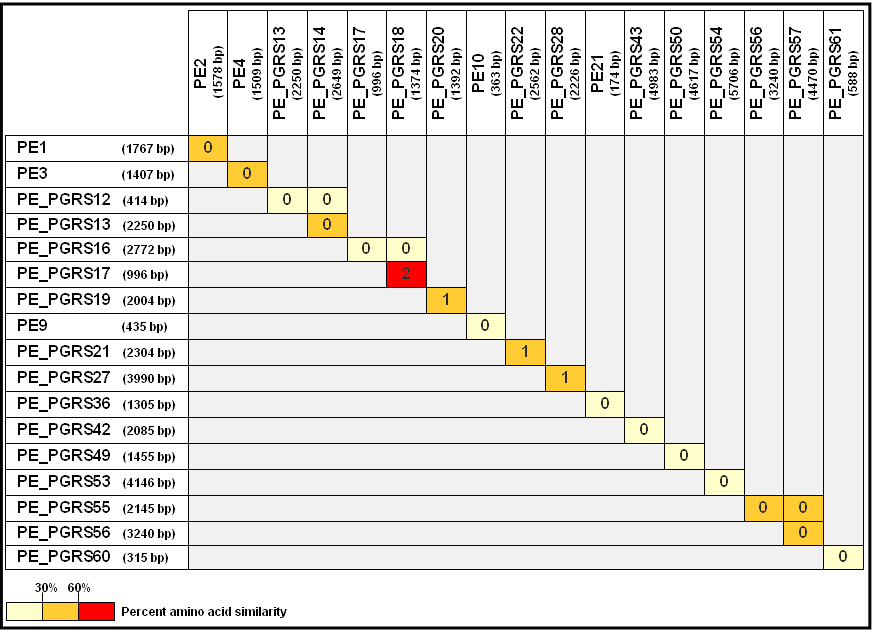
Pairwise amino acid similarity of contiguous PE genes of the *M. tuberculosis *strain H37Rv. The percent amino acid similarity values were calculated using the BioEdit program [52].

Since the large number of homologous *M. tuberculosis *PE genes seem to have arisen by multiple gene duplication events, it is very difficult to determine duplication history. We thus focussed our comparative sequence analysis on PE genes that are situated adjacent to each other in the *M. tuberculosis *H37Rv genome, as these tandem duplications (those where the two copies of the duplicated region are immediately adjacent to one another in the same orientation), could signify real duplicates. Fourteen neighbouring PE genes (11 apparent duplicates and 3 apparent triplicates) were identified throughout the H37Rv genome. About half of the contiguous PE sequences display more than 30% overall amino acid sequence similarity with their neighbouring PE gene member (Figure 1). As expected, a higher degree of similarity was associated with the conserved N-terminal PE region of the gene (Additional file 1), as previously reported [2]. Genes with low amino acid similarity were excluded from further analyses due to an uncertainty concerning their origin.

**Figure 2:**
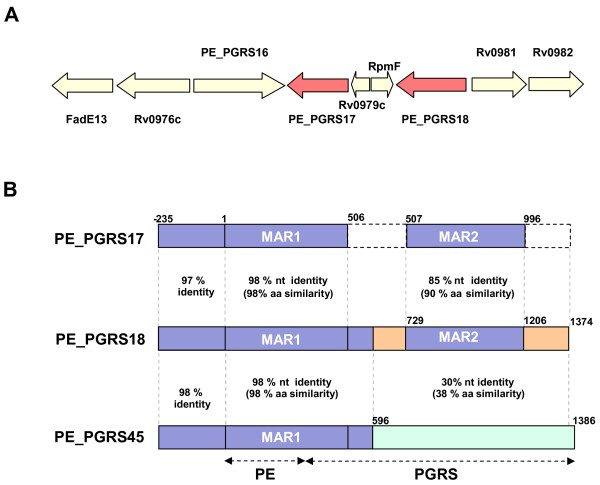
Genomic context and homology of the PE_PGRS17 and PE_PGRS18 genes. Genetic map showing the genomic context of the PE_PGRS17 (Rv0978c) and PE_PGRS18 (Rv0980c) genes within the genome of *M. tuberculosis *H37Rv. (B) Schematic representation of the homology shared between the PE_PGRS17 and PE_PGRS18 nucleotide sequences. Note the sequence relatedness with the PE_PGRS 45 (Rv2615c). MAR: major alignable coding region.

Further scrutiny of the nucleotide and amino acid alignments, showed that the PE_PGRS17 (Rv0978c) and PE_PGRS18 (Rv0980c) genes (Figure 2A) shared the highest identity, indicating that they may represent a true duplication event. Within these two genes, we could identify 2 major alignable coding regions of 168 (MAR1) and 162 (MAR2) amino acids, with a similarity of 98% and 90%, respectively (Figure 2B). These genes thus fulfilled the criteria used previously to define the bacterial paranome [32] and should be regarded as a typical tandem duplicate in spite of being separated by two overlapping, non-associated, non-PE genes (Rv0979c – encoding a hypothetical protein of unknown function and Rv0979A – encoding a probable 50S ribosomal protein L32 rpmF). The first alignable coding region starts at the first codon and extends beyond the PE region, while the second maps to the highly repetitive and GC rich (and usually very variable) PGRS C-terminal extension. PE_PGRS18 is larger than PE_PGRS17 essentially because of the presence of two extra in-frame nucleotide stretches, the first (222 nt) is located between the two major alignable coding regions and, the second (168 nt) represents an extra C-terminal extension (Figure 2B). Further inspection of the upstream non coding region of both genes revealed a nearly perfect homology starting at the nucleotide position -235. Furthermore, a BLASTN search performed against the whole genome of *M. tuberculosis *H37Rv identified another PE_PGRS gene, PE_PGRS45 (Rv2615c), sharing 98% identity through nucleotides -235 to 506 with PE_PGRS17 and 98% identity through -235 to 595 with PE_PGRS18. PE_PGRS45, which is located approximately 2.6 MB distal in the genome, is thus clearly a paralog of PE_PGRS17 and PE_PGRS18, and is either the progenitor of these two genes or was duplicated from one of them. In a recent study, the 3 PE_PGRS orthologs were shown to share 5 nts at polymorphic positions lying upstream of their coding sequences [33], thus confirming their evolutionary link. Sequence analyses of the genome sequence of the phylogenetically closest non-tuberculous mycobacterial species, *M. marinum*, showed that its genome was devoid of both the PE_PGRS17 and PE_PGRS18 orthologs (See Additional file 3), as well as lacking the orthologue for PE_PGRS45 (data not shown). As these genes were also found to be absent from other species of mycobacteria (e.g. *M. leprae*, *M. ulcerans*, and *M. avium paratuberculosis*-see Additional file 3), the duplication of these three PE_PGRS genes thus seems to have taken place only after the divergence of the tubercle bacilli.

#### Identification of a prominent genetic variation in PE_PGRS17 and PE_PGRS18 coding sequences

**Figure 3:**
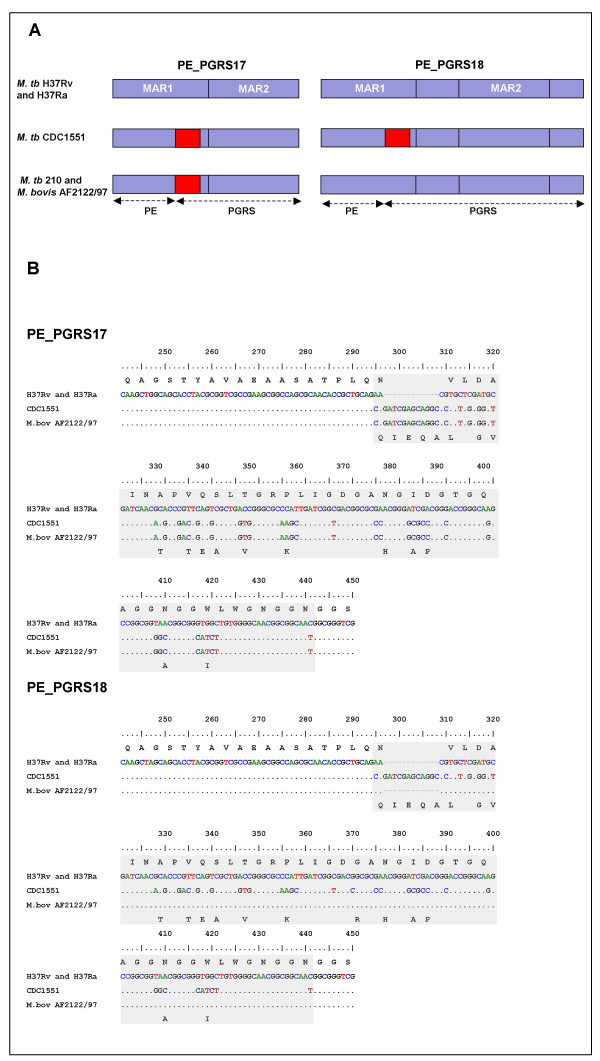
Distribution of the 12/40 polymorphism. (A). Schematic representation of PE_PGRS17 and PE_PGRS18 showing the distribution of the 12/40 polymorphism (red block) between paralogous and orthologous sequences. (B). Multiple sequence alignment of the 12/40 polymorphism region (highlighted with grey) in PE_PGRS17 and PE_PGRS18 of the three sequenced mycobacterial genomes of *M. tuberculosis *H37Rv (H37Rv), *M. tuberculosis *CDC1551 (CDC1551) and *M. bovis *AF2122/97 (M. bov AF2122/97). The corresponding sequences in *M. tuberculosis *strain H37Ra were determined by sequencing and proved to be identical to H37Rv. MAR: major alignable coding region.

Alignment of PE_PGRS17 and PE_PGRS18 with their corresponding published sequences (*M. tuberculosis *reference strains CDC1551 and 210, and *M. bovis *AF2122/97) revealed a prominent genetic variation associated with the major alignable coding region 1 that was either restricted to PE_PGRS17 (*M. bovis *and *M. tuberculosis *strain 210) or shared by both PE_PGRS17 and PE_PGRS18 genes (*M. tuberculosis *CDC1551) (Figure 3A). In comparison to the genome of the *M. tuberculosis *reference strain H37Rv, this genetic variation (termed the 12/40 polymorphism) consisted of a 12-nucleotide in-frame insertion accompanied by 40 SNPs dispersed along a DNA stretch of 135 nucleotides (Figure 3B), encompassing the junction between the PE and PGRS regions of the gene sequence (Figure 3A). This variation results in a 4-amino acid insertion and 15 amino acid changes. In contrast, although PE_PGRS45 shared the MAR1 region (which contains the 12/40 polymorphism) with PE_PGRS17 and PE_PGRS18, no polymorphism occurred in the orthologs of this gene in the abovementioned sequenced strains (H37Rv, CDC1551, 210, as well as *M. bovis *AF2122/97).

### Distribution of the 12/40 polymorphism throughout a worldwide collection of tubercle bacilli

**Figure 4:**
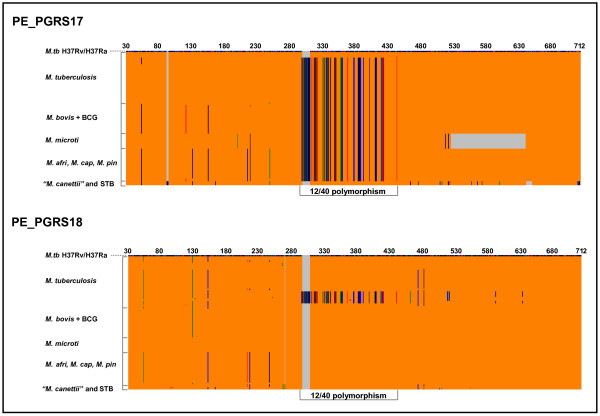
A plot graph showing the genetic variability within PE_PGRS17 and PE_PGRS18. The plot shows the distribution of the 12/40 polymorphism and the other SNPs among 101 worldwide distributed tubercle bacilli isolates (98 tubercle bacilli sequenced in this study along with the sequences from *M. tuberculosis *reference strains H37Rv, CDC1551, and *M. bovis *reference strain AF2122/97). SNPs (relative to the *M. tuberculosis *reference strain H37Rv) are shown in colours other than orange, whereas grey background indicates the presence of a deletion. *M. tb *– *M. tuberculosis*, *M. afri *– *M. africanum*, *M. cap *– *M. caprae*, *M. pin *– *M. pinnipedii*, STB-Smooth tubercle bacilli.

Based on the above observations, we decided to extend our analysis to an additional group of 98 genetically diverse and worldwide distributed tubercle bacilli. This group consisted of *M. tuberculosis *H37Ra, 38 other *M. tuberculosis *strains *sensu stricto*, 19 *M. bovis *strains, 1 *M. bovis *bacille Calmette-Guérin (BCG) strain (Danish strain, Statens Serum Institute), 22 *M. africanum *strains, 10 *M. microti *strains, 1 dassie bacillus, 1 *M. caprae *strain, 1 *M. pinnipedii *strain, 2 "*M. canettii" *strains, and 2 smooth tubercle bacilli strains (See Additional file 4, highlighted in yellow). For this purpose, the DNA sequence encompassing nucleotides 31 to 712 (numbering according to the *M. tuberculosis *H37Rv sequence) for both PE_PGRS17 and PE_GRS18 was determined in all these tubercle bacilli strains. The multiple sequence alignment (Figure 4) showed the absence, in both genes, of the 12/40 polymorphism in *M. tuberculosis *H37Ra, the two "*M. canettii*" strains and in one smooth tubercle bacillus isolate. For the second smooth tubercle bacillus isolate, we have been able to amplify only PE_PGRS18 which was found to be devoid of such a polymorphism. Strikingly, all strains of the *M. africanum*-*M. microti*-*M. caprae*-*M. pinnipedii*-*M. bovis *lineage (including *M. bovis *BCG), as well as the ancestral (TbD1+) and PGG1 strains of *M. tuberculosis*, harbor the 12/40 polymorphism in only their PE_PGRS17 gene sequence and not in PE_PGRS18. This finding was supported by analysis of the sequenced whole genome of *M. microti *strain OV254 (data not shown). In contrast, the pool of *M. tuberculosis *bacilli belonging to PGG2 and PGG3 appears as a mix of 2 subpopulations; one showing the variation uniquely in PE_PGRS17, while the second displays the genetic change in both paralogs. Interestingly, similar to the PGG2 epidemic *M. tuberculosis *reference strain CDC1551, a PGG2 outbreak-associated Tunisian *M. tuberculosis *Haarlem3 strain [34] displayed the 12/40 polymorphism in both PE_PGRS paralogs.

### Development of a reverse hybridization-based assay for the detection of the 12/40 polymorphism and confirmation of its non-random distribution throughout the evolutionary history of the MTBC

**Figure 5:**
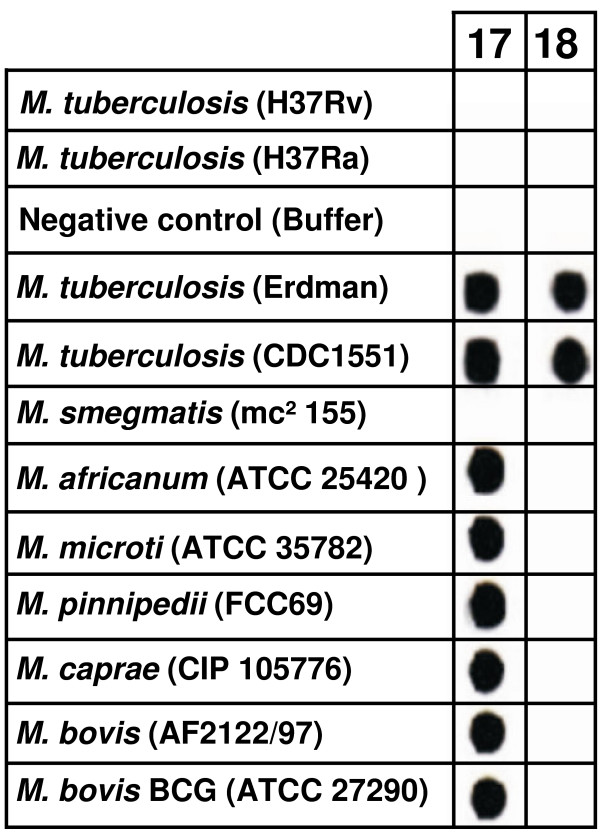
The PE grouping assay (PEGAssay) pattern. Representation of the hybridization pattern results obtained using PEGAssay on selected members of the *M. tuberculosis *complex and other mycobacteria (*M. canettii *and *M. smegmatis*). Lanes 17 and 18 correspond to PE_PGRS17 and PE_PGRS18, respectively. MTBC strains producing a single reacting dot with PE_PGRS 17 (presence of the 12/40 polymorphism in PE_PGRS17 but not in PE_PGRS18) are referred to as "+/-" and belong to the PGRS type 1 group (PGRST1). Isolates whose PEGAssay pattern produces two reacting dots (presence of the 12/40 polymorphism in both PE_PGRS17 and PE_PGRS18 genes) are referred to as "+/+" and belong to the PGRS type 2 genotype (PGRST2). Finally, isolates whose PEGAssay produces no hybridization signal (absence of the 12/40 polymorphism from both PE_PGRS genes) are referred to as "-/-" and belong to the PGRS type 3 genotypic group (PGRST3). Note that *M. smegmatis *strain mc^2 ^155, which lacks both PE_PGRS17 and PE_PGRS18 whole genes, produces no hybridization signal.

In order to be able to rapidly test for the presence or absence of the 12/40 polymorphism, we devised a simple reverse-hybridization-based assay, named the PEGAssay (**PE**_PGRS **G**rouping **A**ssay). This consists of two biotinylated PCR products, encompassing the 12/40 polymophism, each specific to the PE_PGRS17 and PE_PGRS18 sequences from each sample. The labeled amplicons were allowed to react independently with a 5' aminated 24-mer oligonucleotide probe, representing the 12 nt insertion and the 12 downstream nucleotides of the 12/40 polymorphism. Using this assay, we determined the distribution pattern of the 12/40 polymorphism within additional tubercle bacilli strains, known to have a critical position in the evolutionary history of this group of pathogens ("*M. canettii*" and ancestral *M. tuberculosis *strains), and in a larger collection of strains representing the 3 PGG groups recovered from Africa, Europe, North- and South-America. As shown in Figure 5, no hybridization occurred with either *M. tuberculosis *H37Rv or its avirulent derived strain H37Ra, while two hybridization spots are apparent for those strains harboring the 12/40 polymorphism in both PE_PGRS paralogs. As expected, only one reacting dot corresponding to the PE_PGRS17 amplicon could be seen for strains where the 12/40 polymorphism is restricted to this PE_PGRS gene. Compilation of the sequence data with those of the reverse hybridization assay (See Additional file 4) revealed that (i) all "*M. canettii*" strains (n = 6) and one smooth tubercle bacillus isolate are devoid of the 12/40 polymorphism in both PE_PGRS paralogs, (ii) all strains of the *M. africanum*-*M. microti*-*M. bovis *and their related subspecies (n = 98) harbor the polymorphism motif exclusively in their PE_PGRS17 gene sequence (**+/-**), (iii) all PGG1 (ancestral and modern) *M. tuberculosis *strains (n = 108) associate with the *M. africanum*-*M. microti*-*M. bovis *in that they harbor the polymorphism motif exclusively in their PE_PGRS17 gene sequence, (iv) for the remaining modern *M. tuberculosis *strains (259 PGG2 and 48 PGG3), the largest number of strains harbor the polymorphism exclusively in their PE_PGRS17 gene sequence, while a smaller proportion of PGG2 group (n = 112) and only 3 strains from the PGG3 group contained the 12/40 polymorphism in both PE_PGRS17 and PE_PGRS18 genes (+/+). A very small number of isolates (n = 4 PGG2) were found to mirror the reference strain *M. tuberculosis *H37Rv and its derivative H37Ra in that the 12/40 polymorphism motif was absent from both PE_PGRS genes (-/-; n = 6 in total). This finding was confirmed by sequencing (data not shown).

**Figure 6:**
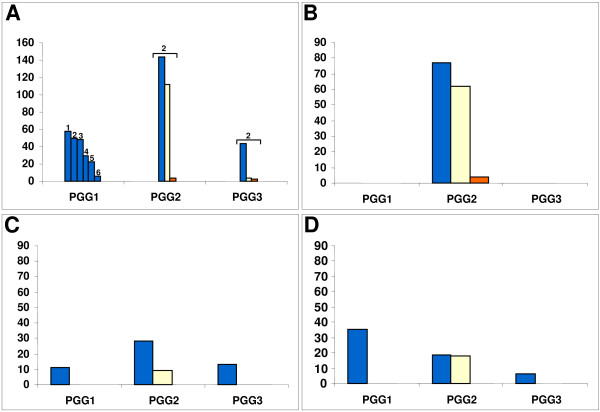
Distribution of the three PE_PGRS-based PGRST groups within the three Principal Genetic Groups (PGGs). (A)- Distribution throughout the whole collection (521 strains), (1)- Ancestral *M. tuberculosis*, (2)- Modern *M. tuberculosis*, (3)- *M. bovis *+ subspecies, (4)- *M. africanum*, (5)- *M. microti *+ subspecies and (6)- "*M. canettii*" (B) Distribution in Tunisian *M. tuberculosis *collection (C) Distribution in American *M. tuberculosis *collection (D) Distribution in South African *M. tuberculosis *collection Blue bars: PGRST1 (+/-), Yellow bars: PGRST2 (+/+), Orange bars: PGRST3 (-/-)

Overall, depending on the occurrence pattern of the 12/40 polymorphism (restricted to PE_PGRS17, shared by both PE_PGRS17 and PE_PGRS18, or absent from both paralogs), the whole MTBC complex falls into three PE_PGRS-based genotypic groups or PGRS types (termed PGRST1 to 3, respectively). Aside from representing all strains (100%) of the *M. africanum*-*M. microti*-*M. bovis *lineage and the *M. tuberculosis *PGG1 group, PGRST1 (+/-) is also significantly the most predominant PGRS type within the PGG2 (56%; *P *< 0.001) and PGG3 (87%; *P *= 0.0033) groups. In contrast, PGRST2 (+/+) and PGRST3 (-/-) are restricted to modern, non PGG1, *M. tuberculosis *strains. The PGRST3 subpopulation is very rare, as in the whole MTBC collection we could only identify 6 strains (1%), which includes the laboratory strains H37Rv and H37Ra. Importantly, PGRST2 was highly associated with PGG2 strains (*P *< 0.001) and could also be significantly found in the PGG3 pool (*P *= 0.0004), albeit at a very low frequency (Figure 6A).

### Frequency of the 3 new PE_PGRS-based genotypic groups (PGRST) in three geographically distinct populations

As our collection contained a substantial number of strains originating from South Africa (Cape Town; n = 61), Tunisia (Tunis, Bizerte, and Zaghouan; n = 144), and the USA (New York and New Jersey; n = 82), we analysed the frequency of PGRST1, 2 and 3 types in these three geographically and socio-economically distinct countries. As shown in Figure (6B, C and 6D), PGRST1 was predominant in the three geographic situations (*P *< 0.001; *P *< 0.001; and *P *= 0.0192, respectively).

### Genetic variation of the PE_PGRS17 and PE_PGRS18 genes

**Figure 7:**
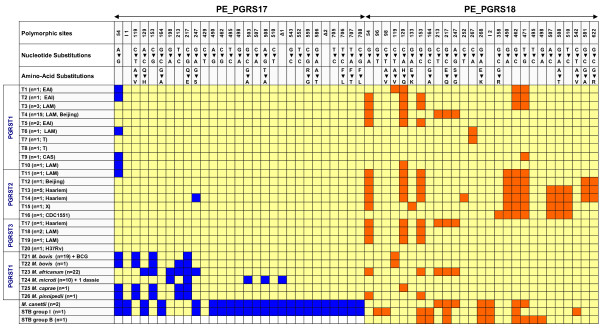
Genetic variability of PE_PGRS17 and PE_PGRS18 genes throughout a worldwide collection of tubercle bacilli isolates. The sequenced region encompasses nucleotides 31 to 712 for both genes (numbering according to the reference strain H37Rv). Sequences of both genes were concatenated and, for MTBC strains, each unique sequence was assigned a type number (T1 to T26). Yellow boxes indicate that the sequence at this site is identical to that of the reference strain *M. tuberculosis *H37Rv. Blue and orange backgrounds correspond to genetic variations (in comparison to H37Rv) within PE_PGRS17 and PE_PGRS18 sequenced regions, respectively. White background indicates no sequence available. STB: Smooth tubercle bacillus. I1: A trinucleotide (GCC) insertion immediately after position 90. I2; A 1-nucleotide (A) frameshift insertion immediately after position 270. Δ1: A 114-nucleotide in frame deletion from positions 512 to 625. Δ2: A 9-nucleotide in frame deletion from positions 631 to 639.

To obtain better insight into the evolution of these two PE_PGRS paralogs, we analyzed the genetic variability of the sequenced genes across the worldwide tubercle bacilli collection. As mentioned earlier, the 12/40 polymorphism is highly conserved, as the same insertion and the 40 accompanying SNPs are observed for both genes irrespective of the MTBC subspecies. In no case, have we noticed a missing SNP or a variation for the 12-nt insertion and its associated 40 SNPs. However, aside from the 12/40 polymorphism, we observed along the partial sequence of both genes, a total of 30 MTBC-associated polymorphic sites (14 sSNPs, 15 nsSNPs and a 114-nt in-frame *M. microti*-specific deletion) (See Figure 7). Overall, after concatenating the sequences of both PE_PGRS genes and taking into account the 12/40 polymorphism, 26 MTBC alleles were identified (T1 to T26), of which 20 (77%) were associated with *M. tuberculosis*. In this species, much of the genetic diversity was found to be associated with PE_PGRS18, as assessed by the estimation of the π value for both synonymous (πs) and nonsynonymous (πa) substitutions. However, both genes appear to be under purifying selection as their ratio of nonsynonymous to synonymous substitutions per site (Ka/Ks) is <1 (Additional file 5). Unlike *M. tuberculosis*, the orthologs from strains of the *M. africanum*-*M. microti*-*M. bovis *lineage appear more homogeneous; the strain collections of *M. africanum *and *M. microti *each defining a unique allele, and sequences for 19 out of the 20 *M. bovis *strains (including *M. bovis *AF2122/97) were identical.

The sequences from the pre-bottleneck species, "*M. canettii" *and other smooth tubercle bacilli, were clearly the most divergent. Indeed, aside from sharing 10 SNPs with the other MTBC strains, they showed 25 additional specific polymorphic sites (15 sSNPs, 7 nsSNPs, 2 insertions, and 1 deletion), clearly indicating their evolutionary distance from the rest of the MTBC. In both "*M. canettii" *and the two other smooth tubercle bacilli strains, PE_PGRS18 is frame-shifted (a 1-nt insertion immediately after position 270) and this gene appears to be much more variable than its paralog.

Further inspection of the nucleotide substitutions revealed that nucleotide changes in both paralogs tend to occur within the same nucleotide positions, irrespective of the evolutionary status of the species. In fact, nucleotide positions 54, 119, 129, 153, 213, 217, 247, 450, 462, 507, 508, and 510, showed variability in both PE_PGRS genes. Thus, certain positions appear to be prone to genetic variation although evolving differently within different species. Strikingly, in all but one variable position (position 119), the mutations in one paralog are permuted comparatively to the other. Consequently, where the mutation is non synonymous (nucleotide positions 129, 217, 247, and 508) the phenotypic change for both paralogs is limited to two amino acids throughout all the species (Figure 7).

Because PE_PGRS genes are GC-rich sequences, we looked at the occurrence of the mutation with respect to the codon position. ANOVA and Tukey's tests (See Additional file 6) showed that, for both genes, the third codon position significantly displayed the highest GC content (*P *< 0,001 for both genes). However, although mutations occurred more frequently at the third codon position (*P *< 0.001 for both ANOVA and Tukey's test) in PE_PGRS18, no such association could be observed for PE_PGRS17. Thus, a mutational bias might have operated for the diversification of PE_PGRS18.

## Discussion

Previous studies involving comparative genomics and explorative genome-wide multilocus analysis conclusively showed that the present MTBC strains appear as a genetically homogeneous clonal pool, since they display a highly significant linkage disequilibrium and an exceptionally low rate of silent nucleotide substitutions [4,7,33,35-37]. This picture contrasts with the situation that seems to have prevailed in the very early history of the tubercle bacillus in which a significant rate of DNA exchanges were allowed, most likely through intragenomic recombination and horizontal gene transfer [19]. Compelling evidence suggest that members of the MTBC arose from a single successful ancestor, resulting from a recent evolutionary bottleneck [4,15,19]. The identity of such a parental strain has not been defined, though some genetic markers (polymorphisms located in codon 463 of the *katG *gene and codon 95 of the *gyrA *sequence, an SNP in the promoter region of the *narGHJI *gene complex, and the TbD1 deletion) help to distinguish between ancestral and modern MTBC strains [4,15,16,38].

The findings from this study provide additional evidence for the concept that the present clonal MTBC strains are the progeny of a single successful ancestor. Indeed, the identified PE_PGRS-associated 12/40 polymorphism could represent a genetic marker for the most successful post-bottleneck-derived clone from which the MTBC strains expanded. Based on this polymorphism, we showed that all MTBC strains could be assigned to three new PE_PGRS-based genotypic groups (PGRST1 to 3). Strikingly, PGRST1 was found to be predominant in all three *katG*-*gyrA *defined PGG groups irrespective of their geographic origin and evolutionary status (ancestral or modern). Because all ancestral (TbD1+) strains, including the *M. africanum*-*M. microti*-*M. bovis *lineage, belong to PGRST1, one can argue that acquisition of the 12/40 polymorphism is coincident with the emergence of the most successful MTBC parental strain. Consistently, the 12/40 polymorphism was absent from both PE_PGRS genes in 6 "*M. canettii*" strains and one other smooth tubercle bacillus analyzed, which are believed to represent the very early pre-bottleneck MTBC progenitors [19].

**Figure 8:**
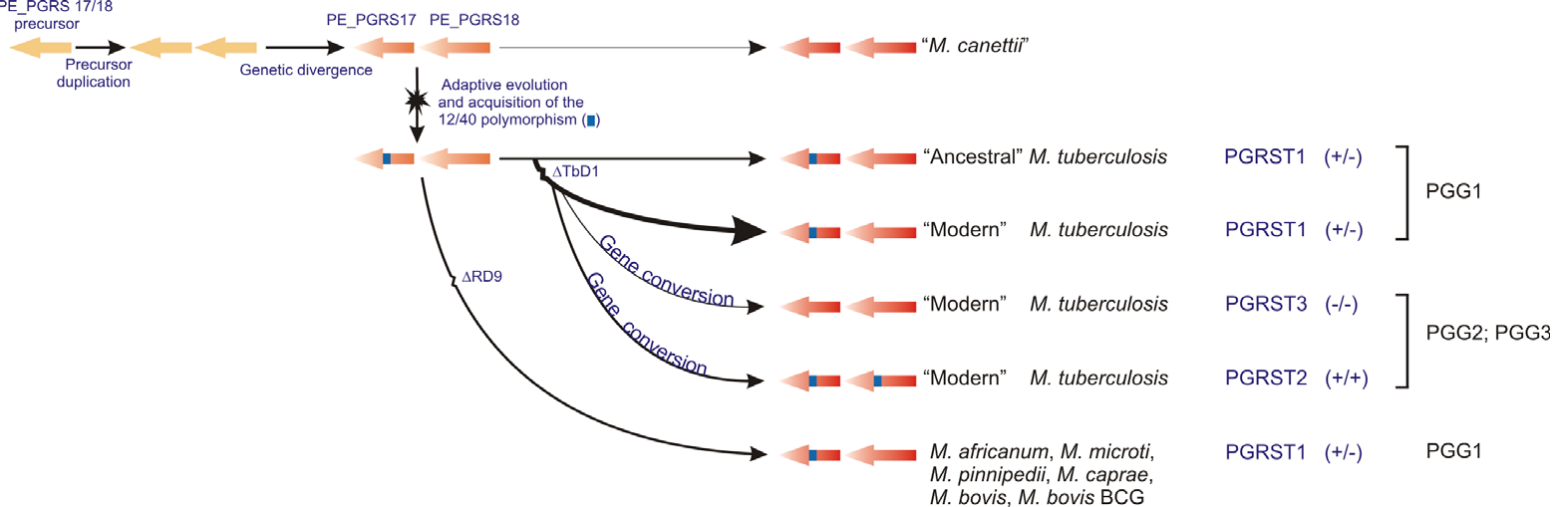
Schematic representation of the proposed evolutionary history of the tubercle bacilli according to the presence and absence of the 12/40 polymorphism. The scenario was constructed from the distribution of the 12/40 polymorphism within and between species. This scenario fits in with previously-proposed evolutionary schemes based on deletion regions and single nucleotide polymorphisms [15, 15, 19]

From the overall distribution pattern of the 12/40 polymorphism, a general evolutionary picture emerges (Figure 8) which conforms to, and confirms, previously published evolutionary scenarios for the tubercle bacillus [4,15,16,19]. As PE_PGRS17 and PE_PGRS18 showed the highest degree of homology and represent a typical tandem duplicate, one can deduce the molecular events which took place in the most ancestral tubercle bacillus strain. Such a strain would be TbD1+ and harbor a single copy of a gene genetically very close to PE_PGRS17 or PE_PGRS18 (the PE_PGRS17/18 common ancestor). After duplication of this ancestral gene copy, further gene evolution led to the "*M. canettii*"-associated genetic configuration in which both genes lack the 12/40 polymorphism, but have separately undergone isolated single nucleotide changes. During the adaptive period of the progenitor species, additional genetic changes took place, including the occurrence of the 12/40 polymorphism in the one paralog (PE_PGRS17). The fact that the GC content of the 12/40 polymorphism is in the range of that of the mycobacterial host genome, may have ensured its maintenance throughout the clonal expansion of the MTBC progenitor. The data suggest that the polymorphism has remained stable throughout the expansion into the *M. africanum*-*M. microti*-*M. bovis *lineage and within the ancestral *M. tuberculosis *strains, and only with the emergence of modern TB has this variation been able to be transmitted (in its entirety) into the other paralog or deleted from PE_PGRS17. Such a genetic rearrangement occurred exclusively in PGG2 and PGG3 groups leading to the emergence of the two aforementioned disproportionately less frequent PGRST2 and PGRST3 subpopulations.

The emergence of these two newly defined modern subpopulations could be explained by the natural occurrence of homologous recombination between the two PE_PGRS gene sequences of strains from the PGRST1 population. The sequence environment (close proximity and high sequence identity) appears to be optimal for such a mechanism to take place, although it only seems to occur in the modern *M. tuberculosis *strains. It has already been shown, through *in vitro *experiments, that initiation of homologous recombination occurs in mycobacteria provided that sequence heterology does not exceed 10–12% [39]. Such a requirement is largely fulfilled in the case of PE_PGRS17 and PE_PGRS18, but may eventually restrict homologous recombination events between other PE_PGRS paralogs that have undergone substantial levels of sequence divergence (up to 12%).

Transfer of the 12/40 polymorphism from the PE_PGRS17 to its neighboring paralog, or its reversion (loss from PE_PGRS17 sequence) in modern *M. tuberculosis*, is typical of a homologous recombination process called "gene conversion". Such a gene replacement event is frequently observed among members of multigene families in bacterial genomes and contributes to both the maintenance of genetic information and creation of genetic diversity [40]. It is very unlikely that horizontal gene transfer (HGT) may have contributed to generate the two modern PGRS types of populations (PGRST2 and PGRST3). Indeed, random acquisition of the 12/40 polymorphism through HGT would have generated an additional PGRS type population, harboring the polymorphism uniquely in its PE_PGRS18 sequence (a putative "-/+" population).

The recent generation of PGRST2 and PGRST3 subpopulations from the predominant PGRST1 accommodates either the double-strand break repair (DSBR) or the synthesis-dependent strand annealing models [41,42] as the molecular basis for this gene conversion event. It seems that the pre-synaptic double-strand breaks that initiate the homologous recombination event in the PGRST1 population occurred more frequently in PE_PGRS18 than in PE_PGRS17, so that PGRST2 emerges more frequently than PGRST3. Under such circumstances, the DNA polymerase will use the 12/40 polymorphism-containing PE_PGRS17 sequence as homologous template to fill-in the broken PE_PGRS18 gene sequence, resulting in the PGRST2-associated genotype.

**Figure 9:**
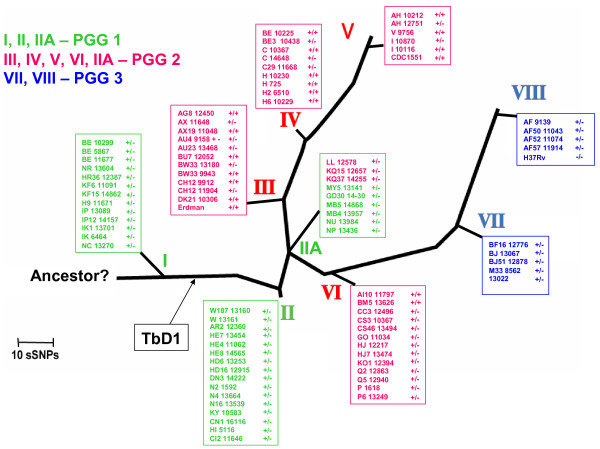
Distribution of the three PGRS types through sSNP-based genetic clusters and PGG groups. The Phylogenetic tree of *M. tuberculosis *isolates from New York and New Jersey shows the relative distribution of PGRS types 1 (PGRST1; +/-), 2 (PGRST2; +/+), and 3 (PGRST3; -/-) with respect to the synonymous single-nucleotide (sSNP)-defined 9 major genetic clusters (I, II, IIA, and III to VIII) and PGG grouping. The 36 sSNP-based phylogenetic tree was constructed as described by Gutacker *et al*. [33]. The data clearly indicate that the PE_PGRS17/PE_PGRS18-associated gene conversion event occurs multiple times mainly in PGG2 strains.

The distribution of the polymorphism types when informed by PGG and sSNP clusters strongly indicates that gene conversion events (followed by clonal expansion) occurred independently multiple times (Figure 9). Indeed, all the PGG2 sSNP clusters each possess both PGRST1 and PGRST2 despite existing over clearly divergent evolutionary tracts. Based upon the data set presented in Figure 9, conversion has occurred at least four times and reversion at least once (or twice if one considers the PGG2 PGRST3 strains not included in Figure 9). Overall, the results clearly indicate that PGRST2 and PGRST3 subpopulations are modern *M. tuberculosis *lineages and emerged separately from PGRST1 strains through multiple gene conversion events. It is also interesting to note that two of the 6 strains identified with PGRST3 were laboratory strains (H37Rv and H37Ra), which have undergone significant *in vitro *culturing over decades, so that this conversion may not really occur or be tolerated well only under *in vivo *infection conditions.

RecA-mediated gene conversion processes have been shown to occur *in vitro *between two rRNA operon copies in *M. smegmatis*, uncovering the molecular mechanism underlying resistance to aminoglycosides [43]. As far as could be ascertained, this study provides the first concrete example and the most direct evidence for the natural occurrence of gene conversion events in mycobacteria. However, Gutacker et al. [33] have previously suspected recombination when addressing the distribution pattern of 5 polymorphic nucleotides within the Rv0980c-Rv0981 intergenic region (Rv0980c-Rv0981 iSNPs). This finding raises the question whether the complicated pattern of the Rv0980c-Rv0981 iSNPs would be linked to the 12/40 polymorphism-associated gene conversion event. Indeed, if gene conversion extends to the homologous intergenic sequences of PE_PGRS17 and PE_PGRS18, the distribution profile of the iSNPs must be identical for both genes, as a result of gene replacement. The data show, that for both H37Rv and CDC1551, whose PE_PGRS17 and PE_PGRS18 genes have undergone gene conversion, the iSNPs distribution patterns of the two genes are quite different. Thus, unless the 5 polymorphic nucleotide positions are exceptionally unstable, the complicated pattern of this intergenic polymorphism does not seem to be associated with the gene conversion event described in the present paper.

It is well assumed that gene duplication and subsequent functional divergence are crucial for bacterial evolution as they play a major role in gene innovation and adaptation to changing environments [32]. In this context, it is worth mentioning that although PE_PGRS genes are restricted to mycobacterial species, they have preferentially expanded within the genomes of pathogenic mycobacteria, most likely through extensive gene duplication events coupled to genetic divergence during their adaptation to the very hostile intra-macrophagic environment [24]. We hypothesize that gene conversion may have contributed to the evolution of members of the PE_PGRS subfamily and may have participated in the generation of antigenic variation in their members [22,2,28]. It is striking that this type of recombination does not seem to occur in the MTBC members other than modern *M. tuberculosis*, and one wonders whether this is a mechanism that is specific to, or that may occur at greater frequency in modern *M. tuberculosis*. Recently, Lui *et al*. [44], extending Gutacker's analysis [33], identified a mosaic polymorphic pattern (the IRMT0105 locus) associated with a PPE gene (MT0105). The authors hypothesize that small-scale gene conversion or recombination at hotspots near PE or PPE gene families has been an important mechanism for *M. tuberculosis *to escape immune surveillance.

As far as could be ascertained, the functions(s) of PE_PGRS17 and PE_PGRS18 are unknown and, as yet, there is no indication whether they are essential or not. According to the present study, it could be assumed that both PE_PGRS genes may be dispensable for normal *in vivo *growth under certain conditions as they are absent from the genome of *M. leprae*, and PE_PGRS18 was frame-shifted in the two "*M. canettii*" and two other smooth tubercle bacilli strains analysed. By contrast, no such frame shift mutations were observed in the worldwide sequenced collection of MTBC strains, indicating that they may have evolved to assume an essential role in these particular widespread species. Consistently, PE_PGRS17 and PE_PGRS18 belong to the so-called *iVEGI *(*in vivo*-expressed genomic island), a cluster of 49 *in vivo*-expressed genes, thought to encode cell wall components and participate in lipid metabolism required for mycobacterial survival *in vivo *[45]. Within this island, PE_PGRS17 and PE_PGRS18 account among the 21 genes that display higher expression levels in mice samples compared to *in vitro *cultures. The *iVEGI *locus harbors at least three genes (Rv0981, Rv0986, and Rv0987), whose products were shown to be required in early interactions with the host cell as well as in persistence [46-48]. Furthermore, genes playing critical roles in bacterial survival and fitness generally display higher acquisition rates for sSNP (Ks) in comparison to nsSNP (Ka). We found that both PE_PGRS17 and PE_PGRS18 are under purifying selection as the majority of disadvantageous phenotypic changes would have been eliminated during evolution. We consistently found that, irrespective of the species, only particular non-synonymous changes are tolerated within certain nucleotide positions of both genes. These findings, and the fact that these genes appear to be preferentially expressed *in vivo *[45], argue for a potential role in host-pathogen interactions.

Finally, the question comes to mind whether the occurrence of the 12/40 polymorphism could have enabled PE_PGRS17 to acquire a new or altered function that positively influenced the evolution of the MTBC. If so, it is also interesting to speculate whether the recent change from the PGRST1 to PGRST2 genotype in *M. tuberculosis *would further increase its fitness and/or adaptability. Indeed, as with PGG2 isolates, PGRST2 appears to be frequently associated with outbreak strains and clustered cases, although not exclusively. By contrast, as mentioned earlier, the PGRST3 genotype is very rare among TB cases, and two of the four strains found to contain the genotype are in fact laboratory strains (H37Rv and H37Ra), thus seeming less prone to expansion and/or to have occurred as a more recent reversion event. Although it is hard to believe that a single polymorphism in one or two genes would have dramatically impacted the evolution of the tubercle mycobacterial species, further experiments based on gene replacement and/or inactivation of the different forms of both PE_PGRS genes are needed to clarify this issue. It is worth mentioning that the 12/40 polymorphism lies within a region of the protein, which according to the domain organization proposed by Brenan *et al*. [25], may represent a transmembrane helix. This location may be critical for the protein function inasmuch as PE/PPE protein complexes are strongly suspected to be involved in signal transduction [49].

## Conclusion

Deciphering the evolution of bacterial populations is crucial to better understand the genetic traits behind the emergence of biomedically relevant strains. In the present study, we identified a novel, PE_PGRS-based, genetic polymorphism that expands our knowledge of the history of the tubercle mycobacterial species. This polymorphism provides a valuable marker of the ill-defined successful ancestor that emerged from the evolutionary bottleneck and from which the MTBC expanded. The findings also demonstrate the involvement of natural gene conversion events specifically in the diversification of the modern *M. tuberculosis *population. To our knowledge, this paper provides the first concrete example for the natural occurrence of such a molecular event in mycobacteria.

## Methods

### Sequence data

The complete gene and protein sequences of all members of the PE gene family in the genome of *M. tuberculosis *H37Rv were obtained from the GenoList (Pasteur Institute) website [50]. The sequences from all PE members which are contiguous in the genome were extracted from these datasets for further characterization. The contiguous PE sequences were aligned using CLUSTALW [51]. Amino acid similarity and identity rates were calculated using BioEdit [52] with an integrated Blosum62 matrix. Members that showed high percentages of identity were further aligned to their corresponding orthologs from the genome sequences of *M. tuberculosis *CDC1551 [53] and *M. bovis *AF2122/97 [50]. The complete genome sequences of *M. marinum *strain ATCC BAA-535 [54] and *M. microti *strain OV254 [54] were obtained from the Sanger Institute website.

### Bacterial strains

A total of 521 mycobacterial isolates recovered from diverse geographic origins (Africa, Asia, Australia, Europe, and North- and South-America), were used in this study. The collection was chosen to be representative of the known diversity of the MTBC and the pre-bottleneck lineages. It included 415 *M. tuberculosis *strains (representing members from all three Principal Genetic Groups (PGG) as defined by Sreevatsan et al. [4], i.e. 108 PGG1, 259 PGG2 and, 48 PGG3 strains), 42 *M. bovis *strains (including 5 different BCG strains), 30 *M. africanum *strains representing members from all three subtypes defined by Viana-Niero et al. [55] (i.e. 14 subtype A1, 6 subtype A2 and 8 subtype A3 strains), 17 *M. microti *strains (including 9 from voles, 3 from llama, 2 from cat, 1 from pig and 2 from humans), 3 dassie bacillus strains, 4 *M. pinnipedii *strains, 2 *M. caprae *strains, 6 "*M. canettii*" strains and 2 smooth tubercle bacilli isolates (representing members from five of the eight Smooth Tubercle Bacilli Groups- ST groups-identified by Gutierrez et al. [19], ie.1 ST group A, 3 ST group C, 2 ST group D, and 1 each of ST group B and I). The *M. tuberculosis *isolates, which were recovered from at least 32 different countries, involved 57 ancestral (TbD1+) [15], 31 Beijing, 91 Haarlem, 73 LAM, 85 T, 16 CAS, 17 X, 5 S, 10 U and 1 MANU families [56]. Details on the geographic origin, host, spoligotype pattern, and PGG of each isolate of the whole collection are available in the Additional file 4.

### PCR and DNA sequencing

PCR amplification of PE_PGRS17 (Rv0978c) and PE_PGRS18 (Rv0980c) gene sequences was accomplished using a common sense primer, 7880S (5'-ATGTCGTTTGTCAACGTGGC-3'; positions 1–20) and the specific reverse oligonucleotides 0978R1 (5'-TCAGCTGATTACCGACACCGT-3', 976–996) and 0980R1 (5'-TCATATGGCCGCCGAACACAC-3', 1354–1374), respectively. The amplification reaction mixture contained 2 μl of template genomic DNA (about 20 ng), 10 μl of 10× buffer (Qiagen), 10 μl DMSO, 2 μl of 10 mM nucleotide mix (Amersham Biosciences), 2 μl of each primer (20 μM stock), 0,25 μl (1.25 U) of HotStart *Taq *DNA polymerase (Qiagen) and sterile nuclease-free water (Amersham Biosciences) to 100 μl total reaction volume. Cycling was carried out in a PTC 9700 thermocycler (Applied Biosystems) with an initial denaturation step of 10 min at 96°C followed by 35 cycles consisting of 1 min at 95°C, 1 min at 60°C and 2 min at 72°C. The amplification ended with a final elongation step of 7 min at 72°C. PCR products were purified using the GFX PCR DNA and Gel Band purification kit (Amersham Biosciences) according to the manufacturer's protocol. Partial DNA sequencing (nucleotides 31 to 712) was performed using the sense primers 7880S (see above) and PEGA.S (5'-CAAGCGATCAGCGCGCAGG-3', 184–202) for both genes. Sequencing on the reverse strand involved the internal primers 0978R2 (5'-CGCTTGGACCGTTGCCGATGG-3', 770–790) and 0980R2 (5'-GAGGCTGACCGCGCCGCCGGT-3', 730–750) for PE_PGRS17 and PE_PGRS18, respectively. Determination of the nucleotide sequence was performed with the Prism Ready Reaction Dye Deoxy Terminator Cycle sequencing Kit on an ABI PRISM 377 DNA sequencer (Applied Biosystems). Each sample was sequenced from two independent PCR amplification reactions.

### Sequence analysis

The sequence data was edited and aligned using the software programs BioEdit [52] and ClustalW [51]. Both the PE_PGRS17 and PE_PGRS18 genes were either analysed individually or upon concatenation. The software programs Arlequin v.2.0 [57] and DNASP [58] were used to obtain summary statistics of genetic diversity. To test for adaptive selection, we determined the nucleotide substitution changes and the ratio of synonymous (Ks) and nonsynonymous (Ka) substitutions per site. For this purpose, we used the analysis developed by Nei-Gojobori [59] as implemented in the DNASP package. A statistical analysis of ANOVA and a Tukey's test were performed to test for significant difference of GC content and substitution rates between PE_PGRS17 and PE_PGRS18.

### Set up of a reverse hybridization dot blot assay for rapid PE_PGRS-based grouping (PEGAssay) of MTBC strains

Biotinylated PCR products encompassing the 12/40 polymorphism were obtained using the common sense primer PEGA.S and the biotinylated member specific reverse primers PEGA78.R (5'-bGACACCGTGCCGCTGCCGAAA-3', 705–725) and PEGA80.R (5'-bCCGTTGCCGAACAGCCATCC-3', 568–587) for PE_PGRS17 and PE_PGRS18, respectively. The amplification conditions were the same as mentioned above. Ten microliters of the biotinylated and heat denatured PCR product (diluted to a total volume of 150 μl with 2 × SSPE-0.1% SDS) was further hybridized with a 24-mer 5' amino-linked oligonucleotide probe (5'-GATCGAGCAGGCCCTGTTGGGGGT-3'). The latter represents the 12-nucleotide insertion and the immediate downstream 12 nucleotides of the 12/40 polymorphism and was synthesized according to the PE_PGRS17 sequence of strain CDC1551. The probe was diluted in 150 μl of 0.5 M NaHCO_3 _(final concentration of 3 ng/μl) and covalently bound to a Biodyne C membrane (Pall Biosupport, Portsmouth, United Kingdom) using standard protocols [60]. Briefly, after activation of the membrane, 150 μl of the diluted probe was applied in wells of a 96-well dot blot apparatus (BioRad) and incubated for 5 min at room temperature. Following inactivation and washing steps, biotinylated PCR products were added to the wells and the entire apparatus was then incubated at stringent hybridization conditions (65°C for 1 h). The samples were removed by vacuum aspiration and the membrane was washed three times with 50 ml of 2 × SSPE-0.5% SDS for 10 min at 57°C. After incubation for 45 min at 42°C with 10 U of streptavidin-POD (Amersham Biosciences) diluted in 20 ml of 2 × SSPE-0.5% SDS, the membrane was washed twice with 50 ml of 2 × SSPE-0.5% SDS for 10 min at 42°C and then once with 25 ml of 2 × SSPE for 10 min at room temperature. ECL chemiluminescence detection reagents (Amersham Biosciences) were added according to the manufacturer's instructions, and the membrane was exposed to Hyperfilm ECL (Amersham Biosciences) for 5 min. To allow for repeated use (up to 10 times), the membrane was stripped during a 1 h incubation in 1% SDS at 90°C, after which it was incubated in 20 mM EDTA for 20 min at room temperature and stored at 4°C.

### Typing assays

Spoligotyping, assignment of isolates to PGG, and detection of the TbD1 region, were performed according to previously described protocols [60,4,15].

### Statistical analysis

Associations were evaluated for statistical significance using the χ2 or Fisher's Exact Test implemented in GrapPad Prism v.4. (GraphPad Software, Inc., USA). A *P *value < 0.05 was considered to be significant.

## Abbreviations

PE_PGRS, Proline-glutamic acid_polymorphic GC-rich repetitive sequence; nsSNP, non-synonymous single nucleotide polymorphism; sSNP, synonymous single nucleotide polymorphism; PGG, Principal Genetic group; PGRST, PGRS type

## Authors' contributions

**AK**: Sequencing and computational analysis, set up and performed reverse hybridization experiments (PEGAssay), typing, and manuscript preparation

**NCGP**: Comparative genomics analysis, sequencing and typing of South African *M. tuberculosis *strains, and manuscript preparation

**AN**: Statistical analysis and typing experiments

**VV**: Provided a worldwide MTBC strains collection including proTB strains and made helpful comments during the project progress

**CS**: Provided ancestral MTBC strains, helped with typing and strain families classification, and made helpful comments with regard to the evolutionary scenario

**NR**: Provided ancestral MTBC strains and made helpful comments

**PS**: Enriched the strain collection with Brazilian *M. tuberculosis *and *M. bovis *strains, helped with typing and provided comments

**MF**: provided proTB strains and helped with typing

**AC**: Enriched the strain collection, typing, and comments

**RCH**: Provided a consistent and well characterized *M. tuberculosis *strain collection from New York and New Jersey, participated actively in the manuscript correction

**NK**: Performed typing and SNPs-based phylogenetic analysis of *M. tuberculosis *strains from New York and New Jersey. Also participated to the correction of the manuscript.

**BK**: Provided a well characterized worldwide *Mycobacterium tuberculosis *strain collection with a significant number of strains from New York and New Jersey, and made helpful comments and suggestions during the work progress

**JLH**: Provided a well characterized and typed *Mycobacterium tuberculosis *strain collection from New York and New Jersey, shared some results of his work before publishing and made helpful comments and suggestions during the work progress

**CG**: Provided a worldwide strain collection of MTBC including proTB strains and made helpful comments and suggestions during the work progress

**HM**: Project leader, project guide and in-charge of manuscript preparation, final corrections and submission

All authors read and approved the final version of the manuscript.

## Supplementary Material

Additional file 1 Percent similarity (identity) values of the PE deduced amino acid sequences (whole gene, PE and PGRS regions) that are contiguous in the genome of *M. tuberculosis *strain H37Rv. The percent values were calculated using the BioEdit program [52].*According to Gevers et al. [32]. NA: not applicable (the gene sequence is too short, and the junction between the PE and PGRS regions is not well delimited). Click here for file

Additional file 3 Nucleotide sequence alignments of *M. tuberculosis *(H37Rv) genomic relevant regions extending from Rv0976c to Rv0981 with their corresponding sequences in *M. marinum *(ATCC BAA-535), *M. ulcerans *(Agy99), and *M. avium *subspecies *paratuberculosis *(*Map *K-10). Note the absence in the latter species of gene sequences homologous to PE_PGRS16, PE_PGRS17, and PE_PGRS18. The *M. tuberculosis *Rv0980c-Rv0981 intergenic region that contains the iSNP polymorphism described by Gutacker et al. [33] has no homolog in *M. marinum*. Click here for file

Additional file 4 Characteristics of the whole collection of tubercle bacilli isolates used in this study. Lines in yellow indicate the 98 isolates, whose PE_PGRS17 and PE_PGRS18 sequences (nucleotide positions + 31 to +712) were subjected to sequencing. *According the Brudey et al. [56] §According to Gutierrez et al. [19] Click here for file

Additional file 5 Summary statistics of the genetic diversity within PE_PGRS17 and PE_PGRS18 sequenced regions. The statistics were generated using the software programs Arlequin v.2.0 [57] and DNASP [58]. M. tb: *M. tuberculosis*; M. afr: *M. africanum*; M. mic: *M. microti*; M. bov: *M. bovis*. The subspecies include *M. caprae*, *M. pinnipedii*, and dassie bacillus. M. can: *M. canettii*; STB: smooth tubercle bacillus. s: synonymous; a: nonsynonymous; NA: not applicable. Click here for file

Additional file 6 Statistical analysis of ANOVA and a Tukey's test used to test for significant difference of the GC content and substitution rates within the three codon positions for both PE_PGRS17 and PE_PGRS18 genes. For this purpose, each unique sequence for each gene was assigned a different number (ST1 to ST10 and ST1 to ST19 for PE_PGRS17 and PE_PGRS 18, respectively). Click here for file

Additional file 2 Schematic representation of the genes (shown by arrows) and gene order of the genomic region containing PE_GRS17 and PE_PGRS18 genes. (A) Comaprison of *M. tuberculosis *with *M. marinum*, *M. ulcerans*, and *M. avium *subspecies *paratuberculosis*. (B) Comparison between *M. tuberculosis *and *M. leprae*. Shaded areas indicate homologous regions. Note the inversion of the region encompassing echA7 to fadE13 between the two latter species. Click here for file
