# Supplementary material for: Insights into the evolutionary history of tubercle bacilli as disclosed by genetic rearrangements within a PE_PGRS duplicated gene pair
Source: BMC Evol Biol. 2006 Dec 12;6:107. doi: 10.1186/1471-2148-6-107 (PMC1762029; doi:10.1186/1471-2148-6-107)
Supplement: Additional file 1 — Percent similarity (identity) values of the PE deduced amino acid sequences (whole gene, PE and PGRS regions) that are contiguous in the genome of M. tuberculosis strain H37Rv. The percent values were calculated using the BioEdit program [52].*According to Gevers et al. [32]. NA: not applicable (the gene sequence is too short, and the junction between the PE and PGRS regions is not well delimited). [file 1471-2148-6-107-S1.pdf]

Gene designation	Size (bp)	% of amino acid similarity (identity)			Number of alignable stretches*
		PE	PGRS	Whole gene	
PE1 (1767 bp)<br>PE2 (1578 bp)	1767<br>1578	25 (15)	NA	51 (42)	0
PE3 (1407 bp)<br>PE4 (1509 bp)	1407<br>1509	68 (52)	NA	67 (53)	0
PE_PGRS12 (414 bp)	414				
		NA	NA	8 (7)	0
PE_PGRS13 (2250 bp)	2250				
		25 (17)	50 (45)	39 (34)	0
PE_PGRS14 (2649 bp)	2649				
		NA	NA	11 (9)	0
PE_PGRS12 (414 bp)					
PE_PGRS16 (2772 bp)	2772				
		58 (46)	18 (15)	22 (18)	0
PE_PGRS17 (996 bp)	996				
		98 (97)	59 (57)	68 (67)	2
PE_PGRS18 (1374 bp)	1374				
		57 (44)	27 (23)	23 (29)	0
PE_PGRS16 (2772 bp)					
PE_PGRS19 (2004 bp)<br>PE_PGRS20 (1392 bp)	2004<br>1392	40 (027)	56 (53)	59 (54)	1
PE9 (435 bp)<br>PE10 (363 bp)	435<br>363	NA	NA	26 (16)	0
PE_PGRS21 (2304 bp)<br>PE_PGRS22 (2562 bp)	2304<br>2562	88 (82)	61 (53)	63 (58)	1
PE_PGRS27 (3990 bp)<br>PE_PGRS27 (2226 bp)	3990<br>2226	96 (95)	41 (39)	46 (44)	1
PE_PGRS36 (1305 bp)<br>PE21 (174 bp)	1305<br>174	NA	NA	6 (3)	0
PE_PGRS42 (2085 bp)<br>PE_PGRS43 (4983 bp)	2085<br>4983	78 (69)	26 (24)	29 (27)	0
PE_PGRS49 (1455 bp)<br>PE_PGRS50 (4617 bp)	1455<br>4617	22 (18)	20 (18)	24 (22)	0
PE_PGRS53 (4146 bp)<br>PE_PGRS54 (5706 bp)	4146<br>5706	74 (54)	47 (42)	48 (43)	0
PE_PGRS55 (4146 bp)	2145				
		22 (13)	42 (38)	40 (35)	0
PE_PGRS56 (3240 bp)	3240				
		25 (15)	49 (45)	47 (43)	0
PE_PGRS57 (4470 bp)	4470				
		86 (76)	32 (30)	36 (33)	0
PE_PGRS53 (4146 bp)					
PE_PGRS60 (4146 bp)<br>PE_PGRS61 (4146 bp)	315<br>588	NA	NA	15 (11)	0
