# Supplementary material for: Insights into the evolutionary history of tubercle bacilli as disclosed by genetic rearrangements within a PE_PGRS duplicated gene pair
Source: BMC Evol Biol. 2006 Dec 12;6:107. doi: 10.1186/1471-2148-6-107 (PMC1762029; doi:10.1186/1471-2148-6-107)
Supplement: Additional file 3 — Nucleotide sequence alignments of M. tuberculosis (H37Rv) genomic relevant regions extending from Rv0976c to Rv0981 with their corresponding sequences in M. marinum (ATCC BAA-535), M. ulcerans (Agy99), and M. avium subspecies paratuberculosis (Map K-10). Note the absence in the latter species of gene sequences homologous to PE_PGRS16, PE_PGRS17, and PE_PGRS18. The M. tuberculosis Rv0980c-Rv0981 intergenic region that contains the iSNP polymorphism described by Gutacker et al. [33] has no homolog in M. marinum. [file 1471-2148-6-107-S3.pdf]

M. marinum orthologue of Rv0981

tcagggtggcgtttcgcgtagcacgtaacccaccccgcgcacggtgtggatcagccgcggctcgccctcggcctcggttttacgccgcaggtagcccacgtagacctcgagcgcgttgccggacgtgggaaagtcg
aagccccacacctcttcgaggatgcggctgcgggtcagcacccgtcgcgggttggcgatcagcatctccagcagggcgaactcggttcgggtgaggctgatgcgccgcgccccgcgggtcacctcccgggtcaccg
ggtccaggctcaggtcggagaacgtcatggctaccgaatcggcggcttcgtcgtcttcgggcttggtgcggcgcagcaacgcgcgcatccgcgccaacagctcttccagcgcgaacggcttcggcaggtagtcatc
cgcgccggcatcgagccccgcgacccgttcggagacggaatcacgggcggtcagcacgagaatgggcagatcatcaccggtactgcgaagttgacggcatacctccaagccatccagtcgcggcatcatgacgtcc
agcaccaacgcgtcaggccgatcactggcaatcatctcgagagcttccacaccatcgtgtgccagctcaaccgtgtagccattgaaggaaagcgacctgcgcagcgactcgcgcaccgcgcgatcgtcgtcgacga
caagtatccgcacggacat
tagtttcgtcctaacgcctgagagcggcctgagaggcgcgccgaaatgacgacagcgtcacaatttgatcgggatcaataaccaacggtttgccagcgccgccgggcagccggcatcgatgcacggcacggccgtc
atgccgtgccgtatcacaaccgccacattggccccaccgggaaggggccgggttgcgccctctcagcagcgacggcagtcgccgctaagaggacagagacacccaggcgcgatggcccagggtaatcggg

M. marinum orthologue of rpmF

ctatcgacggtcgagatcgatgagaccgagccgggccgccttgagcagccggcgaggcaccttgtgcttctgaccggcaacggtgacgccgacgagttcggccttggtggccttccactgcgagcgccggcttcga
gtattcgcgcgcgacatcctgcgctttggtgtggccat
ggtcggacctaactcctcggtcggggtttcgggttcaacatgtcgcacggcatggccaatcggcacagcggcgacgaatgacgtaaacgatagtcggtgacctgctggttgccaaaaccgggcaccgccggatccg
ccgtgttcacgtaccgagtgaggcgggtatcacaccgtggcaggcgtcccgctatgctgacctaccggttggttggttcacccaccggcaagccctgcccaccggtaaccccgatgaaggaggacgcggatggcgt
ctcgttctgatgcgtccggcgcc

M. marinum orthologue of Rv0976c

gtacgcatcgcgaactgttccggcttctacggggaccgtctctcggcgatgcgcgaaatgctcaccggcggcgaggtggactacctcaccggcgactacctcgccgaactgaccatgctgatcctgggccgcgacc
ggatgaaaaaccccgagcgcggctacgccaagaccttcctggcccagctcgaagactgcctgggactggcccacgagcgcggagtgcgcatcgtcgccaacgccggcggcctcaatcccgccgggctggccgatgc
gatcagggccctaaccgagcgcttgggcatcggcgcccgcatcgcccacgtggaaggcgacgatctggcgccccgcgcggccgaactcggcctgggcacaccgctgacagccaacgcctacctgggcgcgtggggc
atcgtcgattgcctcaacagcggggccgacgtcgtggtcaccggccgggtcaccgacgcctcggtgatcgtcggatccgcggcggcgcacttcggctggggccgcaccgactacaaccagctggccggcgccgtgg
tggccggccatgtcatcgaatgcggggtccaggccaccggcggcaactacgccttcttcaccgaggtccccgatctgacctacgccggcttcccgctggccgaggtcagcgccgacggcacctcggtgatcaccaa
acaccccggcaccggtgggctggtcagccccgacaccgtcaccgcacaactgctctacgagatatccggcgcccgctacgccaatccggacgtgacggcccggatggacaccatccaactgtccagcgatggcccg
gatcgggtgcgcatcagcggcgtgatcggcgaagcccccccacccaccctgaaggtgtcgctgaacagcatcggcgggttccgcaacgccatgacgttcgtcttgacgggtctgaacatcgaggccaaggccgagc
tcgtgcaacgccagctcgaggccaccctgaccgtcaaacccgccgaactggaatggaccctggcccgcaccgaccacgtcgacgccgacaccgaggaagccgccagcgccctgctgcactgcgtggtccgtgaccc
cgaccccgccaatgtgggccgcaagttctcctcggccgcggtggaattggcgttggccagctatcccggattcacctcgaccgcaccccgggcgacggccaggtgtatggggtgttcgtgcccggctacgtggac
gccaagctggtgccacacatcgccgtgcacgccgacggcacgcgcaccgatatcccctgcgccaccgagaccctggaactggaatcggtcgatcccccggccttgcccgatccgctgcccaccggcccgacccgac
gggtaccgctgggcctgattgccggcgcccgcagcggggacaagggcgggtcggccaacgtcggactatgggtgcgcaccgacgagcagtggcgctggctggccaacacgctgacagtggagctgctcaaggaact
gctgcccgaggcggccgatctgcaagtcacccgctacgcgttgcccaacctgcgggcgctgaacttcgtcatcgaggggattctcggtcagggcgtggcctatcaggcgcgattcgacccgcaagccaaagggctg
ggcgaatggctgcgcagccgccacctcgatatcccggagagcctcctatga

Alignment of *M. tuberculosis* Rv0981 and *M. marinum* orthologue of Rv0981

```
M. tuberculosis (H73Rv):         1 gtgtccgtgcgaattcttgtcgttgacgacgatcgtgcggtgcgcgagtcgctgcgccggt 61
                                    |||||||||| || |||||||| ||||||||||| ||||||||||||||||||||| |||
M. marinum (ATCC BAA-535):  5558332 atgtccgtgcggatacttgtcgtcgacgacgatcgcgcggtgcgcgagtcgctgcgcaggt 5558272


M. tuberculosis:                62 cgctttccttcaatggctattcggtcgaactggcccacgacggggttgaggcgctcgaca 121
                                   |||||||||||||||||||  |||| || ||||| ||||| || || || || ||||| |
M. marinum:                5558271 cgctttccttcaatggctacacggttgagctggcacacgatggtgtggaagctctcgaga 5558212


M. tuberculosis:               122 tgattgccagcgatcgccccgacgcgttggtcctggatgtcatgatgccgcggctggacg 181
                                   |||||||||| ||||| || ||||||||||| ||||| |||||||||||||| ||||| |
M. marinum:                5558211 tgattgccagtgatcggcctgacgcgttggtgctggacgtcatgatgccgcgactggatg 5558152


M. tuberculosis:               182 gcctcgaggtgtgccgtcagctccgcggcaccggcgacgacctgccgattctggtgctga 241
                                   || | ||||| |||||||| || ||| | ||||| || || ||||| ||||| |||||||
M. marinum:                5558151 gcttggaggtatgccgtcaacttcgcagtaccggtgatgatctgcccattctcgtgctga 5558092


M. tuberculosis:               242 ccgcgcgcgactcggtgtccgagcgggtggccgggctggacgccggtgccgacgactacc 301
                                   |||| || || || || ||||| ||||| || ||||| || ||||| || || |||||||
M. marinum:                5558091 ccgcccgtgattccgtctccgaacgggtcgcggggctcgatgccggcgcggatgactacc 5558032


M. tuberculosis:               302 taccaaagccgttcgccctcgaagagctgctggcacggatgcgggcgctgctgcgccgca 361
                                   | || ||||||||||| || ||||||||| |||| |||||||| ||| ||||||||||||
M. marinum:                5558031 tgccgaagccgttcgcgctggaagagctgttggcgcggatgcgcgcgttgctgcgccgca 5557972


M. tuberculosis:               362 ccaagcc------cgaggatgccgccgagtcgatggccatgaggttctccgacctgacgc 415
                                   |||||||      ||| || |||||||| ||| | ||||||| ||||||||||||||  |
M. marinum:                5557971 ccaagcccgaagacgacgaagccgccgattcggtagccatgacgttctccgacctgagcc 5557912


M. tuberculosis:               416 tggacccggtaacccgcgaagtcaaccgtggacagcgccggatcagcctgacccgcaccg 475
                                   |||||||||| ||||| || || | ||| ||   ||| || |||||||| ||||| ||||
M. marinum:                5557911 tggacccggtgacccgggaggtgacccgcggggcgcggcgcatcagcctcacccgaaccg 5557852


M. tuberculosis:               476 aatttgcattgctggagatgctgatcgccaatccgcggcgagtgctgacgcgcagccgta 535
                                   | || ||  |||||||||||||||||||||| ||||| || |||||||| |||||||| |
M. marinum:                5557851 agttcgccctgctggagatgctgatcgccaacccgcgacgggtgctgacccgcagccgca 5557792


M. tuberculosis:               536 tcctggaagaggtatggggattcgactttcccacctcgggcaacgcgctggaagtctacg 595
                                   |||| |||||||| ||||| |||||||||||||| || ||||||||||| || |||||||
M. marinum:                5557791 tcctcgaagaggtgtggggcttcgactttcccacgtccggcaacgcgctcgaggtctacg 5557732
```

```
M. tuberculosis:          596 tcgggtatctacgccgcaagaccgaggccgacggcgagccgcggctgatccacactgtgc 655
                              | || || || || || || ||||||||||| ||||||||||||||||||||| ||||
M. marinum:           5557731 tgggctacctgcggcgtaaaaccgaggccgagggcgagccgcggctgatccacaccgtgc 5557672

M. tuberculosis:          656 gcggagtgggttacgtgctacgtgaaacaccaccctga 693
                              |||| ||||||||||||||||| ||||| |||||||||
M. marinum:           5557671 gcggggtgggttacgtgctacgcgaaacgccaccctga 5557634
```

Intergenic region between *M. marinum* orthologue of Rv0981 and *M. marinum* orthologue of rpmF (266bp)

```
tagtttcgtcctaacgcctgagagcggcctgagaggcgcgccgaaatgacgacagcgtcacaatttgatcgggatcaataaccaacggtttgccagcgccgccgggcagccggcatcgatg
cacggcacggccgtcatgccgtgccgtatcacaaccgccacattggccccaccgggaaggggccgggttgcgccctctcagcagcgacggcagtcgccgctaagaggacagagacacccag
gcgcgatggcccagggtaatcggg
```

Alignment of Intergenic region between *M. marinum* orthologue of Rv0981 and *M. marinum* orthologue of rpmF (266bp) with Intergenic region between *M. tuberculosis* Rv0981 and Rv0980c (366 bp). Bases highlighted in green represent iSNP bases identified in the intergenic region between Rv0980c and Rv0981 in *M. tuberculosis* by Gutacker et al. 2006. No homologous region covering these bases were identified in *M. marinum*.

```
                    43 bp homologous region

M.tub          cagtgtcgtcgcagcgcctgagactggcctgagaggcgcgccgtggtatgcgggtttgt 59
                ||| |||||  | |||||||||  |||||||||||||||||   |   ||     ||
mari           tagtttcgtcctaacgcctgagagcggcctgagaggcgcgccgaaatg-acgacagcgt 58

m.tub          t-cgacgaggtttagccacatcagccaca--ttggccccatggacgggttttgtttggtg 116
                 | |   | |   |    || | | ||   ||| |  |   | ||||
mari           cacaatttgatcgggatcaataaccaacggtttgccagcgccgccgggcagccggcatcg 118

m.tub          gtcaagagtagggcgatttgtgctgcttggcggaagagttctcgtccgcgcgggtttcgc 176
                |  |  | | |||  |   ||| |  || || |     || |  |||| |   ||   |
mari           atgcacggcacggccgtc-atgccg--tgccgta-----tcacaaccgc-cacattggcc 169

m.tub          ctgctgacacacgatgagggcagaaccccaggtgcagtgtgcaccacgttccaccaatgt 236
               |  | |  | | |    |||  |  |||     |||| |  |  ||    || | ||
mari           ccaccggga-aggggccgggttgcgccctctcagcagcg-acggcagtcgccgctaa--- 224
```

```
m.tub           tggtgcggtttgtgcctgcgcccaggcgtggaacggcagggatcgtccgaataaacggtt 296
                    | ||   | |    | |||||||| | |  | |  || |  || |
mari            ----gaggacagag---acacccaggcgcg-atggcccagggtaatcggg---------- 266

m.tub           acccaccgcagggtaacaggcggtgggccgcccgcatctcaacatcgtcgcggtcagga 356

mari            ------------------------------------------------------------

m.tub           ggatttcga 365

mari            ---------
```

Alignment of *M. tuberculosis* rpmF and *M. marinum* orthologue of rpmF (Rv0979A)

```
M. tuberculosis (H37Rv):         1 atggctgtgccc
                                   |||||    ||
M. marinum (ATCC RAA-535): 5558772 atggccacacca


M. tuberculosis:                13 aagcgcagaaagtcgcgctcgaatacccgaagccggcgctcgcagtggaaggccgccaag 72
                                   |||||||| | ||||||| ||||||| ||||||||||||||||||||||||||| |||||
M. marinum:                5558760 aagcgcaggatgtcgcgcgcgaatactcgaagccggcgctcgcagtggaaggccaccaag 5558701


M. tuberculosis:                73 accgagctggtcggtgtgaccgtcgccggtcacgcccacaaggtgcctcggcgcttgctc 132
                                   |||| || ||||| || ||||| ||||||||    |||||||||||||| ||  |||||
M. marinum:                5558700 gccgaactcgtcggcgtcaccgttgccggtcagaagcacaaggtgcctcgccggctgctc 5558641


M. tuberculosis:               133 aaggccgcccggctcggcctcatcgatttcgataagcgctga 174
                                   ||||| ||||||||||| ||||||||| ||||    || |
M. marinum:                5558640 aaggcggcccggctcggtctcatcgatctcgaccgtcgatag 5558599
```

Intergenic region between *M. marinum* orthologue of rpmF and *M. marinum* orthologue of Rv0976c (295bp)

ggtcggacctaactcctcggtcggggtttcgggttcaacatgtcgcacggcatggccaatcggcacagcggcgacgaatgacgtaaacgatagtcggtgacctgctggttgccaaaaccgg

gcaccgccggatccgccgtgttcacgtaccgagtgaggcgggtatcacaccgtggcaggcgtcccgctatgctgacctaccggttggttggttcacccaccggcaagccctgcccaccggt

aaccccgatgaaggaggacgcggatggcgtctcgttctgatgcgtccggcgcc

Alignment of Intergenic region between *M. marinum* orthologue of rpmF and *M. marinum* orthologue of Rv0976c (295 bp) with Intergenic region between *M. tuberculosis* Rv0976c and Rv0977 (199 bp).

```
M.tub           -----------------------------------------------tggcgctgacctc 13
                                                                |||  || ||
mari            ggtcggacctaactcctcggtcggggtttcgggttcaacatgtcgcacggca-tggccaa 59

m.tub           ctggggc--cggcaaagcacccggcgtcccgtagc--gaaacggactctactccaatgga 69
                  ||  |  |||| | | |    |    |  |||   |  ||   ||     ||||
mari            tcggcacagcggcgacgaatgacgtaaacgatagtcggtgacctgctggttgccaaaacc 119

m.tub           cggcgcagaccgggccgcttcg-------actcag-gaagcgatctccgtcgcgaaacag 121
                 ||| | | | |  ||||   |       ||  || || |||     |    ||   |||
mari            gggcaccgccggatccgccgtgttcacgtaccgagtgaggcgggtatcacaccgtggcag 179

m.tub           g------gcagtgctaacctaccggttggttggt-------------agcgtcgcc---- 158
                |      ||  |||| ||||||||||||||||||             |||   |||
mari            gcgtcccgctatgctgacctaccggttggttggttcacccaccggcaagccctgcccacc 239

m.tub           ---gatcccaa---gggaggaatcgcatggc-cctgactgtggtgccg-------- 199
                    | ||| |    |||||| || |||||  ||   | || |||
mari            ggtaaccccgatgaaggaggacgcggatggcgtctcgttctgatgcgtccggcgcc 295
```

27 bp homologous region

Alignment of *M. tuberculosis* Rv0976c and *M. marinum* orthologue of Rv0976c

```
M. tuberculosis (H37Rv):        1 gtgcgtatcgga 12
                                  || || ||||
M. marinum (ATCC BAA-535): 5559068 gtacgcatcgcg 5559079


M. tuberculosis:               13 aactgctcgggcttctacggcgaccgcctctccgccatgcgcgagatgctgaccggcggc 72
                                  ||||| || ||||||||||| ||||| ||||| || |||||||| ||||| |||||||||
M. marinum:               5559080 aactgttccggcttctacggggaccgtctctcggcgatgcgcgaaatgctcaccggcggc 5559139


M. tuberculosis:               73 gagctggactatctcaccggcgactacctggccgagctgaccatgttgattctcggtcgc 132
                                  ||| ||||||| ||||||||||||||||| ||||| ||||||||| |||| || || |||
M. marinum:               5559140 gaggtggactacctcaccggcgactacctcgccgaactgaccatgctgatcctgggccgc 5559199


M. tuberculosis:              133 gaccgcatgaaaaaccccgatcgcggctacgcgaagaccttcctggcccagctcgaggac 192
                                  ||||| |||||||||||||| ||||||||||| ||||||||||||||||||||||| |||
M. marinum:               5559200 gaccggatgaaaaaccccgagcgcggctacgccaagaccttcctggcccagctcgaagac 5559259
```

```
M. tuberculosis:          193 tgcctgggcctggcccatgaccgcggggtgcgcatcgtcaccaacgccggtggcctgaac 252
                              |||||||| |||||||| || ||||| |||||||||||| |||||||||| ||||| ||
M. marinum:           5559260 tgcctgggactggcccacgagcgcggagtgcgcatcgtcgccaacgccggcggcctcaat 5559319

M. tuberculosis:          253 cccgccgggctggccaacgcggtgcgggcgctggccgcgcgcctgggcattccggcgcag 312
                              ||||||||||||||| | ||| |  |||| ||  ||| |||| |||||||  ||||||
M. marinum:           5559320 cccgccgggctggccgatgcgatcagggccctaaccgagcgcttgggcat--cggcgccc 5559377

M. tuberculosis:          313 g--tcgcccacgtggaaggcgacgacctgcaaccacgggcggccgaactggggctgggga 370
                              |  |||||||||||||||||||||| |||   || || ||||||||||| || ||||| |
M. marinum:           5559378 gcatcgcccacgtggaaggcgacgatctggcgccccgcgcggccgaactcggcctgggca 5559437

M. tuberculosis:          371 cgccactgacggccaacgcctacctgggcgcatggggcatcgtcgactgcttcgagcgcg 430
                              | || ||||| |||||||||||||||||||| |||||||||||||| ||| || |  |||
M. marinum:           5559438 caccgctgacagccaacgcctacctgggcgcgtggggcatcgtcgattgcctcaacagcg 5559497

M. tuberculosis:          431 gcgccgacgtcgtggtcaccggccgggtcaccgacgcctcggtggtcgtcggggcggcgg 490
                              | |||||||||||||||||||||||||||||||||||||||||| |||||||  | ||||
M. marinum:           5559498 gggccgacgtcgtggtcaccggccgggtcaccgacgcctcggtgatcgtcggatccgcgg 5559557

M. tuberculosis:          491 ccgcgcacttcggctggggccgcaccgactaccaccgactggccggcgccgtggtggccg 550
                              | |||||||||||||||||||||||||||||| |||  ||||||||||||||||||||||
M. marinum:           5559558 cggcgcacttcggctggggccgcaccgactacaaccagctggccggcgccgtggtggccg 5559617

M. tuberculosis:          551 ggcacgtgatcgaatgcggagtccaggccaccggcggcaactacgcgttcttcaccgaga 610
                              | || || ||||||||||| |||||||||||||||||||||||||| ||||||||||||
M. marinum:           5559618 gccatgtcatcgaatgcggggtccaggccaccggcggcaactacgccttcttcaccgagg 5559677

M. tuberculosis:          611 taggcgacctcactcacgccggcttcccgctggccgagatcgctgccgacggctcgtcgg 670
                              |   ||| || ||  ||||||||||||||||||||||| ||   ||||||||| | ||||
M. marinum:           5559678 tccccgatctgacctacgccggcttcccgctggccgaggtcagcgccgacggcacctcgg 5559737

M. tuberculosis:          671 tgatcaccaagcaccacggcaccggcggcctggtcagcgtcgacaccatcaccgcgcagc 730
                              |||||||||| |||| ||||||||| || |||||||||  ||||||| ||||||| || |
M. marinum:           5559738 tgatcaccaaacaccccggcaccggtgggctggtcagccccgacaccgtcaccgcacaac 5559797

M. tuberculosis:          731 tgctctacgagatcacgggcgctcggtatgccaacccggatgtgacggcccggatggaca 790
                              |||||||||||||  | ||||| || || ||||| ||||| |||||||||||||||||||
M. marinum:           5559798 tgctctacgagatatccggcgcccgctacgccaatccggacgtgacggcccggatggaca 5559857
```

```
M. tuberculosis:         791 gcgtcgaactgtcgcccgacgggccggatcgagtgcgcattagcggcgtgatcggcgaac 850
                              | || |||||||   ||| || |||||||| |||||||| ||||||||||||||||||
M. marinum:          5559858 ccatccaactgtccagcgatggcccggatcgggtgcgcatcagcggcgtgatcggcgaag 5559917

M. tuberculosis:         851 cgccgccgccgacctacaaggtgtcgctgaacagcatcggcggcttccgcaacgccatga 910
                             | || || || |||   |||||||||||||||||||||||||| ||||||||||||||||
M. marinum:          5559918 cccccccacccaccctgaaggtgtcgctgaacagcatcggcgggttccgcaacgccatga 5559977

M. tuberculosis:         911 cattcgtgctgaccggcctggacatcgatgccaaggccgacttggtgcgacgccagctcg 970
                             | |||||  |||| || ||| ||||||| |||||||||||  | |||| |||||||||||
M. marinum:          5559978 cgttcgtcttgacgggtctgaacatcgaggccaaggccgagctcgtgcaacgccagctcg 5560037

M. tuberculosis:         971 aggccgcgctgaccgtcaaacccgctgagctgcaatggacgctggcccgcaccgaccacc 1030
                             ||||| | ||||||||||||||||| || ||| ||||||| ||||||||||||||||||
M. marinum:          5560038 aggccaccctgaccgtcaaacccgccgaactggaatggaccctggcccgcaccgaccacg 5560097

M. tuberculosis:        1031 ccgacgccgacaccgaagaaaccgccagcgcgctgctcacctgcgtcgcccgcgatcccg 1090
                              ||||||||||||||| ||| |||||||||| |||||   |||||| | ||| || ||||
M. marinum:          5560098 tcgacgccgacaccgaggaagccgccagcgccctgctgcactgcgtggtccgtgaccccg 5560157

M. tuberculosis:        1091 accctgccaatgtgggtcggcaattctcctcggccgcggtcgaattggcgctggccagct 1150
                             |||| ||||||||||| ||  | ||||||||||||||||| ||||||||| |||||||||
M. marinum:          5560158 accccgccaatgtgggccgcaagttctcctcggccgcggtggaattggcgttggccagct 5560217

M. tuberculosis:        1151 acccggggttcaccgccaccgccccgcccggtgacggccaggtgtatggcgtgttcaccc 1210
                             | || || |||||| | ||||| || || || ||||||||||||||||| ||||||   |
M. marinum:          5560218 atcccggattcacctcgaccgcacccccgggcgacggccaggtgtatggggtgttcgtgc 5560277

M. tuberculosis:        1211 ccggctacgtcgacgccggcaaggtggcgcacatcgccgtgcacgccgacggcacccgca 1270
                             |||||||||| ||||||     |||| | |||||||||||||||||||||||||| ||||
M. marinum:          5560278 ccggctacgtggacgccaagctggtgccacacatcgccgtgcacgccgacggcacgcgca 5560337

M. tuberculosis:        1271 ccgagattccctgcgccactgagacgttggagctggcgccggcacatcccccggcgctgc 1330
                             |||| || ||||||||||| |||||  |||| ||||   |||   ||||||||||  |||
M. marinum:          5560338 ccgatatcccctgcgccaccgagaccctggaactggaatcggtcgatcccccggccttgc 5560397

M. tuberculosis:        1331 ccgacccgctgccggctggcccgacccggcgcgtaccgctgggcctgatcgctggcgccc 1390
                             |||| ||||||||  | ||||||||||| || ||||||||||||||||| || |||||||
M. marinum:          5560398 ccgatccgctgcccaccggcccgacccgacgggtaccgctgggcctgattgccggcgccc 5560457
```

```
M. tuberculosis:              1391 gcagcggcgacaagggcggatcggccaacgtcggcgtgtgggtacgcaccgacgagcagt 1450
                                   ||||||| ||||||||||| ||||||||||||||  | ||||| ||||||||||||||||
M. marinum:                5560458 gcagcggggacaagggcgggtcggccaacgtcggactatgggtgcgcaccgacgagcagt 5560517

M. tuberculosis:              1451 ggcgctggctggcgcacacgctgaccgtcgagctactcaaggagctgctgccagaaacgg 1510
                                   |||||||||||||  |||||||||| || ||||| |||||||| |||||||| ||  |||
M. marinum:                5560518 ggcgctggctggccaacacgctgacagtggagctgctcaaggaactgctgcccgaggcgg 5560577

M. tuberculosis:              1511 caggcctggtcgtcacccgtcacgtgctgccgaatctgcgggcactgaacttcgtcatcg 1570
                                   | |  |||   ||||||||  ||| | |||| || |||||||| ||||||||||||||||
M. marinum:                5560578 ccgatctgcaagtcacccgctacgcgttgcccaacctgcgggcgctgaacttcgtcatcg 5560637

M. tuberculosis:              1571 aggcaatcctcggccagggcgtcgcctaccaggcccgcttcgacccgcaggccaagggac 1630
                                   |||  || ||||| |||||||| ||||| ||||| || ||||||||||| ||||| || |
M. marinum:                5560638 aggggattctcggtcagggcgtggcctatcaggcgcgattcgacccgcaagccaaagggc 5560697

M. tuberculosis:              1631 tgggtgagtggctgcgcagccgccacgtcgagatcccggagaccctgttgtga 1683
                                   |||| || |||||||||||||||||| |||| |||||||||| |||  | |||
M. marinum:                5560698 tgggcgaatggctgcgcagccgccacctcgatatcccggagagcctcctatga 5560750
```

Alignment of *M. marinum* region from the end of the *M. marinum* orthologue of Rv0981 (green) to the end of *M. marinum* orthologue of Rv0976c (light blue) with the orthologous region in *M. ulcerans*, showing high percentage of homology and a similar absence of the PE_PGRS16, -17 and -18 in *M. ulcerans.*

```
M. marinum (ATCC BAA-535): 1 tcagggtggcgtttcgcgtagcacgtaacccaccccgcgcacggtgtggatcagccgcgg 60
                             |||||||||||||||||||||||||||||||||||| |||||||||||||||||||||||
M. ulcerans (Agy99): 5204730 tcagggtggcgtttcgcgtagcacgtaacccacccctcgcacggtgtggatcagccgcgg 5204789

M. marinum:               61 ctcgccctcggcctcggttttacgccgcaggtagcccacgtagacctcgagcgcgttgcc 120
                             ||||||||||||||||||||||||||||||||||||||||||||||||||||||||||||
M. ulcerans:         5204790 ctcgccctcggcctcggttttacgccgcaggtagcccacgtagacctcgagcgcgttgcc 5204849

M. marinum:              121 ggacgtgggaaagtcgaagccccacacctcttcgaggatgcggctgcgggtcagcacccg 180
                             ||||||||||||||||||||||||||||||||||||||||||||||||||||||||||||
M. ulcerans:         5204850 ggacgtgggaaagtcgaagccccacacctcttcgaggatgcggctgcgggtcagcacccg 5204909
```

```
M. marinum:          181 tcgcgggttggcgatcagcatctccagcagggcgaactcggttcgggtgaggctgatgcg 240
                         ||||||||||||||||||||||||||||||||||||||||||||||||||||||||||||
M. ulcerans:     5204910 tcgcgggttggcgatcagcatctccagcagggcgaactcggttcgggtgaggctgatgcg 5204969

M. marinum:          241 ccgcgccccgcgggtcacctcccgggtcaccgggtccaggctcaggtcggagaacgtcat 300
                         ||||||||||||||||||||||||||||||||||||||||||||||||||||||||||||
M. ulcerans:     5204970 ccgcgccccgcgggtcacctcccgggtcaccgggtccaggctcaggtcggagaacgtcat 5205029

M. marinum:          301 ggctaccgaatcggcggcttcgtcgtcttcgggcttggtgcggcgcagcaacgcgcgcat 360
                         ||||||||||||||||||||||||||||||| ||||||||||||||||||||||||||||
M. ulcerans:     5205030 ggctaccgaatcggcggcttcgtcgtcttcgagcttggtgcggcgcagcaacgcgcgcat 5205089

M. marinum:          361 ccgcgccaacagctcttccagcgcgaacggcttcggcaggtagtcatccgcgccggcatc 420
                         ||||||||||||||||||||||||||||||||||||||||||||||||||||||||||||
M. ulcerans:     5205090 ccgcgccaacagctcttccagcgcgaacggcttcggcaggtagtcatccgcgccggcatc 5205149

M. marinum:          421 gagccccgcgacccgttcggagacggaatcacgggcggtcagcacgagaatgggcagatc 480
                         |||||||||||||||||||||||||||||| |||||||||||||||||||||||||||||
M. ulcerans:     5205150 gagccccgcgacccgttcggagacggaatcccgggcggtcagcacgagaatgggcagatc 5205209

M. marinum:          481 atcaccggtactgcgaagttgacggcatacctccaagccatccagtcgcggcatcatgac 540
                         ||||||||| ||||||||||||||||||||||||||||||||||||||||||||||||||
M. ulcerans:     5205210 atcaccggtgctgcgaagttgacggcatacctccaagccatccagtcgcggcatcatgac 5205269

M. marinum:          541 gtccagcaccaacgcgtcaggccgatcactggcaatcatctcgagagcttccacaccatc 600
                         ||||||||||||||||||||||||||||||||||||||||||||||||||||||||||||
M. ulcerans:     5205270 gtccagcaccaacgcgtcaggccgatcactggcaatcatctcgagagcttccacaccatc 5205329

M. marinum:          601 gtgtgccagctcaaccgtgtagccattgaaggaaagcgacctgcgcagcgactcgcgcac 660
                         ||||||||||||||||||||||||||||||||||||||||||||||||||||||||||||
M. ulcerans:     5205330 gtgtgccagctcaaccgtgtagccattgaaggaaagcgacctgcgcagcgactcgcgcac 5205389

M. marinum:          661 cgcgcgatcgtcgtcgacgacaagtatccgcacggacattagtttcgtcctaacgcctga 720
                         ||||||||||||||||||||||||||||||||||||||||||||||||||||||||||||
M. ulcerans:     5205390 cgcgcgatcgtcgtcgacgacaagtatccgcacggacattagtttcgtcctaacgcctga 5205449

M. marinum:          721 gagcggcctgagaggcgcgccgaaatgacgacagcgtcacaatttgatcgggatcaataa 780
                         ||||||||||||||||||||||||||||||||||||||||||||||||||||||||||||
M. ulcerans:     5205450 gagcggcctgagaggcgcgccgaaatgacgacagcgtcacaatttgatcgggatcaataa 5205509
```

```
M. marinum:          781 ccaacggtttgccagcgccgccgggcagccggcatcgatgcacggcacggccgtcatgcc 840
                         |||||||||||||||||||||| |||||||||||||||||||| || |||||||||||||
M. ulcerans:     5205510 ccaacggtttgccagcgccgccaggcagccggcatcgatgcaccgcgcggccgtcatgcc 5205569

M. marinum:          841 gtgccgtatcacaaccgccacattggccccaccgggaaggggccgggttgcgccctctca 900
                         | ||||||||||||||||||||||||||||  ||||||||||||||||||||| ||||
M. ulcerans:     5205570 gcgccgtatcacaaccgccacattggccccactaggaaggggccgggttgcgcccactca 5205629

M. marinum:          901 gcagcgacggcagtcgccgctaagaggacagagacacccaggcgcgatggcccagggtaa 960
                         ||||| ||||| ||||||||||||||||||||||||||||||||||||||||||||||||
M. ulcerans:     5205630 gcagcaacggctgtcgccgctaagaggacagagacacccaggcgcgatggcccagggtaa 5205689

M. marinum:          961 tcgggctatcgacggtcgagatcgatgagaccgagccgggccgccttgagcagccggcga 1020
                         ||||||||||||||||||||||||||||||||||||||||||||||||||||||||||||
M. ulcerans:     5205690 tcgggctatcgacggtcgagatcgatgagaccgagccgggccgccttgagcagccggcga 5205749

M. marinum:         1021 ggcaccttgtgcttctgaccggcaacggtgacgccgacgagttcggccttggtggccttc 1080
                         ||||||||||||||||||||| ||||||||||||||||||||||||||||||||||||||
M. ulcerans:     5205750 ggcaccttgtgcttctgaccgccaacggtgacgccgacgagttcggccttggtggccttc 5205809

M. marinum:         1081 cactgcgagcgccggcttcgagtattcgcgcgcgacatcctgcgctttggtgtggccatg 1140
                         ||||||||||||||||||||||||||||||||||||||||||||||||||||||||||||
M. ulcerans:     5205810 cactgcgagcgccggcttcgagtattcgcgcgcgacatcctgcgctttggtgtggccatg 5205869

M. marinum:         1141 gtcggacctaactcctcggtcggggtttcgggttcaacatgtcgcacggcatggccaatc 1200
                         ||||||||||||||||||||||||||||||||||||| ||||||||||||||||||||||
M. ulcerans:     5205870 gtcggacctaactcctcggtcggggtttcgggttcaatatgtcgcacggcatggccaatc 5205929

M. marinum:         1201 ggcacagcggcgacgaatgacgtaaacgatagtcggtgacctgctggttgccaaaaccgg 1260
                         ||||||||||||||||||||||| ||||||||| ||||||||||||||||||||||||||
M. ulcerans:     5205930 ggcacagcggcgacgaatgacgtcaacgatagtaggtgacctgctggttgccaaaaccgg 5205989

M. marinum:         1261 gcaccgccggatccgccgtgttcacgtaccgagtgaggcgggtatcacaccgtggcaggc 1320
                         ||||||||||||||||||||||| || |||||||||||||||||||||||||||||||| 
M. ulcerans:     5205990 gcaccgccggatccgccgtgttcgcgcaccgagtgaggcgggtatcacaccgtggcagga 5206049

M. marinum:         1321 gtcccgctatgctgacctaccggttggttggttcacccaccggcaagccctgcccaccgg 1380
                         |||||||||||||||||||||||||||||||||||| ||||||||| ||||||||||||||
M. ulcerans:     5206050 gtcccgctatgctgacctaccggttggttggttcacgcaccggcaaaccctgcccaccgg 5206109
```

```
M. marinum:         1381 taaccccgatgaaggaggacgcggatggcgtctcgttctgatgcgtccggcgccgtacgc 1440
                         ||||||||||||||||||||||||||||||||||||||||||||||||||||||||||||
M. ulcerans:     5206110 taaccccgatgaaggaggacgcggatggcgtctcgttctgatgcgtccggcgccgtacgc 5206169

M. marinum:         1441 atcgcgaactgttccggcttctacggggaccgtctctcggcgatgcgcgaaatgctcacc 1500
                         ||||||||||||||||| ||||||||||||||||||||||||||||||||||||||||||
M. ulcerans:     5206170 atcgcgaactgttccggtttctacggggaccgtctctcggcgatgcgcgaaatgctcacc 5206229

M. marinum:         1501 ggcggcgaggtggactacctcaccggcgactacctcgccgaactgaccatgctgatcctg 1560
                         ||||||||||||||||||||||||||||||||||| ||||||||||||||| ||||||||
M. ulcerans:     5206230 ggcggcgaggtggactacctcaccggcgactacctggccgaactgaccatgttgatcctg 5206289

M. marinum:         1561 ggccgcgaccggatgaaaaaccccgagcgcggctacgccaagaccttcctggcccagctc 1620
                         ||||||||||||||||| ||||||||||||||||||||||||||||||||||||||||||
M. ulcerans:     5206290 ggccgcgaccggatgaagaaccccgagcgcggctacgccaagaccttcctggcccagctc 5206349

M. marinum:         1621 gaagactgcctgggactggcccacgagcgcggagtgcgcatcgtcgccaacgccggcggc 1680
                         |||||||||||||||||||||||||| |||||||||||||||||||||||||||||||||
M. ulcerans:     5206350 gaagactgcctgggactggcccacgatcgcggagtgcgcatcgtcgccaacgccggcggc 5206409

M. marinum:         1681 ctcaatcccgccgggctggccgatgcgatcagggccctaaccgagcgcttgggcatcggc 1740
                         ||||||||||||||||||||||||||||||||||||||| ||||| || |||||||||||
M. ulcerans:     5206410 ctcaatcccgccgggctggccgatgcgatcagggccctagccgagtgcctgggcatcggc 5206469

M. marinum:         1741 gcccgcatcgcccacgtggaaggcgacgatctggcgccccgcgcggccgaactcggcctg 1800
                         ||||||||||||||||||||||||||||||||||||||||||||||||||||||||||||
M. ulcerans:     5206470 gcccgcatcgcccacgtggaaggcgacgatctggcgccccgcgcggccgaactcggcctg 5206529

M. marinum:         1801 ggcacaccgctgacagccaacgcctacctgggcgcgtggggcatcgtcgattgcctcaac 1860
                         |||||| |||||||||||||||||||||||||||||||||||||||||||||||||||||
M. ulcerans:     5206530 ggcacatcgctgacagccaacgcctacctgggcgcgtggggcatcgtcgattgcctcaac 5206589

M. marinum:         1861 agcggggccgacgtcgtggtcaccggccgggtcaccgacgcctcggtgatcgtcggatcc 1920
                         |||||||||||||||||||||||||| |||||||||||||||||||||||||||||||||
M. ulcerans:     5206590 agcggggccgacgtcgtggtcaccgggcgggtcaccgacgcctcggtgatcgtcggatcc 5206649

M. marinum:         1921 gcggcggcgcacttcggctggggccgcaccgactacaaccagctggccggcgccgtggtg 1980
                         ||||||||||||||||||||||||||||||||||||||||||||||||||||||||||||
M. ulcerans:     5206650 gcggcggcgcacttcggctggggccgcaccgactacaaccagctggccggcgccgtggtg 5206709
```

```
M. marinum:          1981 gccggccatgtcatcgaatgcggggtccaggccaccggcggcaactacgccttcttcacc 2040
                          ||||||||||||||||||||||||||||||||||||||||||||||||||||||||||||
M. ulcerans:      5206710 gccggccatgtcatcgaatgcggggtccaggccaccggcggcaactacgccttcttcacc 5206769

M. marinum:          2041 gaggtccccgatctgacctacgccggcttcccgctggccgaggtcagcgccgacggcacc 2100
                          ||||||||||||||||||||||||||||||||||||||||||||||||||||||||||||
M. ulcerans:      5206770 gaggtccccgatctgacctacgccggcttcccgctggccgaggtcagcgccgacggcacc 5206829

M. marinum:          2101 tcggtgatcaccaaacaccccggcaccggtgggctggtcagccccgacaccgtcaccgca 2160
                          |||||||||||||||||||||||||||||||||||||||||||| |||||||||||||||
M. ulcerans:      5206830 tcggtgatcaccaaacaccccggcaccggtgggctggtcagcccagacaccgtcaccgca 5206889

M. marinum:          2161 caactgctctacgagatatccggcgcccgctacgccaatccggacgtgacggcccggatg 2220
                          ||||||||||||||||||||||||||||||||||||||||||||||||||||||||||||
M. ulcerans:      5206890 caactgctctacgagatatccggcgcccgctacgccaatccggacgtgacggcccggatg 5206949

M. marinum:          2221 gacaccatccaactgtccagcgatggcccggatcgggtgcgcatcagcggcgtgatcggc 2280
                          ||||||||||| ||||||||||||||||||||||||||||||||| ||||||||||||||
M. ulcerans:      5206950 gacaccatccagctgtccagcgatggcccggatcgggtgcgcatcggcggcgtgatcggc 5207009

M. marinum:          2281 gaagnnnnnnnacccaccctgaaggtgtcgctgaacagcatcggcgggttccgcaacgcc 2340
                          ||||       ||  |||||||||||||||||||||||||||||||||||||||||||||
M. ulcerans:      5207010 gaagcgcccccacagaccctgaaggtgtcgctgaacagcatcggcgggttccgcaacgcc 5207069

M. marinum:          2341 atgacgttcgtcttgacgggtctgaacatcgaggccaaggccgagctcgtgcaacgccag 2400
                          ||||||||||||||||||||||||||||||||||||||||||||||||||||||||||||
M. ulcerans:      5207070 atgacgttcgtcttgacgggtctgaacatcgaggccaaggccgagctcgtgcaacgccag 5207129

M. marinum:          2401 ctcgaggccaccctgaccgtcaaacccgccgaactggaatggaccctggcccgcaccgac 2460
                          ||||||||||||||||||||||||||||||||||||||||||||||||||||||||||||
M. ulcerans:      5207130 ctcgaggccaccctgaccgtcaaacccgccgaactggaatggaccctggcccgcaccgac 5207189

M. marinum:          2461 cacgtcgacgccgacaccgaggaagccgccagcgccctgctgcactgcgtggtccgtgac 2520
                          ||||||||||||||||||||||||||||||||||||||||||||||||||||||||||||
M. ulcerans:      5207190 cacgtcgacgccgacaccgaggaagccgccagcgccctgctgcactgcgtggtccgtgac 5207249

M. marinum:          2521 cccgaccccgccaatgtgggccgcaagttctcctcggccgcggtggaattggcgttggcc 2580
                          |||||||||||||||||||||||||| |||||||||||||||||||||||||||||||||
M. ulcerans:      5207250 cccgaccccgccaatgtgggccgcaaattctcctcggccgcggtggaattggcgttggcc 5207309
```

```
M. marinum:      2581 agctatcccggattcacctcgaccgcacccccgggcgacggccaggtgtatggggtgttc 2640
                      ||||| |||||||||||||||||||| |||||||||||||||||||||||||||||||||
M. ulcerans:  5207310 agctaccccggattcacctcgaccgcgcccccgggcgacggccaggtgtatggggtgttc 5207369

M. marinum:      2641 gtgcccggctacgtggacgccaagctggtgccacacatcgccgtgcacgccgacggcacg 2700
                      ||||||||||||||||||||||||||||||||||||||||||||||||||||||||||||
M. ulcerans:  5207370 gtgcccggctacgtggacgccaagctggtgccacacatcgccgtgcacgccgacggcacg 5207429

M. marinum:      2701 cgcaccgatatcccctgcgccaccgagaccctggaactggaatcggtcgatcccccggcc 2760
                      |||||| |||||||||||||||||||||||||||||||||||||||||||||||||||||
M. ulcerans:  5207430 cgcacctatatcccctgcgccaccgagaccctggaactggaatcggtcgatcccccggcc 5207489

M. marinum:      2761 ttgcccgatccgctgcccaccggcccgacccgacgggtaccgctgggcctgattgccggc 2820
                      ||||||||||||||||||||||||||||||||||||||||||||||||||||||||||||
M. ulcerans:  5207490 ttgcccgatccgctgcccaccggcccgacccgacgggtaccgctgggcctgattgccggc 5207549

M. marinum:      2821 gcccgcagcggggacaagggcgggtcggccaacgtcggactatgggtgcgcaccgacgag 2880
                      ||||||||||||||||||||||||||||||||||||||||||||||||||||||||||||
M. ulcerans:  5207550 gcccgcagcggggacaagggcgggtcggccaacgtcggactatgggtgcgcaccgacgag 5207609

M. marinum:      2881 cagtggcgctggctggccaacacgctgacagtggagctgctcaaggaactgctgcccgag 2940
                      |||||||||||||||||||||||| |||| ||||| ||||||||||||||||||||||||
M. ulcerans:  5207610 cagtggcgctggctggccaacacgttgaccgtggaactgctcaaggaactgctgcccgag 5207669

M. marinum:      2941 gcggccgatctgcaagtcacccgctacgcgttgcccaacctgcgggcgctgaacttcgtc 3000
                      |||||| |||||||||||||||||||||||||||||||||||||||||||||||||||||
M. ulcerans:  5207670 gcggccaatctgcaagtcacccgctacgcgttgcccaacctgcgggcgctgaacttcgtc 5207729

M. marinum:      3001 atcgaggggattctcggtcagggcgtggcctatcaggcgcgattcgacccgcaagccaaa 3060
                      |||||||||||||||||||| |||||||||||||||||||||||||||||||||||||||
M. ulcerans:  5207730 atcgaggggattctcggtcaaggcgtggcctatcaggcgcgattcgacccgcaagccaaa 5207789

M. marinum:      3061 gggctgggcgaatggctgcgcagccgccacctcgatatcccggagagcctcctatga 3117
                      |||||||||||||||||||||||||||||||||||||||||||||||||||||||||
M. ulcerans:  5207790 gggctgggcgaatggctgcgcagccgccacctcgatatcccggagagcctcctatga 5207846
```

Partial alignment of *M. marinum* region from the end of the *M. marinum* orthologue of Rv0981 (green) to the end of *M. marinum* orthologue of Rv0976c (light blue) with the orthologous region in *M. avium paratuberculosis*, showing high percentage of homology and a similar absence of the PE_PGRS16, -17 and -18 in *M. avium paratuberculosis.*

```
M. marinum (ATCC BAA-535):                  20 agcacgtaacccaccccgcgcacggtgtggatcagccgcggctcgccctcggcctcggtt 79
                                               ||||||||||||||||||||||| |||||||||| |||||||||||| ||||| ||||||
M. avium paratuberculosis (Map K-10): 2399073 agcacgtaacccaccccgcgcacagtgtggatcaaccgcggctcgccgtcggcttcggtt 2399014

M. marinum:                                 80 ttacgccgcaggtagcccacgtagacctcgagcgcgttgccggacgtgggaaagtcgaag 139
                                               || || ||||||||||| ||||| ||||| ||||| ||||| || ||||| |||||||||
M. avium paratuberculosis:            2399013 ttgcggcgcaggtagccgacgtacacctccagcgcattgcccgaggtggggaagtcgaag 2398954

M. marinum:                                140 ccccacacctcttcgaggatgcggctgcgggtcagcacccgtcgcgggttggcgatcagc 199
                                               ||||| ||||| |||||||||||||||||||| |||||||| ||||||||||||||||||
M. avium paratuberculosis:            2398953 ccccagacctcctcgaggatgcggctgcgggtgagcacccggcgcgggttggcgatcagc 2398894

M. marinum:                                200 atctccagcagggcgaactcggttcgggtgaggctgatgcgccgcgccccgcgggtcacc 259
                                               || |||||||| ||||||||||| || || |||||||| || |||  ||||||||| |||
M. avium paratuberculosis:            2398893 atttccagcagtgcgaactcggtgcgcgtcaggctgatccggcgctgcccgcgggtgacc 2398834

M. marinum:                                260 tcccgggtcaccgggtccaggctcaggtcggagaacgtcatggctaccgaatcggcggct 319
                                               ||||||||||||||||||||  |||| |||||||||||||| || || || ||||||
M. avium paratuberculosis:            2398833 tcccgggtcaccgggtccagcgtcagatcggagaacgtcatcgccacggattcggcg--- 2398777

M. marinum:                                320 tcgtcgtcttcgggcttggtgcggcgcagcaacgcgcgcatccgcgccaacagctcttcc 379
                                                  ||||| |||||||||||||||||||||| ||| |||||||| |||| ||| ||||||
M. avium paratuberculosis:            2398776 ---tcgtcctcgggcttggtgcggcgcagcagcgcccgcatccgggccagcagttcttcc 2398720

M. marinum:                                380 agcgcgaacggcttcggcaggtagtcatccgcgccggcatcgagccccgcgacccgttcg 439
                                               || ||||||||||| ||||||||||| || ||||| || || ||||| || ||||| |||
M. avium paratuberculosis:            2398719 agggcgaacggcttgggcaggtagtcgtcggcgcccgcgtccagcccggccacccgctcg 2398660

M. marinum:                                440 gagacggaatcacgggcggtcagcacgagaatgggcagatcatcaccggtactgcgaagt 499
                                               ||||| ||||| ||||| |||||||| || |||||||| || |||||||| ||||| ||
M. avium paratuberculosis:            2398659 gagaccgaatcgcgggccgtcagcaccaggatgggcaggtcgtcaccggtgctgcgcagc 2398600

M. marinum:                                500 tgacggcatacctccaagccatccagtcgcggcatcatgacgtccagcaccaacgcgtca 559
```

```
                                                    |||||||| ||||| | |||||||||||||||||||| || ||||| || | ||||||
M. avium paratuberculosis:              2398599 tgacggcacacctcgaggccatccagtcgcggcatcatcacatccaggacgagcgcgtcg 2398540


M. marinum:                                 560 ggccgatcactggcaatcatctcgagagcttccacaccatcgtgtgccagctcaaccgtg 619
                                                || ||||| || || ||||| |||||||| || || || || || |||| ||| || | |
M. avium paratuberculosis:              2398539 gggcgatcgctcgcgatcatgtcgagagcctcgaccccgtcatgggccaactcgacggag 2398480


M. marinum:                                 620 tagccattgaaggaaagcgacctgcgcagcgactcgcgcaccgcgcgatcgtcgtcgacg 679
                                                ||||| |||||||| | ||||||||| | |||||||||||||||||||||||||||||||
M. avium paratuberculosis:              2398479 tagccgttgaaggacaacgacctgcgtaacgactcgcgcaccgcgcgatcgtcgtcgacg 2398420


M. marinum:                                 680 acaagtatccgcacggacattagtttcgtcctaacgcctgagagcggcctgagaggcgcg 739
                                                || ||||| ||||||| |  ||||||||||| | |||||||||  | |||||||||||||
M. avium paratuberculosis:              2398419 accagtattcgcacggcccctagtttcgtcccaccgcctgagactgacctgagaggcgcg 2398360


M. marinum: 740     ccg 742
                    |||
M. avium paratuberculosis: 2398359 ccg 2398357



Score =  168 bits (85), Expect = 3e-041
 Identities = 166/193 (86%)
 Strand = Plus / Minus


M. marinum (ATCC BAA-535):                  973 cggtcgagatcgatgagaccgagccgggccgccttgagcagccggcgaggcaccttgtgc 1032
                                                |||||||||||||| || ||||| ||||||||||||||||||| ||| ||||||||||||
M. avium paratuberculosis (Map K-10): 2398283 cggtcgagatcgatcaggccgagacgggccgccttgagcagcctgcgcggcaccttgtgc 2398224


M. marinum:                                1033 ttctgaccggcaacggtgacgccgacgagttcggccttggtggccttccactgcgagcgc 1092
                                                |||||| || || || || |||||||| |||| |||    ||||||||||||| ||||
M. avium paratuberculosis:              2398223 cgctgacctgccaccgtcacaccgacgagctcggtcttctccgccttccactgcgcgcgc 2398164


M. marinum:                                1093 cggcttcgagtattcgcgcgacatcctgcgctttggtgtggccatggtcggacctaac 1152
                                                ||||| || || ||||||||||||||| ||||||||| |||  ||||||||||||| ||||||
M. avium paratuberculosis:              2398163 cggctacgggtgttcgcgcgacattctgcgcttgggtacggccatggtcgggcctaac 2398104


M. marinum:                                1153 tcctcggtcgggg 1165
                                                |||||||||||||
M. avium paratuberculosis:              2398103 tcctcggtcgggg 2398091
```

```
Score =  311 bits (157), Expect = 3e-084
 Identities = 244/273 (89%)
 Strand = Plus / Minus


M. marinum (ATCC BAA-535):                1438 cgcatcgcgaactgttccggcttctacggggaccgtctctcggcgatgcgcgaaatgctc 1497
                                               |||||||||||||| ||||| |||||||| ||||| || ||||| |||||||| |||||
M. avium paratuberculosis (Map K-10): 2397844 cgcatcgcgaactgctccgggttctacggcgaccggctgtcggccatgcgcgagatgctg 2397785


M. marinum:                               1498 accggcggcgaggtggactacctcaccggcgactacctcgccgaactgaccatgctgatc 1557
                                               ||||||||| ||||||||||||||||||||||||||||| ||||||||||||||||||||
M. avium paratuberculosis:            2397784 accggcggcaaggtggactacctcaccggcgactacctggccgaactgaccatgctgatc 2397725


M. marinum:                               1558 ctgggccgcgaccggatgaaaaaccccgagcgcggctacgccaagaccttcctggcccag 1617
                                               ||||||||||| ||||||||  |||||||||||||||||||||||||||||||| ||||
M. avium paratuberculosis:            2397724 ctgggccgcgatcggatgaagcaccccgagcgcggctacgccaagaccttcctgacccaa 2397665


M. marinum:                               1618 ctcgaagactgcctgggactggcccacgagcgcggagtgcgcatcgtcgccaacgccggc 1677
                                               ||||| |||||||||||   ||||| ||| ||||| || |||||||| ||||||||||||
M. avium paratuberculosis:            2397664 ctcgaggactgcctgggcgaggcccgcgaccgcggggtccgcatcgtggccaacgccggc 2397605


M. marinum:                               1678 ggcctcaatcccgccgggctggccgatgcgatc 1710
                                               || ||||| || ||||| |||||||| ||||||
M. avium paratuberculosis:            2397604 ggactcaacccggccggcctggccgacgcgatc 2397572


Score = 880 bits (444), Expect = 0.0

 Identities = 1112/1332 (83%), Gaps = 2/1332 (0%)
 Strand = Plus / Minus


M. marinum (ATCC BAA-535):                1780 cgcgcggccgaactcggcctgggcacaccgctgacagccaacgcctacctgggcgcgtgg 1839
                                               ||||| ||||| || || |||||||  |||||||| |||||||||||||||||||| |||
M. avium paratuberculosis (Map K-10): 2397502 cgcgccgccgagctggggctgggcagcccgctgaccgccaacgcctacctgggcgcctgg 2397443


M. marinum:                               1840 ggcatcgtcgattgcctcaacagcggggccgacgtcgtggtcaccggccgggtcaccgac 1899
                                               ||||||||||| |||||   |  ||| ||||||||||| |||||||||||||||||||||
M. avium paratuberculosis:            2397442 ggcatcgtcgactgcctgggcgacggcgccgacgtcgtcgtcaccggccgggtcaccgac 2397383
```

```
M. marinum:                    1900 gcctcggtgatcgtcggatccgcggcggcgcacttcggctggggccgcaccgactacaac 1959
                                    |||||||||||||||||  | || |||||||||||||||||||  |||| ||||||| ||
M. avium paratuberculosis:  2397382 gcctcggtgatcgtcggggcggccgcggcgcacttcggctgggaacgcagcgactacgac 2397323

M. marinum:                    1960 cagctggccggcgccgtggtggccggccatgtcatcgaatgcggggtccaggccaccggc 2019
                                    | ||| ||||| || |||||||||||||| |||||||| |||||||| ||||||||||||
M. avium paratuberculosis:  2397322 cggctcgccggggcggtggtggccggccacgtcatcgagtgcggggtgcaggccaccggc 2397263

M. marinum:                    2020 ggcaactacgccttcttcaccgaggtccccgatctgacctacgccggcttcccgctggcc 2079
                                    ||||||||  |||||||||||||||| ||||| |||||| |||| || ||||||||||||
M. avium paratuberculosis:  2397262 ggcaactattccttcttcaccgaggtgcccgacctgacccacgcgggtttcccgctggcc 2397203

M. marinum:                    2080 gaggtcagcgccgacggcacctcggtgatcaccaaacaccccggcaccggtgggctggtc 2139
                                    |||||   |||||||||| |||||||||||||||| || || |||||||| |||||||||
M. avium paratuberculosis:  2397202 gaggtgcacgccgacggctcctcggtgatcaccaagcatccgggcaccggcgggctggtc 2397143

M. marinum:                    2140 agccccgacaccgtcaccgcacaactgctctacgagatatccggcgcccgctacgccaat 2199
                                    |||  ||||||||||||||| |||||||| ||||||||  ||||||| || ||||||||
M. avium paratuberculosis:  2397142 agcgtcgacaccgtcaccgcgcaactgctgtacgagatcaccggcgcgcgttacgccaac 2397083

M. marinum:                    2200 ccggacgtgacggcccggatggacaccatccaactgtccagcgatggcccggatcgggtg 2259
                                    || ||||| || ||||||||||||||| || | ||||||  ||| ||||| || ||||||
M. avium paratuberculosis:  2397082 cccgacgtcaccgcccggatggacaccgtcgagctgtcctccgacggccccgaccgggtg 2397023

M. marinum:                    2260 cgcatcagcggcgtgatcggcgaagcccccccacccaccctgaaggtgtcgctgaacagc 2319
                                    |||||||||||||||  ||| ||  | || || ||||||   ||||||||||||||||||
M. avium paratuberculosis:  2397022 cgcatcagcggcgtgcgcggtgagccgccgccgcccacctacaaggtgtcgctgaacagc 2396963

M. marinum:                    2320 atcggcgggttccgcaacgccatgacgttcgtcttgacgggtctgaacatcgaggccaag 2379
                                    |||||||| ||||||||| | ||||| |||||  |||| || ||| ||||||||||||||
M. avium paratuberculosis:  2396962 atcggcggattccgcaactcgatgaccttcgtgctgaccggcctggacatcgaggccaag 2396903

M. marinum:                    2380 gccgagctcgtgcaacgccagctcgaggccaccctgaccgtcaaacccgccgaactggaa 2439
                                    |||||||| ||||  || |||||  || || |  || | ||||||||||| || ||| |
M. avium paratuberculosis:  2396902 gccgagctggtgcgtcggcagctgcagtccgcgttggcggtcaaacccgcggagctgcag 2396843

M. marinum:                    2440 tggaccctggcccgcaccgaccacgtcgacgccgacaccgaggaagccgccagcgccctg 2499
                                    ||| | |||||||||||||||||  |||||||||||||||| || |||||||||||||||
M. avium paratuberculosis:  2396842 tggtcgctggcccgcaccgaccatctcgacgccgacaccgaagaggccgccagcgccctg 2396783
```

```
M. marinum:                          2500 ctgcactgcgtggtccgtgaccccgaccccgccaatgtgggccgcaagttctcctcggcc 2559
                                          ||||||||||| ||||| || |||||||| |||||||| || ||| | ||||||||||||
M. avium paratuberculosis:        2396782 ctgcactgcgtcgtccgcgatcccgacccggccaatgtcgggcgccaattctcctcggcc 2396723

M. marinum:                          2560 gcggtggaattggcgttggccagctatcccgga-ttcacctcgaccgcacccccgggcga 2618
                                          || || ||||||||  | ||||||||||| ||| ||||| || ||||| || |||| |||
M. avium paratuberculosis:        2396722 gcagtcgaattggcactcgccagctatccgggatttcacgtc-accgccccgccggccga 2396664

M. marinum:                          2619 cggccaggtgtatggggtgttcgtgcccggctacgtggacgccaagctggtgccacacat 2678
                                          |||||||||||| || ||||||    ||||||| || ||||||| |   ||||||| |||||
M. avium paratuberculosis:        2396663 cggccaggtgtacggcgtgttcaccgccggctatgtcgacgccgataaggtgccgcacat 2396604

M. marinum:                          2679 cgccgtgcacgccgacggcacgcgcaccgatatcccctgcgccaccgagaccctggaact 2738
                                          ||| ||||||||||||||| | ||   ||| || || ||||||||||| ||  |||| ||
M. avium paratuberculosis:        2396603 cgcggtgcacgccgacggcgcccgggtcgacattccttgcgccaccgatactttggagct 2396544

M. marinum:                          2739 ggaatcggtcgatcccccggccttgcccgatccgctgcccaccggcccgacccgacgggt 2798
                                          ||   ||||||||  ||||||  ||||||| ||||||||   |||||||   || || |
M. avium paratuberculosis:        2396543 ggcgccggtcgatgacccggcgctgcccgaaccgctgccggacggcccggtgcgccgcgc 2396484

M. marinum:                          2799 accgctgggcctgattgccggcgcccgcagcggggacaagggcgggtcggccaacgtcgg 2858
                                          ||||||||    || ||||| |||| |||||| ||||||||||| ||||||||||||||
M. avium paratuberculosis:        2396483 gccgctgggtactatcgccggggcccccagcggagacaagggcggctcggccaacgtcgg 2396424

M. marinum:                          2859 actatgggtgcgcaccgacgagcagtggcgctggctggccaacacgctgacagtggagct 2918
                                            | ||||| |||||||||||||||||||||||||||||| ||||| |||| || ||||
M. avium paratuberculosis:        2396423 ggtctgggtccgcaccgacgagcagtggcgctggctggcccacacgttgaccgtcgagcg 2396364

M. marinum:                          2919 gctcaaggaactgctgcccgaggcggccgatctgcaagtcacccgctacgcgttgcccaa 2978
                                          |||||  || |||||||||||||| |||||  |    ||||||||| ||  | |||||||
M. avium paratuberculosis:        2396363 gctcaccgagctgctgcccgaggccgccgagttcgccgtcacccgccacctgctgcccaa 2396304

M. marinum:                          2979 cctgcgggcgctgaacttcgtcatcgagggattctcggtcagggcgtggcctatcaggc 3038
                                          |||||| ||| | |||||||||||||| || || ||||| ||||| || |||||||| ||
M. avium paratuberculosis:        2396303 cctgcgcgcggtcaacttcgtcatcgacggcatcctcggacagggtgtcgcctatcaagc 2396244

M. marinum:                          3039 gcgattcgacccgcaagccaaagggctgggcgaatggctgcgcagccgccacctcgatat 3098
                                           || ||||||||||||||||| ||||||||||||||||||||| ||||| ||||||| ||
M. avium paratuberculosis:        2396243 ccggttcgacccgcaagccaaggggctgggcgaatggctgcgcggccgctacctcgacat 2396184
```

```
M. marinum:                             3099 cccggagagcct 3110
                                             ||||||||||||
M. avium paratuberculosis:           2396183 cccggagagcct 2396172
```
