# Supplementary material for: Insights into the evolutionary history of tubercle bacilli as disclosed by genetic rearrangements within a PE_PGRS duplicated gene pair
Source: BMC Evol Biol. 2006 Dec 12;6:107. doi: 10.1186/1471-2148-6-107 (PMC1762029; doi:10.1186/1471-2148-6-107)
Supplement: Additional file 4 — Characteristics of the whole collection of tubercle bacilli isolates used in this study. Lines in yellow indicate the 98 isolates, whose PE_PGRS17 and PE_PGRS18 sequences (nucleotide positions + 31 to +712) were subjected to sequencing. *According the Brudey et al. [56] §According to Gutierrez et al. [19] [file 1471-2148-6-107-S4.pdf]

Isolates	Origin	ST*	Genotype*	PGG	12/40 in PE_PGRS17	12/40 in PE_PGRS18	TbD1	Host	Spoligotype
M. tuberculosis 974	SOUTH AFRICA	orphan	CAS	1	+	-	-	Human	■■■□□□□■□■■■■■■■■■□ ■■■□□□□□□□□□□□□■■■■■■■■■
M. tuberculosis 953	SOUTH AFRICA	1	Beijing	1	+	-	-	Human	□□□□□□□□□□□□□□□□□□□ □□□□□□□□□□□□□□□■■■■■■■■■
M. tuberculosis 1945	SOUTH AFRICA	1	Beijing	1	+	-	-	Human	□□□□□□□□□□□□□□□□□□□ □□□□□□□□□□□□□□□■■■■■■■■■
M. tuberculosis 618	SOUTH AFRICA	1	Beijing	1	+	-	-	Human	□□□□□□□□□□□□□□□□□□□ □□□□□□□□□□□□□□□■■■■■■■■■
M. tuberculosis 2076	SOUTH AFRICA	1	Beijing	1	+	-	-	Human	□□□□□□□□□□□□□□□□□□□ □□□□□□□□□□□□□□□■■■■■■■■■
M. tuberculosis 1453	SOUTH AFRICA	1	Beijing	1	+	-	-	Human	□□□□□□□□□□□□□□□□□□□ □□□□□□□□□□□□□□□■■■■■■■■■
M. tuberculosis 1189	SOUTH AFRICA	1	Beijing	1	+	-	-	Human	□□□□□□□□□□□□□□□□□□□ □□□□□□□□□□□□□□□■■■■■■■■■
M. tuberculosis 1984	SOUTH AFRICA	1	Beijing	1	+	-	-	Human	□□□□□□□□□□□□□□□□□□□ □□□□□□□□□□□□□□□■■■■■■■■■
M. tuberculosis 2701	SOUTH AFRICA	1	Beijing	1	+	-	-	Human	□□□□□□□□□□□□□□□□□□□ □□□□□□□□□□□□□□□■■■■■■■■■
M. tuberculosis 407	SOUTH AFRICA	1	Beijing	1	+	-	-	Human	□□□□□□□□□□□□□□□□□□□ □□□□□□□□□□□□□□□■■■■■■■■■
M. tuberculosis 1659	SOUTH AFRICA	orphan	EAI5	1	+	-	+	Human	■■■■□■■■■■■■■■■■■■■ ■■■■■■■■■□□□□■□■■■■■■■■■
M. tuberculosis 20030423	Asia	517	EAI5	1	+	-	+	Human	■■■■■■■■■■■■■■■■■■■ ■■■■■■■■■□□□□■□■■■■■□□■■
M. tuberculosis 20021448	INDIA		EAI3_IND	1	+	-	+	Human	■□□■■■■■■■■■■■■■■■■ ■■■■■■■■■□□□□■□■■□□□■■■■
M. tuberculosis 20030173	INDIA		EAI3_IND	1	+	-	+	Human	■□□■■■■■■■■■■■■■■■□ □■■■■■■■■□□□□■□■■□□□■■■■
M. tuberculosis 20030271	MADAGASCAR	109	EAI8_MDG	1	+	-	+	Human	■□□■■■■■■■■■■■■■■■□ ■■■■■■■■■□□□□■□■■■■■■■■■
M. tuberculosis 20030400	INDIA	orphan	EAI	1	+	-	+	Human	■□□■■■■■■■■■■■■■■■■ ■■■■■■■□□□□□□□□□□□□□■■■■
M. tuberculosis 20030491	UNKNOWN		EAI6_BDG1	1	+	-	+	Human	■■■■■■■■■■■■■■■■■■■ ■■■□□■■■■□□□□■□■■■■■■■■■
M. tuberculosis 20030281	UNKNOWN		EAI3_IND	1	+	-	+	Human	■□□■■■■■■■■■■■■■■■■ ■■■■■■■■■□□□□■□■■□□□■■■■
M. tuberculosis 20031604	CONGO	orphan	EAI	1	+	-	+	Human	■■□□□□□■■■■■■■■■■■■ ■■■■■■■■■□□□□■□■■■■■■■■■
M. tuberculosis 20031033	UNKNOWN	orphan	EAI	1	+	-	+	Human	■■■■■■■■■■■■■■■■■■■ ■■■■■■■■■□□□□■□■□□□□□■■■
M. tuberculosis 20031571	INDIA		EAI3_IND	1	+	-	+	Human	■□□■■■■■■■■■■■■■■■■ ■■■■■■■■■□□□□■□■■□□□■■■■
M. tuberculosis 20031025	PHILIPPINES	orphan	EAI	1	+	-	+	Human	■■□■■■■■■■■■■■■■■■□ □■■■■■■■■□□□□■□■■■■■■■■■
M. tuberculosis 20031068	PHILIPPINES	orphan	EAI	1	+	-	+	Human	■■□■■■■■■■■■■■■■■■□ □■■■■■■■■□□□□■□■■■■■□■■■
M. tuberculosis 20031059	INDIA	orphan	EAI	1	+	-	+	Human	■■■■■■■■■■■■■■■■■□□ □□■■■■■■■□□□□■□■■■■■□■■■
*M. tuberculosis*19960357	CAMBODIA	1	Beijing	1	+	-	-	Human	□□□□□□□□□□□□□□□□□□□ □□□□□□□□□□□□□□□■■■■■■■■■
M. tuberculosis 19981121	REUNION	1	Beijing	1	+	-	-	Human	□□□□□□□□□□□□□□□□□□□ □□□□□□□□□□□□□□□■■■■■■■■■

M. tuberculosis 1	MADAGASCAR	21	CAS	1	+	-	-	Human	■■■□□□□■■□■■■■■■■■■ □□□□□□□□□□□□□□□□■■■■■■■■
M. tuberculosis 2	MADAGASCAR	21	CAS	1	+	-	-	Human	■■■□□□□■■□■■■■■■■■■ □□□□□□□□□□□□□□□□■■■■■■■■
M. tuberculosis 3	MADAGASCAR	21	CAS	1	+	-	-	Human	■■■□□□□■■□■■■■■■■■■ □□□□□□□□□□□□□□□□■■■■■■■■
M. tuberculosis 4	MADAGASCAR	21	CAS	1	+	-	-	Human	■■■□□□□■■□■■■■■■■■■ □□□□□□□□□□□□□□□□■■■■■■■■
M. tuberculosis 5	MADAGASCAR	21	CAS	1	+	-	-	Human	■■■□□□□■■□■■■■■■■■■ □□□□□□□□□□□□□□□□■■■■■■■■
M. tuberculosis 7	COMOROS	21	CAS	1	+	-	-	Human	■■■□□□□■■□■■■■■■■■■ □□□□□□□□□□□□□□□□■■■■■■■■
M. tuberculosis 10	FRENCH GUIANA	1762	EAI3-India	1	+	-	+	Human	■□□■■■■■■■■■■■■■■■■ ■□□■■■■■■□□□□■□■■□□□■■■■
M. tuberculosis 11	MARTINIQUE	1762	EAI3-India	1	+	-	+	Human	■□□■■■■■■■■■■■■■■■■ ■□□■■■■■■□□□□■□■■□□□■■■■
M. tuberculosis 12	FRENCH GUIANA	958	MANU1	1	+	-	-	Human	■■■■■■■■■■■■■■■■■■■ ■■■■■■■□□□□□□■□■■■■■□■■■
M. tuberculosis 13	FRENCH GUIANA	483	EAI	1	+	-	+	Human	■■□■■■■■■■■■■■■■■■■ □□■■■■■■■□□□□■□■■■■■□□□■
M. tuberculosis 14	FRENCH GUIANA	48	EAI1	1	+	-	+	Human	■■■■■■■■■■■■■■■■■■■ ■■■■■■■■■□□□□■□■■■■■□■■■
M. tuberculosis 15	FRENCH GUIANA	89	EAI2-Nonthaburi	1	+	-	+	Human	■■□■■■■□□□□□□□□□□□□ □□□□□□■■■□□□□■□■■■■■■■■■
M. tuberculosis 16	FRENCH GUIANA	48	EAI	1	+	-	+	Human	■■■■■■■■■■■■■■■■■■■ ■■■■■■■■■□□□□■□■■■■■□■■■
M. tuberculosis 18	FRENCH GUIANA	11	EAI3-India	1	+	-	+	Human	■□□■■■■■■■■■■■■■■■■ ■■■■■■■■■□□□□■□■■□□□■■■■
M. tuberculosis 19	FRENCH GUIANA	1340	EAI	1	+	-	+	Human	■■■■■■■■■■■■■■■■□■■ ■■■□■■■■■□□□□■□■■□■■■■■■
M. tuberculosis 21	FRENCH GUIANA	19	EAI2-Manilla	1	+	-	+	Human	■■□■■■■■■■■■■■■■■■■ □□■■■■■■■□□□□■□■■■■■■■■■
M. tuberculosis 23	FRENCH GUIANA	76	EAI	1	+	-	+	Human	■□□■■■■□□□□□□□□□□■□ ■■■■■■■■■□□□□■□■■■■■■■■■
M. tuberculosis 25	FRENCH GUIANA	924	EAI	1	+	-	+	Human	■■■■■■■■■■■□□□□□□□□ □□□□□■■■■□□□□■□■■□■■■■■■
M. tuberculosis 26	FRENCH GUIANA	2861	EAI	1	+	-	+	Human	■■■■■■■■■■■■■■□■□■■ ■■■□■■■■■□□□□■□■■□■■■■■■
M. tuberculosis 24	FRENCH GUIANA	1340	EAI	1	+	-	+	Human	■■■■■■■■■■■■■■■■□■■ ■■■□■■■■■□□□□■□■■□■■■■■■
M. tuberculosis 20	FRENCH GUIANA	19	EAI2-Manilla	1	+	-	+	Human	■■□■■■■■■■■■■■■■■■■ □□■■■■■■■□□□□■□■■■■■■■■■
M. tuberculosis 28	FRENCH GUIANA	1340	EAI	1	+	-	+	Human	■■■■■■■■■■■■■■■■□■■ ■■■□■■■■■□□□□■□■■□■■■■■■
M. tuberculosis 27	FRENCH GUIANA	11	EAI3-India	1	+	-	+	Human	■□□■■■■■■■■■■■■■■■■ ■■■■■■■■■□□□□■□■■□□□■■■■
M. tuberculosis 29	FRENCH GUIANA	89	EAI2-Nonthaburi	1	+	-	+	Human	■■□■■■■□□□□□□□□□□□□ □□□□□□■■■□□□□■□■■■■■■■■■
M. tuberculosis 30	FRENCH GUIANA	924	EAI	1	+	-	+	Human	■■■■■■■■■■■□□□□□□□□ □□□□□■■■■□□□□■□■■□■■■■■■
M. tuberculosis 31	FRENCH GUIANA	1340	EAI	1	+	-	+	Human	■■■■■■■■■■■■■■■■□■■ ■■■□■■■■■□□□□■□■■□■■■■■■
M. tuberculosis 32	FRENCH GUIANA	26	CAS	1	+	-	-	Human	■■■□□□□■■■■■■■■■■■■ ■■■□□□□□□□□□□□□■■■■■■■■■
M. tuberculosis 33	FRENCH GUIANA	2862	EAI	1	+	-	+	Human	■■■■■■■□□■■■■■■■■■■ ■■■■■■■■■□□□□■□■■■■■■■■■
M. tuberculosis 36	FRENCH GUIANA	11	EAI3-India	1	+	-	+	Human	■□□■■■■■■■■■■■■■■■■ ■■■■■■■■■□□□□■□■■□□□■■■■

M. tuberculosis 37	FRENCH GUIANA	349	EAI	1	+	-	+	Human	■■■■■■■■■■■■□■■■■■■ ■■■■■■■■■□□□□■□■■■■■□■■■
M. tuberculosis 45	FRENCH GUIANA	129	CAS	1	+	-	-	Human	■■■□□□□□□■■■■■■■■■■ ■■■□□■■■■□□□□■□■■■■■■■■■
M. tuberculosis 47	BARBADOS	2104	EAI3-India	1	+	-	+	Human	■□□□□□□□□□□□□□■■■■■ ■■■■■■■■■□□□□■□■■□□□■■■■
M. tuberculosis 40	FRENCH GUIANA	958	EAI	1	+	-	+	Human	■■■■■■■■■■■■■■■■■■■ ■■■■■■■□□□□□□■□■■■■■□■■■
M. tuberculosis 41	FRENCH GUIANA	152	EAI	1	+	-	+	Human	■■■■■■■■■■■■■■■■■■■ ■■■■■■■□■□□□□■□■■■■■■■■■
M. tuberculosis 42	FRENCH GUIANA	924	CAS	1	+	-	-	Human	■■■■■■■■■■■□□□□□□□□ □□□□□■■■■□□□□■□■■□■■■■■■
M. tuberculosis 44	FRENCH GUIANA	702	EAI	1	+	-	+	Human	■■■□□□□□□■■■■■■■□■■ ■■■□□■■■■□□□□■□■■■■■■■■■
M. tuberculosis 48	MARTINIQUE	2863	EAI	1	+	-	+	Human	■■□■□■■■■■■■■■■■■■■ ■■■■■■■■■□□□□■□■■□□□■■■■
M. tuberculosis 49	FRENCH GUIANA	129	EAI	1	+	-	-	Human	■■■□□□□□□■■■■■■■■■■ ■■■□□■■■■□□□□■□■■■■■■■■■
M. tuberculosis 51	FRENCH GUIANA	1062	EAI	1	+	-	+	Human	■■■□□□□□□■■■■■■■□□□ □□□□□■■■■□□□□■□■■■■■■■■■
M. tuberculosis CN116116	UNITED STATES	1149	U	1	+	-	-	Human	■■■■■■■■■■■■■■■■■■■ ■■■■■■■■■■■■■■■■■□■■□■■■
M. tuberculosis 548	BRAZIL	1	Beijing	1	+	-	-	Human	□□□□□□□□□□□□□□□□□□□ □□□□□□□□□□□□□□□■■■■■■■■■
M. tuberculosis BE 5867	UNITED STATES	139	EAI4-Vietnam	1	+	-	+	Human	■■■■■■■■■■■■■■■■■■■ ■■■■■■□□■□□□□■□■■■■■■■■■
M. tuberculosis BE 11677	UNITED STATES	11	EAI3-India	1	+	-	+	Human	■□□■■■■■■■■■■■■■■■■ ■■■■■■■■■□□□□■□■■□□□■■■■
M. tuberculosis IK 6464	UNITED STATES	8	EAI5	1	+	-	+	Human	■□□□□□□□□□□□□■■■■■■ ■■■■■■■■■□□□□■□■■■■■■■■■
M. tuberculosis MY5 13141	UNITED STATES	26	CAS1-Delhi	1	+	-	-	Human	■■■□□□□■■■■■■■■■■■■ ■■■□□□□□□□□□□□□■■■■■■■■■
M. tuberculosis GD30 14-30	INDIA	26	CAS1-Delhi	1	+	-	-	Human	■■■□□□□■■■■■■■■■■■■ ■■■□□□□□□□□□□□□■■■■■■■■■
M. tuberculosis 14392 BE3	KOREA. REPUBLIC OF	956	U	1	+	-	-	Human	■■■■■■■■■■■■■■■■■■■ ■■■■■■■■■■■■■□□□□□□□□□■■
M. tuberculosis HR36 12387	PHILIPPINES	892	EAI5	1	+	-	+	Human	■■■■■■■■■■■■■■■■■■■ □□■■■■■■■□□□□■□■■■■■■■■■
M. tuberculosis KY 10583	RUSSIA	269	Beijing Like	1	+	-	-	Human	□□□□□□□□□□□□□□□□□□□ □□□□□□□□□□□□□□□□□■■■■■■■
M. tuberculosis 35	COMOROS	26	EAI	1	+	-	+	Human	■■■□□□□■■■■■■■■■■■■ ■■■□□□□□□□□□□□□■■■■■■■■■
M. tuberculosis 50	FRENCH GUIANA	2864	EAI	1	+	-	+	Human	■■■□□□□□□■■■■■■■■■■ ■■■□□■■■■□□□□■□■■■■□■■■■
M. tuberculosis HE7 13454	ECUADOR	269	Beijing Like	1	+	-	-	Human	□□□□□□□□□□□□□□□□□□□ □□□□□□□□□□□□□□□□□■■■■■■■
M. tuberculosis HI 5116	UNITED STATES	246	U	1	+	-	-	Human	■■■■■■■■■■■■■■■■■■■ ■■■■■■■■■■■■■■■■■■■■□■■■
M. tuberculosis N2 1592	UNITED STATES	1	Beijing	1	+	-	-	Human	□□□□□□□□□□□□□□□□□□□ □□□□□□□□□□□□□□□■■■■■■■■■
M. tuberculosis AR2 12360	UNITED STATES	1	Beijing	1	+	-	-	Human	□□□□□□□□□□□□□□□□□□□ □□□□□□□□□□□□□□□■■■■■■■■■
M. tuberculosis H63514	UNITED STATES	190	Beijing Like	1	+	-	-	Human	□□□□□□□□□□□□□□□□□□□ □□□□□□□□□□□□□□□■■■■■□■■■
M. tuberculosis KF6 11091	UNITED STATES	19	EAI2-Manilla	1	+	-	+	Human	■■□■■■■■■■■■■■■■■■■ □□■■■■■■■□□□□■□■■■■■■■■■
M. tuberculosis HE4 11062	UNITED STATES	1	Beijing	1	+	-	-	Human	□□□□□□□□□□□□□□□□□□□ □□□□□□□□□□□□□□□■■■■■■■■■

M. tuberculosis BE 10299	UNITED STATES	orphan	orphan	1	+	-	+	Human	■□□■■■■■■■■■■■■■■■■ □□■■■■■■■□□□□■□■■□□□■■■■
M. tuberculosis 12433 001	UNITED STATES	1970	EAI6-BGD1	1	+	-	+	Human	■■■■■■■■■■■■■■■■■□■ ■■■□■■■■■□□□□■□■■■■■■■■■
M. tuberculosis H9 11671	UNITED STATES	1573	EAI5	1	+	-	+	Human	■■■■■■■■■■■■■■■■■■□ □■■■■■■■■□□□□■□■■□■■■■■■
M. tuberculosis C12 11646	UNITED STATES	1	Beijing	1	+	-	-	Human	□□□□□□□□□□□□□□□□□□□ □□□□□□□□□□□□□□□■■■■■■■■■
M. tuberculosis W187 13160	UNITED STATES	1	Beijing	1	+	-	-	Human	□□□□□□□□□□□□□□□□□□□ □□□□□□□□□□□□□□□■■■■■■■■■
M. tuberculosis IP 13089	UNITED STATES	19	EAI2-Manilla	1	+	-	+	Human	■■□■■■■■■■■■■■■■■■■ □□■■■■■■■□□□□■□■■■■■■■■■
M. tuberculosis HD16 12915	UNITED STATES	1	Beijing	1	+	-	-	Human	□□□□□□□□□□□□□□□□□□□ □□□□□□□□□□□□□□□■■■■■■■■■
M. tuberculosis 13444 001	UNITED STATES	1	Beijing	1	+	-	-	Human	□□□□□□□□□□□□□□□□□□□ □□□□□□□□□□□□□□□■■■■■■■■■
M. tuberculosis NP 13436	UNITED STATES	427	CAS1-Delhi	1	+	-	-	Human	■■■□□□□■■■■■□□□■■■■ ■■■□□□□□□□□□□□□■■■■■■■■■
M. tuberculosis NC 13270	UNITED STATES	1409	EAI6-BGD1	1	+	-	+	Human	■■■■■■■■■■■■■■■■■■■ ■■■□■■■■■□□□□■□■■□■□■■■■
M. tuberculosis HD6 13253	UNITED STATES	1	Beijing	1	+	-	-	Human	□□□□□□□□□□□□□□□□□□□ □□□□□□□□□□□□□□□■■■■■■■■■
M. tuberculosis W 13161	UNITED STATES	1	Beijing	1	+	-	-	Human	□□□□□□□□□□□□□□□□□□□ □□□□□□□□□□□□□□□■■■■■■■■■
M. tuberculosis HE8 14565	UNITED STATES	1	Beijing	1	+	-	-	Human	□□□□□□□□□□□□□□□□□□□ □□□□□□□□□□□□□□□■■■■■■■■■
M. tuberculosis DN3 14222	UNITED STATES	1	Beijing	1	+	-	-	Human	□□□□□□□□□□□□□□□□□□□ □□□□□□□□□□□□□□□■■■■■■■■■
M. tuberculosis 14214 001	UNITED STATES	1	Beijing	1	+	-	-	Human	□□□□□□□□□□□□□□□□□□□ □□□□□□□□□□□□□□□■■■■■■■■■
M. tuberculosis IP12 14157	UNITED STATES	19	EAI2-Manilla	1	+	-	+	Human	■■□■■■■■■■■■■■■■■■■ □□■■■■■■■□□□□■□■■■■■■■■■
M. tuberculosis 14454 001	UNITED STATES	1	Beijing	1	+	-	-	Human	□□□□□□□□□□□□□□□□□□□ □□□□□□□□□□□□□□□■■■■■■■■■
M. tuberculosis IK01 13701	UNITED STATES	138	EAI5	1	+	-	+	Human	■■■■■■■■■■■■■■■■■■■ ■■■■■■■■■□□□□■□■■■■■□□□□
M. tuberculosis N4 13664	UNITED STATES	1	Beijing	1	+	-	-	Human	□□□□□□□□□□□□□□□□□□□ □□□□□□□□□□□□□□□■■■■■■■■■
M. tuberculosis N16 13539	UNITED STATES	1	Beijing	1	+	-	-	Human	□□□□□□□□□□□□□□□□□□□ □□□□□□□□□□□□□□□■■■■■■■■■
M. tuberculosis KF15 14862	UNITED STATES	19	EAI2-Manilla	1	+	-	+	Human	■■□■■■■■■■■■■■■■■■■ □□■■■■■■■□□□□■□■■■■■■■■■
M. tuberculosis MB5 14868	UNITED STATES	26	CAS1-Delhi	1	+	-	-	Human	■■■□□□□■■■■■■■■■■■■ ■■■□□□□□□□□□□□□■■■■■■■■■
M. tuberculosis NU 13984	UNITED STATES	26	CAS1-Delhi	1	+	-	-	Human	■■■□□□□■■■■■■■■■■■■ ■■■□□□□□□□□□□□□■■■■■■■■■
M. tuberculosis MB4 13957	UNITED STATES	26	CAS1-Delhi	1	+	-	-	Human	■■■□□□□■■■■■■■■■■■■ ■■■□□□□□□□□□□□□■■■■■■■■■
M. tuberculosis CDC1551	UNITED STATES	549	X3	2	+	+	-	Human	■■■□□□□□□□□□■■■■■□■ ■■■□■■■■■■■■■□□□□■■■■■■■
M. tuberculosis Erdman	UNKNOWN	orphan	orphan	2	+	+	-	Human	■■■■■■■■■■■■■■■■■■■ ■■■■■■□□□□□■□■□□□■■■■■■■
M. tuberculosis 20010443	ALGERIA	50	Haarlem3	2	+	+	-	Human	■■■■■■■■■■■■■■■■■■■ ■■■■■■■■■■■□■□□□□■■■■■■■
M. tuberculosis 20020276	FRANCE. METROPOLITAN	50	Haarlem3	2	+	+	-	Human	■■■■■■■■■■■■■■■■■■■ ■■■■■■■■■■■□■□□□□■■■■■■■
M. tuberculosis 20020494	PORTUGAL	50	Haarlem3	2	+	+	-	Human	■■■■■■■■■■■■■■■■■■■ ■■■■■■■■■■■□■□□□□■■■■■■■

M. tuberculosis 20030442	RWANDA	128	T2	2	+	-	-	Human	■■□□■■■■■■■■■■■■□■■ ■■■■■■■■■■■■■□□□□■■■□■■□
M. tuberculosis 20020800	UNKNOWN	53	T1	2	+	-	-	Human	■■■■■■■■■■■■■■■■■■■ ■■■■■■■■■■■■■□□□□■■■■■■■
M. tuberculosis 20030007	FRANCE. METROPOLITAN	53	T1	2	+	-	-	Human	■■■■■■■■■■■■■■■■■■■ ■■■■■■■■■■■■■□□□□■■■■■■■
M. tuberculosis 20030507	Africa	60	LAM 4	2	+	-	-	Human	■■■■■■■■■■■■■■■■■■■ ■□□□□■■■■■■■■□□□□■■■□■■■
M. tuberculosis 386	SOUTH AFRICA	53	T1	2	+	+	-	Human	■■■■■■■■■■■■■■■■■■■ ■■■■■■■■■■■■■□□□□■■■■■■■
M. tuberculosis 166	SOUTH AFRICA	nd	nd	2	+	+	-	Human	nd
M. tuberculosis 1430	SOUTH AFRICA	2307	T2	2	+	-	-	Human	■■■■■■■■■■■■□□□□■■■ ■■■■■■■■■□■■■□□□□■■■□■■■
M. tuberculosis 113	SOUTH AFRICA	1353	U	2	+	-	-	Human	■■■■■■■■■■■■■■■■■■■ ■■■■■■□□□□□□□□□□□□□□□□□□
M. tuberculosis 913	SOUTH AFRICA	39	T4-CEU1	2	+	+	-	Human	■■■■■■■■■■■■■■■■■■□ ■■■□□■■■■■■■■□□□□■□□■■■■
M. tuberculosis 103	SOUTH AFRICA	orphan	orphan	2	+	+	-	Human	■■■■■■■■■■■□□□□□■■□ □□□□■■■■■■■■■□□□□■■□□■■■
M. tuberculosis 1297	SOUTH AFRICA	nd	nd	2	+	-	-	Human	nd
M. tuberculosis 494	SOUTH AFRICA	4	LAM3/S	2	+	-	-	Human	□□□□□□□□□□□□□□□□□□□ □□□□□■■■■■■■■□□□□■■■■■■■
M. tuberculosis 1245	SOUTH AFRICA	20	LAM1	2	+	-	-	Human	■■□■■■■■■■■■■■■■■■■ ■□□□□■■■■■■■■□□□□■■■■■■■
M. tuberculosis 1029	SOUTH AFRICA	orphan	nd	2	+	-	-	Human	■■■■■□□□□■■■■■■□□□□ □□■□□■■■■■■□□□□□□■■■■■■■
M. tuberculosis 107	SOUTH AFRICA	nd	nd	2	+	-	-	Human	nd
M. tuberculosis 716	SOUTH AFRICA	117	T1	2	+	-	-	Human	■■■■■■■■■■■■■■□■■■■ ■■■■■■■■■■■■■□□□□■■■□■■■
M. tuberculosis 1329	SOUTH AFRICA	53	T1	2	+	+	-	Human	■■■■■■■■■■■■■■■■■■■ ■■■■■■■■■■■■■□□□□■■■■■■■
M. tuberculosis 2081	SOUTH AFRICA	1241	U	2	+	-	-	Human	■■■■■■■■■■■■■■■■■■■ ■□□□□■■■■■■□□□□□□■■■■■■■
M. tuberculosis 433	SOUTH AFRICA	34	S	2	+	-	-	Human	■■■■■■■■□□■■■■■■■■■ ■■■■■■■■■■■■■□□□□■■■■■■■
M. tuberculosis 198	SOUTH AFRICA	53	T1	2	+	-	-	Human	■■■■■■■■■■■■■■■■■■■ ■■■■■■■■■■■■■□□□□■■■■■■■
M. tuberculosis 51	SOUTH AFRICA	42	LAM9	2	+	-	-	Human	■■■■■■■■■■■■■■■■■■■ ■□□□□■■■■■■■■□□□□■■■■■■■
M. tuberculosis 1008	SOUTH AFRICA	nd	nd	2	+	+	-	Human	nd
M. tuberculosis 66	SOUTH AFRICA	832	T2-T3	2	+	+	-	Human	■■■■■■■■■■■■□■□■■■■ ■■■■■■■■■■■■■□□□□■■■□■■■
M. tuberculosis 538	SOUTH AFRICA	370	T1	2	+	-	-	Human	■■■■■■■■■■■■■■■■■■■ ■■■□□■■■■■■■■□□□□■□□■■■■
M. tuberculosis 1050	SOUTH AFRICA	42	LAM9	2	+	-	-	Human	■■■■■■■■■■■■■■■■■■■ ■□□□□■■■■■■■■□□□□■■■■■■■
M. tuberculosis 1570	SOUTH AFRICA	2306	U	2	+	-	-	Human	■■■■■■■■■■■■□■■■■■■ ■■■■■□□□□□□□□□□□□■■■■■■■
M. tuberculosis 1211	SOUTH AFRICA	815	LAM11_ZWE	2	+	-	-	Human	■■■■■■■■■■■■■■■■■■■ ■□□□□■■□□□□■■□□□□■■■□■■■
M. tuberculosis 1084	SOUTH AFRICA	33	LAM3	2	+	-	-	Human	■■■■■■■■□□□■■■■■■■■ ■□□□□■■■■■■■■□□□□■■■■■■■
M. tuberculosis 1039	SOUTH AFRICA	2300	U	2	+	-	-	Human	■■■■□□□■■■■■■■□□□□□ □□■□□■■■■■■□□□□□□■■■■■■■

M. tuberculosis 1509	SOUTH AFRICA	50	Haarlem3	2	+	+	-	Human	■■■■■■■■■■■■■■■■■■■ ■■■■■■■■■■■□■□□□□■■■■■■■
M. tuberculosis 1367	SOUTH AFRICA	37	T1	2	+	-	-	Human	■■■■■■■■■■■■□■■■■■■ ■■■■■■■■■■■■■□□□□■■■■■■■
M. tuberculosis 449	SOUTH AFRICA	92	X3	2	+	+	-	Human	■■■□□□□□□□□□■■■■■□■ ■■■■■■■■■■■■■□□□□■■■■■■■
M. tuberculosis 322	SOUTH AFRICA	33	LAM3	2	+	-	-	Human	■■■■■■■■□□□■■■■■■■■ ■□□□□■■■■■■■■□□□□■■■■■■■
M. tuberculosis 1891	SOUTH AFRICA	nd	nd	2	+	-	-	Human	nd
M. tuberculosis 915	SOUTH AFRICA	33	LAM3	2	+	-	-	Human	■■■■■■■■□□□■■■■■■■■ ■□□□□■■■■■■■■□□□□■■■■■■■
M. tuberculosis 100	SOUTH AFRICA	2307	T2	2	+	-	-	Human	■■■■■■■■■■■■□□□□■■■ ■■■■■■■■■□■■■□□□□■■■□■■■
M. tuberculosis 1423	SOUTH AFRICA	nd	nd	2	+	-	-	Human	nd
M. tuberculosis 1088	SOUTH AFRICA	53	T1	2	+	-	-	Human	■■■■■■■■■■■■■■■■■■■ ■■■■■■■■■■■■■□□□□■■■■■■■
M. tuberculosis 1841	SOUTH AFRICA	nd	nd	2	+	-	-	Human	nd
M. tuberculosis 856	SOUTH AFRICA	42	LAM9	2	+	-	-	Human	■■■■■■■■■■■■■■■■■■■ ■□□□□■■■■■■■■□□□□■■■■■■■
M. tuberculosis 1595	SOUTH AFRICA	34	S	2	+	-	-	Human	■■■■■■■■□□■■■■■■■■■ ■■■■■■■■■■■■■□□□□■■■■■■■
M. tuberculosis 2091/03	BRAZIL	50	Haarlem3	2	+	-	-	Human	■■■■■■■■■■■■■■■■■■■ ■■■■■■■■■■■□■□□□□■■■■■■■
M. tuberculosis 556/03	BRAZIL	746	Haarlem3	2	+	+	-	Human	■■■■■■■■■■■■■■■■■■■ ■■■■■■■■■□■□■□□□□■■■■■■■
M. tuberculosis 1717/03	BRAZIL	50	Haarlem3	2	+	+	-	Human	■■■■■■■■■■■■■■■■■■■ ■■■■■■■■■■■□■□□□□■■■■■■■
M. tuberculosis 984	BRAZIL	nd	nd	2	+	+	-	Human	nd
M. tuberculosis 88/03	BRAZIL	50	Haarlem3	2	+	-	-	Human	■■■■■■■■■■■■■■■■■■■ ■■■■■■■■■■■□■□□□□■■■■■■■
M. tuberculosis 204/03	BRAZIL	50	Haarlem3	2	+	+	-	Human	■■■■■■■■■■■■■■■■■■■ ■■■■■■■■■■■□■□□□□■■■■■■■
M. tuberculosis 774/02	BRAZIL	2865	LAM9	2	+	+	-	Human	■■■■■■■■■■■■■■■■■■■ ■□□□□■■■■□□■■□□□□■■■■■■■
M. tuberculosis 1851/03	BRAZIL	50	Haarlem3	2	+	+	-	Human	■■■■■■■■■■■■■■■■■■■ ■■■■■■■■■■■□■□□□□■■■■■■■
M. tuberculosis 2196/03	BRAZIL	47	Haarlem1	2	+	+	-	Human	■■■■■■■■■■■■■■■■■■■ ■■■■■■□□□□□□■□□□□■■■■■■■
M. tuberculosis 2096/03	BRAZIL	47	Haarlem1	2	+	-	-	Human	■■■■■■■■■■■■■■■■■■■ ■■■■■■□□□□□□■□□□□■■■■■■■
M. tuberculosis 1741/03	BRAZIL	2866	Haarlem1	2	+	+	-	Human	■■■■■■■■■■■■□□■■■■■ ■■■■■■□□□□□□■□□□□■■■■■■■
M. tuberculosis 722/03	BRAZIL	33	LAM3	2	+	-	-	Human	■■■■■■■■□□□■■■■■■■■ ■□□□□■■■■■■■■□□□□■■■■■■■
M. tuberculosis BW33 13180	PUERTO RICO	50	Haarlem3	2	+	-	-	Human	■■■■■■■■■■■■■■■■■■■ ■■■■■■■■■■■□■□□□□■■■■■■■
M. tuberculosis BU7 12052	UNITED STATES	556	T1	2	+	+	-	Human	■■■■■■□■■■■□□□□□■■■ ■■■■■■□□□■■■■□□□□■■■■■■■
M. tuberculosis AU4 9158	UNITED STATES	557	Haarlem1	2	+	-	-	Human	■■■■■■□■■■■■■■■■■■■ ■■■■■■□□□□□□■□□□□■■■■■■■
M. tuberculosis H2 6510	UNITED STATES	119	X1	2	+	+	-	Human	■■■■■■■■■■■■■■■■■□■ ■■■■■■■■■■■■■□□□□■■■■■■■
M. tuberculosis H 725	UNITED STATES	137	X2	2	+	+	-	Human	■■■■■■■■■■■■■■■■■□■ ■■■■■■■■■■■■■□□□□■■□□□□■

M. tuberculosis C 10367	UNITED STATES	91	X3	2	+	+	-	Human	■■■□□□□□□□□□□■■■■□■ ■■■■■■■■■■■■■□□□□■■■■■■■
M. tuberculosis BE 10225	UNITED STATES	38	X2	2	+	+	-	Human	■■■■■■■■■■■■■■■■■□■ □□□□□■■■■■■■■□□□□■■□□□□■
M. tuberculosis V 9756	UNITED STATES	119	X1	2	+	+	-	Human	■■■■■■■■■■■■■■■■■□■ ■■■■■■■■■■■■■□□□□■■■■■■■
M. tuberculosis I 10870	UNITED STATES	91	X3	2	+	+	-	Human	■■■□□□□□□□□□□■■■■□■ ■■■■■■■■■■■■■□□□□■■■■■■■
M. tuberculosis CS3 10367	UNITED STATES	2506	LAM2	2	+	-	-	Human	■■□■■■■■■■■■■□■■■■■ ■□□□□■■■■■■■■□□□□■■■■■■■
M. tuberculosis AI10 11797	RUSSIA	42	LAM9	2	+	+	-	Human	■■■■■■■■■■■■■■■■■■■ ■□□□□■■■■■■■■□□□□■■■■■■■
M. tuberculosis P 1618	UNITED STATES	42	LAM9	2	+	-	-	Human	■■■■■■■■■■■■■■■■■■■ ■□□□□■■■■■■■■□□□□■■■■■■■
M. tuberculosis CC3 12496	UNITED STATES	33	LAM3	2	+	-	-	Human	■■■■■■■■□□□■■■■■■■■ ■□□□□■■■■■■■■□□□□■■■■■■■
M. tuberculosis Q5 12940	UNITED STATES	93	LAM5	2	+	-	-	Human	■■■■■■■■■■■■□■■■■■■ ■□□□□■■■■■■■■□□□□■■■■■■■
M. tuberculosis BM5 13626	MEXICO	406	Beijing Like	2	+	+	-	Human	□□□□□□□□□□□□□□□□□□□ □□□□□□□□□□□□□□□□□■■■□■■■
M. tuberculosis HJ 12217	CZECH REPUBLIC	61	LAM10_CAM	2	+	-	-	Human	■■■■■■■■■■■■■■■■■■■ ■■■□□□■■■■■■■□□□□■■■■■■■
M. tuberculosis BE3 10438	INDIA	53	T1	2	+	-	-	Human	■■■■■■■■■■■■■■■■■■■ ■■■■■■■■■■■■■□□□□■■■■■■■
M. tuberculosis LL 12758	RUSSIA	50	Haarlem3	2	+	-	-	Human	■■■■■■■■■■■■■■■■■■■ ■■■■■■■■■■■□■□□□□■■■■■■■
M. tuberculosis KQ15 12657	RUSSIA	50	Haarlem3	2	+	-	-	Human	■■■■■■■■■■■■■■■■■■■ ■■■■■■■■■■■□■□□□□■■■■■■■
M. tuberculosis Cb3.3	UNITED STATES	197	X3	2	+	+	-	Human	■■■□□□□□□□□□□■■■■□■ ■■■■■■■■■■■■■□□□□■■■□■■■
M. tuberculosis 133/92	UGANDA	584	T2	2	+	-	-	Human	■■■■■■■■■■■■■■■■□■■ ■■■■■■■■■■■■■□□□□■■■□■■■
M. tuberculosis 178/92	UGANDA	2340	T2	2	+	-	-	Human	■■□□■■■■■■■■■■■■■■■ ■■■■■■■■■■■■■□□□□■■■□■■□
M. tuberculosis 290/92	UGANDA	2867	T2	2	+	-	-	Human	■■■■■■■■■■■■■■■■■■■ ■■■■■■□■■■■■■□□□□■■■□■■■
M. tuberculosis 1167/92	UGANDA	orphan	X3	2	+	-	-	Human	□□□□□□□□□□□□□□□□□□■ □■■■■■■■■■■■■□□□□■■■□■■■
M. tuberculosis 21	UNITED STATES	197	X3	2	+	+	-	Human	■■■□□□□□□□□□□■■■■□■ ■■■■■■■■■■■■■□□□□■■■□■■■
*M. tuberculosis*22	UNITED STATES	orphan	LAM5	2	+	-	-	Human	■■■■■■■■■■■■□■■■■■■ ■□□□□■■■■■■□□□□□□□■■■■■■
M. tuberculosis 1918/02	TUNISIA	50	Haarlem3	2	+	+	-	Human	■■■■■■■■■■■■■■■■■■■ ■■■■■■■■■■■□■□□□□■■■■■■■
M. tuberculosis 1612/02	TUNISIA	943	T2-T4	2	+	-	-	Human	■■■■■■■■■■■■■■■■■■■ ■■■□■■■■■■■■■□□□□■■■□■■■
M. tuberculosis 859/02	TUNISIA	50	Haarlem3	2	+	+	-	Human	■■■■■■■■■■■■■■■■■■■ ■■■■■■■■■■■□■□□□□■■■■■■■
M. tuberculosis 1723/04	TUNISIA	42	LAM9	2	+	+	-	Human	■■■■■■■■■■■■■■■■■■■ ■□□□□■■■■■■■■□□□□■■■■■■■
M. tuberculosis 228/02	TUNISIA	50	Haarlem3	2	+	+	-	Human	■■■■■■■■■■■■■■■■■■■ ■■■■■■■■■■■□■□□□□■■■■■■■
M. tuberculosis 1156/02	TUNISIA	47	Haarlem1	2	+	+	-	Human	■■■■■■■■■■■■■■■■■■■ ■■■■■■□□□□□□■□□□□■■■■■■■
M. tuberculosis 930/03	TUNISIA	50	Haarlem3	2	+	-	-	Human	■■■■■■■■■■■■■■■■■■■ ■■■■■■■■■■■□■□□□□■■■■■■■
M. tuberculosis 1695/01	TUNISIA	50	Haarlem3	2	+	-	-	Human	■■■■■■■■■■■■■■■■■■■ ■■■■■■■■■■■□■□□□□■■■■■■■

M. tuberculosis 1362/04	TUNISIA	53	T1	2	+	-	-	Human	■■■■■■■■■■■■■■■■■■■ ■■■■■■■■■■■■■□□□□■■■■■■■
M. tuberculosis 1181/02	TUNISIA	42	LAM9	2	+	+	-	Human	■■■■■■■■■■■■■■■■■■■ ■□□□□■■■■■■■■□□□□■■■■■■■
M. tuberculosis 2389/01	TUNISIA	50	Haarlem3	2	+	+	-	Human	■■■■■■■■■■■■■■■■■■■ ■■■■■■■■■■■□■□□□□■■■■■■■
M. tuberculosis 1924/01	TUNISIA	50	Haarlem3	2	+	+	-	Human	■■■■■■■■■■■■■■■■■■■ ■■■■■■■■■■■□■□□□□■■■■■■■
M. tuberculosis 1394/03	TUNISIA	60	LAM4	2	+	+	-	Human	■■■■■■■■■■■■■■■■■■■ ■□□□□■■■■■■■■□□□□■■■□■■■
M. tuberculosis 2510/03	TUNISIA	50	Haarlem3	2	+	+	-	Human	■■■■■■■■■■■■■■■■■■■ ■■■■■■■■■■■□■□□□□■■■■■■■
M. tuberculosis 1902/03	TUNISIA	50	Haarlem3	2	+	+	-	Human	■■■■■■■■■■■■■■■■■■■ ■■■■■■■■■■■□■□□□□■■■■■■■
M. tuberculosis 1346/02	TUNISIA	2037	T1	2	+	-	-	Human	■■■■■■■■■■■■■■■■■■■ ■■■■■■□□□■■■■□□□□■■■■■■■
M. tuberculosis 2391/01	TUNISIA	516	T1	2	+	+	-	Human	■■■■■■■■■■■■■■■■■■■ ■■■■■■■■□■■■■□□□□■■■■■■■
M. tuberculosis 1782/02	TUNISIA	53	T1	2	+	+	-	Human	■■■■■■■■■■■■■■■■■■■ ■■■■■■■■■■■■■□□□□■■■■■■■
M. tuberculosis 1904/03	TUNISIA	53	T1	2	+	-	-	Human	■■■■■■■■■■■■■■■■■■■ ■■■■■■■■■■■■■□□□□■■■■■■■
M. tuberculosis 510/03	TUNISIA	47	Haarlem1	2	+	+	-	Human	■■■■■■■■■■■■■■■■■■■ ■■■■■■□□□□□□■□□□□■■■■■■■
M. tuberculosis 817/04	TUNISIA	47	Haarlem1	2	+	-	-	Human	■■■■■■■■■■■■■■■■■■■ ■■■■■■□□□□□□■□□□□■■■■■■■
M. tuberculosis 1220/02	TUNISIA	42	LAM9	2	+	-	-	Human	■■■■■■■■■■■■■■■■■■■ ■□□□□■■■■■■■■□□□□■■■■■■■
M. tuberculosis 1480/02	TUNISIA	47	Haarlem1	2	+	+	-	Human	■■■■■■■■■■■■■■■■■■■ ■■■■■■□□□□□□■□□□□■■■■■■■
M. tuberculosis 1083/03	TUNISIA	50	Haarlem3	2	+	-	-	Human	■■■■■■■■■■■■■■■■■■■ ■■■■■■■■■■■□■□□□□■■■■■■■
M. tuberculosis 1642/01	TUNISIA	orphan	orphan	2	+	+	-	Human	■■■■■■■■■■■■■■■■■■□ ■■□□□□□□□□□□□□□□□□□■■■■■
M. tuberculosis 1450/02	TUNISIA	50	Haarlem3	2	+	+	-	Human	■■■■■■■■■■■■■■■■■■■ ■■■■■■■■■■■□■□□□□■■■■■■■
M. tuberculosis 355/03	TUNISIA	50	Haarlem3	2	+	+	-	Human	■■■■■■■■■■■■■■■■■■■ ■■■■■■■■■■■□■□□□□■■■■■■■
M. tuberculosis 1641/03	TUNISIA	60	LAM4	2	+	+	-	Human	■■■■■■■■■■■■■■■■■■■ ■□□□□■■■■■■■■□□□□■■■□■■■
M. tuberculosis 282/02	TUNISIA	196	T1	2	+	-	-	Human	■■□■■■■■■■■■■■■■■■■ ■■■■■■■■■■■■■□□□□■■■■■■■
M. tuberculosis 172/02	TUNISIA	60	LAM4	2	+	+	-	Human	■■■■■■■■■■■■■■■■■■■ ■□□□□■■■■■■■■□□□□■■■□■■■
M. tuberculosis 1821/03	TUNISIA	50	Haarlem3	2	+	-	-	Human	■■■■■■■■■■■■■■■■■■■ ■■■■■■■■■■■□■□□□□■■■■■■■
M. tuberculosis 0635/04	TUNISIA	50	Haarlem3	2	+	+	-	Human	■■■■■■■■■■■■■■■■■■■ ■■■■■■■■■■■□■□□□□■■■■■■■
M. tuberculosis 820/03	TUNISIA	2868	T1	2	+	-	-	Human	■■■■■■■■■■■■■■■■■■□ ■■■■□□■■■■■■■□□□□■■■■■■■
M. tuberculosis 1033/02	TUNISIA	60	LAM4	2	+	-	-	Human	■■■■■■■■■■■■■■■■■■■ ■□□□□■■■■■■■■□□□□■■■□■■■
M. tuberculosis 1486/02	TUNISIA	50	Haarlem3	2	+	+	-	Human	■■■■■■■■■■■■■■■■■■■ ■■■■■■■■■■■□■□□□□■■■■■■■
M. tuberculosis 1707/02	TUNISIA	42	LAM9	2	+	-	-	Human	■■■■■■■■■■■■■■■■■■■ ■□□□□■■■■■■■■□□□□■■■■■■■
M. tuberculosis 1513/02	TUNISIA	2037	T1	2	+	+	-	Human	■■■■■■■■■■■■■■■■■■■ ■■■■■■□□□■■■■□□□□■■■■■■■

M. tuberculosis 2330/03	TUNISIA	42	LAM9	2	+	+	-	Human	■■■■■■■■■■■■■■■■■■■ ■□□□□■■■■■■■■□□□□■■■■■■■
M. tuberculosis 1282	TUNISIA	53	T1	2	+	-	-	Human	■■■■■■■■■■■■■■■■■■■ ■■■■■■■■■■■■■□□□□■■■■■■■
M. tuberculosis 1615/04	TUNISIA	47	Haarlem1	2	+	-	-	Human	■■■■■■■■■■■■■■■■■■■ ■■■■■■□□□□□□■□□□□■■■■■■■
*M. tuberculosis*2343/04	TUNISIA	53	T1	2	+	-	-	Human	■■■■■■■■■■■■■■■■■■■ ■■■■■■■■■■■■■□□□□■■■■■■■
M. tuberculosis 1188/01	TUNISIA	orphan	orphan	2	+	-	-	Human	■■■■■■■■■■■■■■■■■■□ ■□□□□■□□■■■■■□□□□■■■■■■■
M. tuberculosis 427/02	TUNISIA	orphan	orphan	2	+	-	-	Human	□■■■■■■■■■■■■■■■■■■ ■■□■■■■■■■■□■□□□□■■■■■■■
M. tuberculosis 462/03	TUNISIA	42	LAM9	2	+	-	-	Human	■■■■■■■■■■■■■■■■■■■ ■□□□□■■■■■■■■□□□□■■■■■■■
M. tuberculosis 1047/03	TUNISIA	50	Haarlem3	2	+	-	-	Human	■■■■■■■■■■■■■■■■■■■ ■■■■■■■■■■■□■□□□□■■■■■■■
M. tuberculosis 173/02	TUNISIA	36	Haarlem3	2	+	+	-	Human	■■■■■■■■■■■■□■■■■■■ ■■■■■■■■■■■□■□□□□■■■■■■■
M. tuberculosis 1852/03	TUNISIA	50	Haarlem3	2	+	-	-	Human	■■■■■■■■■■■■■■■■■■■ ■■■■■■■■■■■□■□□□□■■■■■■■
M. tuberculosis 2055/01	TUNISIA	47	Haarlem1	2	+	-	-	Human	■■■■■■■■■■■■■■■■■■■ ■■■■■■□□□□□□■□□□□■■■■■■■
M. tuberculosis 1685/02	TUNISIA	1064	LAM9	2	+	-	-	Human	■■■■■■□□■■■■■■■■■■■ ■□□□□■■■■■■■■□□□□■■■■■■■
M. tuberculosis 1198/03	TUNISIA	50	Haarlem3	2	+	+	-	Human	■■■■■■■■■■■■■■■■■■■ ■■■■■■■■■■■□■□□□□■■■■■■■
M. tuberculosis 2285/03	TUNISIA	1064	LAM9	2	+	-	-	Human	■■■■■■□□■■■■■■■■■■■ ■□□□□■■■■■■■■□□□□■■■■■■■
M. tuberculosis 562/01	TUNISIA	50	Haarlem3	2	+	+	-	Human	■■■■■■■■■■■■■■■■■■■ ■■■■■■■■■■■□■□□□□■■■■■■■
M. tuberculosis 1781/01	TUNISIA	50	Haarlem3	2	+	+	-	Human	■■■■■■■■■■■■■■■■■■■ ■■■■■■■■■■■□■□□□□■■■■■■■
M. tuberculosis 1209/03	TUNISIA	47	Haarlem1	2	+	-	-	Human	■■■■■■■■■■■■■■■■■■■ ■■■■■■□□□□□□■□□□□■■■■■■■
M. tuberculosis 1116/03	TUNISIA	42	LAM9	2	+	-	-	Human	■■■■■■■■■■■■■■■■■■■ ■□□□□■■■■■■■■□□□□■■■■■■■
M. tuberculosis 306/02	TUNISIA	62	Haarlem1	2	+	-	-	Human	■■■■■■■■■■■■■■■■■■■ ■■■■■■□□□□□□■□□□□■■■□■■■
M. tuberculosis 1338/02	TUNISIA	50	Haarlem3	2	+	-	-	Human	■■■■■■■■■■■■■■■■■■■ ■■■■■■■■■■■□■□□□□■■■■■■■
M. tuberculosis 875/01	TUNISIA	60	LAM4	2	+	+	-	Human	■■■■■■■■■■■■■■■■■■■ ■□□□□■■■■■■■■□□□□■■■□■■■
M. tuberculosis 2301/01	TUNISIA	42	LAM9	2	+	+	-	Human	■■■■■■■■■■■■■■■■■■■ ■□□□□■■■■■■■■□□□□■■■■■■■
M. tuberculosis 1045/03	TUNISIA	50	Haarlem3	2	+	+	-	Human	■■■■■■■■■■■■■■■■■■■ ■■■■■■■■■■■□■□□□□■■■■■■■
M. tuberculosis 1822/03	TUNISIA	1064	LAM9	2	+	-	-	Human	■■■■■■□□■■■■■■■■■■■ ■□□□□■■■■■■■■□□□□■■■■■■■
M. tuberculosis 789/02	TUNISIA	42	LAM9	2	+	-	-	Human	■■■■■■■■■■■■■■■■■■■ ■□□□□■■■■■■■■□□□□■■■■■■■
M. tuberculosis 1759/02	TUNISIA	42	LAM9	2	+	+	-	Human	■■■■■■■■■■■■■■■■■■■ ■□□□□■■■■■■■■□□□□■■■■■■■
M. tuberculosis 114/02	TUNISIA	1166	T1	2	+	-	-	Huma n	■■■■■■■■■□■■■■■■■■■ ■■■■■■■■■■■■■□□□□■■■■■■■
M. tuberculosis 428/02	TUNISIA	53	T1	2	+	-	-	Human	■■■■■■■■■■■■■■■■■■■ ■■■■■■■■■■■■■□□□□■■■■■■■
M. tuberculosis 1788/03	TUNISIA	42	LAM9	2	+	-	-	Human	■■■■■■■■■■■■■■■■■■■ ■□□□□■■■■■■■■□□□□■■■■■■■

M. tuberculosis 2239/02	TUNISIA	50	Haarlem3	2	+	-	-	Human	■■■■■■■■■■■■■■■■■■■ ■■■■■■■■■■■□■□□□□■■■■■■■
M. tuberculosis 1342/03	TUNISIA	883	Haarlem1	2	+	+	-	Human	■■■■■■■■■■■■■■■■■■■ ■■■□■■□□□□□□■□□□□■■■■■■■
M. tuberculosis 1760/02	TUNISIA	42	LAM9	2	+	-	-	Human	■■■■■■■■■■■■■■■■■■■ ■□□□□■■■■■■■■□□□□■■■■■■■
M. tuberculosis 741/02	TUNISIA	42	LAM9	2	+	-	-	Human	■■■■■■■■■■■■■■■■■■■ ■□□□□■■■■■■■■□□□□■■■■■■■
M. tuberculosis 1315/02	TUNISIA	50	Haarlem3	2	+	+	-	Human	■■■■■■■■■■■■■■■■■■■ ■■■■■■■■■■■□■□□□□■■■■■■■
M. tuberculosis 1638/03	TUNISIA	883	Haarlem1	2	+	-	-	Human	■■■■■■■■■■■■■■■■■■■ ■■■□■■□□□□□□■□□□□■■■■■■■
M. tuberculosis 1978/02	TUNISIA	2869	Haarlem1	2	+	-	-	Human	□■■■■■■■■■■■■■■■■■□ ■■■■□■□□□□□□■□□□□■■■■■■■
M. tuberculosis 738/02	TUNISIA	50	Haarlem3	2	+	+	-	Human	■■■■■■■■■■■■■■■■■■■ ■■■■■■■■■■■□■□□□□■■■■■■■
M. tuberculosis 1673/02	TUNISIA	orphan	orphan	2	+	-	-	Human	■■■■■■□□□■■■■■■■■■■ ■■■■■□□□□■■■■□□□□■■■■■■■
M. tuberculosis 2398/01	TUNISIA	50	Haarlem3	2	+	-	-	Human	■■■■■■■■■■■■■■■■■■■ ■■■■■■■■■■■□■□□□□■■■■■■■
M. tuberculosis 1671/02	TUNISIA	50	Haarlem3	2	+	+	-	Human	■■■■■■■■■■■■■■■■■■■ ■■■■■■■■■■■□■□□□□■■■■■■■
M. tuberculosis 662/03	TUNISIA	orphan	orphan	2	+	-	-	Human	■■■■■■■■■■■■■■■■■■□ ■□■■■■□□□□□□■□□□□■■■■■■■
M. tuberculosis 1360/04	TUNISIA	50	Haarlem3	2	+	-	-	Human	■■■■■■■■■■■■■■■■■■■ ■■■■■■■■■■■□■□□□□■■■■■■■
M. tuberculosis 2331/03	TUNISIA	orphan	orphan	2	+	-	-	Human	■■■■■■■■■■■■□□□□■■■ ■■■■■■■■■■■□■□□□□■■■■■■■
M. tuberculosis 492/02	TUNISIA	50	Haarlem3	2	+	+	-	Human	■■■■■■■■■■■■■■■■■■■ ■■■■■■■■■■■□■□□□□■■■■■■■
M. tuberculosis 490/02	TUNISIA	47	Haarlem1	2	+	+	-	Human	■■■■■■■■■■■■■■■■■■■ ■■■■■■□□□□□□■□□□□■■■■■■■
M. tuberculosis 381/02	TUNISIA	50	Haarlem3	2	+	-	-	Human	■■■■■■■■■■■■■■■■■■■ ■■■■■■■■■■■□■□□□□■■■■■■■
M. tuberculosis 1053/03	TUNISIA	orphan	orphan	2	-	-	-	Human	■■■■■■■■■■■■■■■□□■□ □□□□□■■■■■■■■□□□□■■■■■■■
M. tuberculosis 980/03	TUNISIA	orphan	orphan	2	-	-	-	Human	■■■□■■■■■■■■■□□□□■□ □□□□□■■■■■■■■□□□□■■■■■■■
M. tuberculosis 2148/03	TUNISIA	2868	T1	2	+	+	-	Human	■■■■■■■■■■■■■■■■■■□ ■■■■□□■■■■■■■□□□□■■■■■■■
M. tuberculosis 2442/03	TUNISIA	orphan	orphan	2	+	-	-	Human	□■■■■■■■■□■■■■■■■■■ ■■■■■■■■■■■□■□□□□■■■■■■■
M. tuberculosis 1835/01	TUNISIA	50	Haarlem3	2	+	+	-	Human	■■■■■■■■■■■■■■■■■■■ ■■■■■■■■■■■□■□□□□■■■■■■■
M. tuberculosis 874/01	TUNISIA	42	LAM9	2	+	-	-	Human	■■■■■■■■■■■■■■■■■■■ ■□□□□■■■■■■■■□□□□■■■■■■■
M. tuberculosis 1328/02	TUNISIA	42	LAM9	2	+	-	-	Human	■■■■■■■■■■■■■■■■■■■ ■□□□□■■■■■■■■□□□□■■■■■■■
M. tuberculosis 2150/03	TUNISIA	42	LAM9	2	+	+	-	Human	■■■■■■■■■■■■■■■■■■■ ■□□□□■■■■■■■■□□□□■■■■■■■
M. tuberculosis 1968/01	TUNISIA	53	T1	2	+	-	-	Human	■■■■■■■■■■■■■■■■■■■ ■■■■■■■■■■■■■□□□□■■■■■■■
M. tuberculosis 1978/03	TUNISIA	2869	Haarlem1	2	+	-	-	Human	□■■■■■■■■■■■■■■■■■□ ■■■■□■□□□□□□■□□□□■■■■■■■
M. tuberculosis 2114/02	TUNISIA	53	T1	2	+	-	-	Human	■■■■■■■■■■■■■■■■■■■ ■■■■■■■■■■■■■□□□□■■■■■■■
M. tuberculosis 1090/02	TUNISIA	orphan	orphan	2	+	+	-	Human	■■■■■■■■■■■■■■■■■■■ ■■□□□■■■■■■■□□□□■■■■■■■□

M. tuberculosis 1961/02	TUNISIA	53	T1	2	+	+	-	Human	■■■■■■■■■■■■■■■■■■■ ■■■■■■■■■■■■■□□□□■■■■■■■
M. tuberculosis 573/03	TUNISIA	47	Haarlem1	2	+	-	-	Human	■■■■■■■■■■■■■■■■■■■ ■■■■■■□□□□□□■□□□□■■■■■■■
*M. tuberculosis*1679/02	TUNISIA	53	T1	2	+	-	-	Human	■■■■■■■■■■■■■■■■■■■ ■■■■■■■■■■■■■□□□□■■■■■■■
M. tuberculosis 52/02	TUNISIA	50	Haarlem3	2	+	+	-	Human	■■■■■■■■■■■■■■■■■■■ ■■■■■■■■■■■□■□□□□■■■■■■■
M. tuberculosis 1221/02	TUNISIA	50	Haarlem3	2	+	+	-	Human	■■■■■■■■■■■■■■■■■■■ ■■■■■■■■■■■□■□□□□■■■■■■■
M. tuberculosis 918/02	TUNISIA	orphan	orphan	2	+	-	-	Human	■■■■■■■■■■■■■■■■■■□ ■■■■□■□□□□□□■□□□□■■■■■■■
M. tuberculosis 1122/02	TUNISIA	42	LAM9	2	+	-	-	Human	■■■■■■■■■■■■■■■■■■■ ■□□□□■■■■■■■■□□□□■■■■■■■
M. tuberculosis 0043/05	TUNISIA	2037	T1	2	+	-	-	Human	■■■■■■■■■■■■■■■■■■■ ■■■■■■□□□■■■■□□□□■■■■■■■
M. tuberculosis 2364/03	TUNISIA	60	LAM4	2	+	-	-	Human	■■■■■■■■■■■■■■■■■■■ ■□□□□■■■■■■■■□□□□■■■□■■■
M. tuberculosis 1988/02	TUNISIA	2387	U	2	+	+	-	Human	■■■■■■■■■■■■■■■■■■■ ■■■■■■□□□□□□□□□□□□■■□■■■
M. tuberculosis 0372/04	TUNISIA	2870	T1	2	+	+	-	Human	□■■■■■■■■■■■■■■■■■■ ■■■■□■■■■■■■■■□□□□■■■■■■■
M. tuberculosis 0374/04	TUNISIA	53	T1	2	+	+	-	Human	■■■■■■■■■■■■■■■■■■■ ■■■■■■■■■■■■■□□□□■■■■■■■
M. tuberculosis 0624/04	TUNISIA	53	T1	2	+	+	-	Human	■■■■■■■■■■■■■■■■■■■ ■■■■■■■■■■■■■□□□□■■■■■■■
M. tuberculosis 0276/04	TUNISIA	2870	T1	2	+	+	-	Human	□■■■■■■■■■■■■■■■■■■ ■■■■□■■■■■■■■□□□□■■■■■■■
M. tuberculosis 0354/03	TUNISIA	2865	LAM9	2	+	-	-	Human	■■■■■■■■■■■■■■■■■■■ ■□□□□■■■■□□■■□□□□■■■■■■■
M. tuberculosis 1013/04	TUNISIA	50	Haarlem3	2	+	+	-	Human	■■■■■■■■■■■■■■■■■■■ ■■■■■■■■■■■□■□□□□■■■■■■■
M. tuberculosis 0360/02	TUNISIA	50	Haarlem3	2	+	+	-	Human	■■■■■■■■■■■■■■■■■■■ ■■■■■■■■■■■□■□□□□■■■■■■■
M. tuberculosis 1959/03	TUNISIA	50	Haarlem3	2	+	+	-	Human	■■■■■■■■■■■■■■■■■■■ ■■■■■■■■■■■□■□□□□■■■■■■■
M. tuberculosis 1909/02	TUNISIA	42	LAM9	2	+	-	-	Human	■■■■■■■■■■■■■■■■■■■ ■□□□□■■■■■■■■□□□□■■■■■■■
M. tuberculosis 1383/03	TUNISIA	42	LAM9	2	+	-	-	Human	■■■■■■■■■■■■■■■■■■■ ■□□□□■■■■■■■■□□□□■■■■■■■
M. tuberculosis 0847/03	TUNISIA	53	T1	2	+	+	-	Human	■■■■■■■■■■■■■■■■■■■ ■■■■■■■■■■■■■□□□□■■■■■■■
M. tuberculosis 0843/03	TUNISIA	orphan	orphan	2	+	+	-	Human	■■■■■■■■■□□■■■■■■■■ ■□□□□■■■■■■■■□□□□■■■■■■■
M. tuberculosis 1024/03	TUNISIA	50	Haarlem3	2	+	-	-	Human	■■■■■■■■■■■■■■■■■■■ ■■■■■■■■■■■□■□□□□■■■■■■■
M. tuberculosis 0776/03	TUNISIA	50	Haarlem3	2	+	+	-	Human	■■■■■■■■■■■■■■■■■■■ ■■■■■■■■■■■□■□□□□■■■■■■■
M. tuberculosis 0691/04	TUNISIA	50	Haarlem3	2	+	+	-	Human	■■■■■■■■■■■■■■■■■■■ ■■■■■■■■■■■□■□□□□■■■■■■■
M. tuberculosis 1598/02	TUNISIA	50	Haarlem3	2	+	+	-	Human	■■■■■■■■■■■■■■■■■■■ ■■■■■■■■■■■□■□□□□■■■■■■■
M. tuberculosis 1887/03	TUNISIA	60	LAM4	2	+	-	-	Human	■■■■■■■■■■■■■■■■■■■ ■□□□□■■■■■■■■□□□□■■■□■■■
M. tuberculosis 1835/04	TUNISIA	34	S	2	+	+	-	Human	■■■■■■■■□□■■■■■■■■■ ■■■■■■■■■■■■■□□□□■■■■■■■
M. tuberculosis 2350/03	TUNISIA	53	T1	2	+	+	-	Human	■■■■■■■■■■■■■■■■■■■ ■■■■■■■■■■■■■□□□□■■■■■■■

M. tuberculosis 1209/03	TUNISIA	50	Haarlem3	2	+	-	-	Human	■■■■■■■■■■■■■■■■■■■ ■■■■■■■■■■■□■□□□□■■■■■■■
M. tuberculosis 1322/03	TUNISIA	42	LAM9	2	+	-	-	Human	■■■■■■■■■■■■■■■■■■■ ■□□□□■■■■■■■■□□□□■■■■■■■
M. tuberculosis 1236/02	TUNISIA	53	T1	2	+	-	-	Human	■■■■■■■■■■■■■■■■■■■ ■■■■■■■■■■■■■□□□□■■■■■■■
M. tuberculosis 2220/04	TUNISIA	42	LAM9	2	+	-	-	Human	■■■■■■■■■■■■■■■■■■■ ■□□□□■■■■■■■■□□□□■■■■■■■
M. tuberculosis 1511/03	TUNISIA	53	T1	2	+	+	-	Human	■■■■■■■■■■■■■■■■■■■ ■■■■■■■■■■■■■□□□□■■■■■■■
M. tuberculosis 2483/03	TUNISIA	42	LAM9	2	+	-	-	Human	■■■■■■■■■■■■■■■■■■■ ■□□□□■■■■■■■■□□□□■■■■■■■
M. tuberculosis 1763/03	TUNISIA	60	LAM4	2	+	-	-	Human	■■■■■■■■■■■■■■■■■■■ ■□□□□■■■■■■■■□□□□■■■□■■■
M. tuberculosis 1942/03	TUNISIA	42	LAM9	2	+	-	-	Human	■■■■■■■■■■■■■■■■■■■ ■□□□□■■■■■■■■□□□□■■■■■■■
M. tuberculosis 0883/03	TUNISIA	50	Haarlem3	2	+	+	-	Human	■■■■■■■■■■■■■■■■■■■ ■■■■■■■■■■■□■□□□□■■■■■■■
M. tuberculosis 2063/03	TUNISIA	42	LAM9	2	+	-	-	Human	■■■■■■■■■■■■■■■■■■■ ■□□□□■■■■■■■■□□□□■■■■■■■
M. tuberculosis 1429/03	TUNISIA	53	T1	2	+	-	-	Human	■■■■■■■■■■■■■■■■■■■ ■■■■■■■■■■■■■□□□□■■■■■■■
M. tuberculosis 1055/03	TUNISIA	53	T1	2	+	-	-	Human	■■■■■■■■■■■■■■■■■■■ ■■■■■■■■■■■■■□□□□■■■■■■■
M. tuberculosis 0817/03	TUNISIA	50	Haarlem3	2	+	-	-	Human	■■■■■■■■■■■■■■■■■■■ ■■■■■■■■■■■□■□□□□■■■■■■■
M. tuberculosis 1830/03	TUNISIA	42	LAM9	2	+	-	-	Human	■■■■■■■■■■■■■■■■■■■ ■□□□□■■■■■■■■□□□□■■■■■■■
M. tuberculosis 1090/05	TUNISIA	822	LAM9	2	+	+	-	Human	■■■■□■■■■■■■■■■■■■■ ■□□□□■■■■■■■■□□□□■■■■■■■
M. tuberculosis 1837/91	TUNISIA	42	LAM9	2	+	-	-	Human	■■■■■■■■■■■■■■■■■■■ ■□□□□■■■■■■■■□□□□■■■■■■■
M. tuberculosis 2190/91	TUNISIA	53	T1	2	+	-	-	Human	■■■■■■■■■■■■■■■■■■■ ■■■■■■■■■■■■■□□□□■■■■■■■
M. tuberculosis 1484	TUNISIA	53	T1	2	+	+	-	Human	■■■■■■■■■■■■■■■■■■■ ■■■■■■■■■■■■■□□□□■■■■■■■
M. tuberculosis 1872/02	TUNISIA	50	Haarlem3	2	-	-	-	Human	■■■■■■■■■■■■■■■■■■■ ■■■■■■■■■■■□■□□□□■■■■■■■
M. tuberculosis 295/03	TUNISIA	42	LAM9	2	-	-	-	Human	■■■■■■■■■■■■■■■■■■■ ■□□□□■■■■■■■■□□□□■■■■■■■
M. tuberculosis Lapaz	ARGENTINA	50	Haarlem3	2	+	+	-	Human	■■■■■■■■■■■■■■■■■■■ ■■■■■■■■■■■□■□□□□■■■■■■■
M. tuberculosis 462	ARGENTINA	2	Haarlem2	2	+	-	-	Human	□□□□□□□□□□□□□□□□□□□ □□□□□■□□□□□□■□□□□■■■■■■■
M. tuberculosis 825	LIBYA	50	Haarlem3	2	+	+	-	Human	■■■■■■■■■■■■■■■■■■■ ■■■■■■■■■■■□■□□□□■■■■■■■
M. tuberculosis 826	LIBYA	53	T1	2	+	+	-	Human	■■■■■■■■■■■■■■■■■■■ ■■■■■■■■■■■■■□□□□■■■■■■■
M. tuberculosis 827	LIBYA	50	Haarlem3	2	+	+	-	Human	■■■■■■■■■■■■■■■■■■■ ■■■■■■■■■■■□■□□□□■■■■■■■
M. tuberculosis 1177	LIBYA	2644	Haarlem3	2	+	-	-	Human	□□■■■■■■■■■■■■■■■■■ ■■■■■■■■■■■□■□□□□■■■■■■■
M. tuberculosis 1178	LIBYA	50	Haarlem3	2	+	+	-	Human	■■■■■■■■■■■■■■■■■■■ ■■■■■■■■■■■□■□□□□■■■■■■■
M. tuberculosis P6 13249	UNITED STATES	42	LAM 9	2	+	-	-	Human	■■■■■■■■■■■■■■■■■■■ ■□□□□■■■■■■■■□□□□■■■■■■■
M. tuberculosis H 10230	UNITED STATES	50	Haarlem3	2	+	+	-	Human	■■■■■■■■■■■■■■■■■■■ ■■■■■■■■■■■□■□□□□■■■■■■■

M. tuberculosis H6 10229	UNITED STATES	38	X2	2	+	+	-	Human	■■■■■■■■■■■■■■■■■□■ □□□□□■■■■■■■■□□□□■■□□□□■
M. tuberculosis AH 10212	UNITED STATES	119	X1	2	+	+	-	Human	■■■■■■■■■■■■■■■■■□■ ■■■■■■■■■■■■■□□□□■■■■■■■
M. tuberculosis 10206 BU7	UNITED STATES	2871	U	2	+	+	-	Human	■■■■■■■■■□□□□■■■■■■ ■■■■■■■■■■■■■□□□□■■□□□■■
M. tuberculosis I 10116	UNITED STATES	70	X3	2	+	+	-	Human	■■■□□□□□□□□□■■■■■□■ ■■■■■■■■■■■■■□□□□■■□■■■■
M. tuberculosis BW33 9943	UNITED STATES	50	Haarlem3	2	+	+	-	Human	■■■■■■■■■■■■■■■■■■■ ■■■■■■■■■■■□■□□□□■■■■■■■
M. tuberculosis CH12 9912	UNITED STATES	50	Haarlem3	2	+	+	-	Human	■■■■■■■■■■■■■■■■■■■ ■■■■■■■■■■■□■□□□□■■■■■■■
M. tuberculosis AX19 11048	UNITED STATES	2	Haarlem2	2	+	+	-	Human	□□□□□□□□□□□□□□□□□□□ □□□□□■□□□□□□■□□□□■■■■■■■
M. tuberculosis GO 11034	UNITED STATES	42	LAM 9	2	+	-	-	Human	■■■■■■■■■■■■■■■■■■■ ■□□□□■■■■■■■■□□□□■■■■■■■
M. tuberculosis DK21 10306	UNITED STATES	2871	U	2	+	+	-	Human	■■■■■■■■■□□□□■■■■■■ ■■■■■■■■■■■■■□□□□■■□□□■■
M. tuberculosis KO1 12394	UNITED STATES	34	S	2	+	-	-	Human	■■■■■■■■□□■■■■■■■■■ ■■■■■■■■■■■■■□□□□■■■■■■■
M. tuberculosis CH12 11904	UNITED STATES	99	Haarlem3	2	+	-	-	Human	■■■■□■■■■■■■■■■■■■■ ■■■■■■■■■■■□■□□□□■■■■■■■
M. tuberculosis C29 11668	UNITED STATES	50	Haarlem3	2	+	-	-	Human	■■■■■■■■■■■■■■■■■■■ ■■■■■■■■■■■□■□□□□■■■■■■■
M. tuberculosis AX 11648	UNITED STATES	2	Haarlem2	2	+	-	-	Human	□□□□□□□□□□□□□□□□□□□ □□□□□■□□□□□□■□□□□■■■■■■■
M. tuberculosis Q2 12863	UNITED STATES	93	LAM5	2	+	-	-	Human	■■■■■■■■■■■■□■■■■■■ ■□□□□■■■■■■■■□□□□■■■■■■■
M. tuberculosis 12857	UNITED STATES	33	LAM3	2	+	-	-	Human	■■■■■■■■□□□■■■■■■■■ ■□□□□■■■■■■■■□□□□■■■■■■■
M. tuberculosis AH 12751	UNITED STATES	119	X1	2	+	-	-	Human	■■■■■■■■■■■■■■■■■□■ ■■■■■■■■■■■■■□□□□■■■■■■■
M. tuberculosis AG8 12450	UNITED STATES	47	Haarlem1	2	+	+	-	Human	■■■■■■■■■■■■■■■■■■■ ■■■■■■□□□□□□■□□□□■■■■■■■
M. tuberculosis CS46 13494	UNITED STATES	2506	LAM2	2	+	-	-	Human	■■□■■■■■■■■■■□■■■■■ ■□□□□■■■■■■■■□□□□■■■■■■■
M. tuberculosis HJ7 13474	UNITED STATES	61	LAM10_CAM	2	+	-	-	Human	■■■■■■■■■■■■■■■■■■■ ■■■□□□■■■■■■■□□□□■■■■■■■
M. tuberculosis AU23 13468	UNITED STATES	47	Haarlem1	2	+	-	-	Human	■■■■■■■■■■■■■■■■■■■ ■■■■■■□□□□□□■□□□□■■■■■■■
M. tuberculosis KQ37 14255	UNITED STATES	orphan	LAM9	2	+	-		Human	■■■■■■■□□■■■■■■■■■■ ■□□□□■■■■□□□■□□□□■■■■■■■
M. tuberculosis C 14648	UNITED STATES	70	X 3	2	+	-	-	Human	■■■□□□□□□□□□■■■■■□■ ■■■■■■■■■■■■■□□□□■■□■■■■
M. tuberculosis H37Rv	UNKNOWN	451	H37Rv	3	-	-	-	Human	■■■■■■■■■■■■■■■■■■■ □□■■■■■■■■■■■□□□□■■■■■■■
M. tuberculosis H37Ra	UNKNOWN	451	H37Rv	3	-	-	-	Human	■■■■■■■■■■■■■■■■■■■ □□■■■■■■■■■■■□□□□■■■■■■■
M. tuberculosis 20030446	FRANCE. METROPOLITAN	41	LAM7_TUR	3	+	-	-	Human	■■■■■■■■■■■■■■■■■■■ □□□□□■□□■■■■■□□□□■■■■■■■
M. tuberculosis 20021370	FRANCE. METROPOLITAN	191	T1	3	+	-	-	Human	□□■■■■■■■■■■■■■■■■■ ■■■■■■■■■■■■■□□□□■■■■■■■
*M. tuberculosis*20021560	UNKNOWN	334	T1	3	+	-	-	Human	■□■■■■■■■■■■■■■■■■■ ■■■■■■■■■■■■■□□□□■■■■■■■
M. tuberculosis 20031050	FRANCE. METROPOLITAN	51	T1	3	+	-	-	Human	■■■■■■■■■■■■■■■■■■■ ■■■■■■■■■■■■■□□□□■■■□□□□
M. tuberculosis 889	SOUTH AFRICA	orphan	orphan	3	+	-	-	Human	■■■■■■■□□□□□□■■■■■■ ■□□□□■■■■■■■■□□□□■■■■■■■

M. tuberculosis 327	SOUTH AFRICA	53	T1	3	+	-	-	Human	■■■■■■■■■■■■■■■■■■■ ■■■■■■■■■■■■■□□□□■■■■■■■
M. tuberculosis 723	SOUTH AFRICA	245	T1	3	+	-	-	Human	■■■■■■■■■■■■■■■■■■■ ■■■■■■■■■■■■■□□□□■■□■■■■
M. tuberculosis 761	SOUTH AFRICA	nd	nd	3	+	-	-	Human	nd
M. tuberculosis 24	SOUTH AFRICA	53	T1	3	+	-	-	Human	■■■■■■■■■■■■■■■■■■■ ■■■■■■■■■■■■■□□□□■■■■■■■
M. tuberculosis 337	SOUTH AFRICA	53	T1	3	+	-	-	Human	■■■■■■■■■■■■■■■■■■■ ■■■■■■■■■■■■■□□□□■■■■■■■
M. tuberculosis 1109	SOUTH AFRICA	53	T1	3	+	-	-	Human	■■■■■■■■■■■■■■■■■■■ ■■■■■■■■■■■■■□□□□■■■■■■■
M. tuberculosis 1755	SOUTH AFRICA	53	T1	3	+	-	-	Human	■■■■■■■■■■■■■■■■■■■ ■■■■■■■■■■■■■□□□□■■■■■■■
M. tuberculosis 933	SOUTH AFRICA	209	LAM3	3	+	-	-	Human	■■■■■■■■□□□□□□■■■■■ ■□□□□■■■■■■■■□□□□■■■■■■■
M. tuberculosis 1012	SOUTH AFRICA	53	T1	3	+	-	-	Human	■■■■■■■■■■■■■■■■■■■ ■■■■■■■■■■■■■□□□□■■■■■■■
M. tuberculosis 2042	SOUTH AFRICA	53	T1	3	+	-	-	Human	■■■■■■■■■■■■■■■■■■■ ■■■■■■■■■■■■■□□□□■■■■■■■
M. tuberculosis 1956	SOUTH AFRICA	196	T1	3	+	-	-	Human	■■□■■■■■■■■■■■■■■■■ ■■■■■■■■■■■■■□□□□■■■■■■■
M. tuberculosis 1870	SOUTH AFRICA	53	T1	3	+	-	-	Human	■■■■■■■■■■■■■■■■■■■ ■■■■■■■■■■■■■□□□□■■■■■■■
M. tuberculosis (Nyiso)	UNITED STATES	451	T1	3	+	-	-	Human	■■■■■■■■■■■■■■■■■■■ □□■■■■■■■■■■■□□□□■■■■■■■
M. tuberculosis ATCC51910	UNITED STATES	144	T1	3	+	-	-	Human	■■■■■■□□□□□□□□□□□□□ □□□□□□■■■■■■■□□□□■■■■■■■
M. tuberculosis 1155/03	BRAZIL	51	T1	3	+	+	-	Human	■■■■■■■■■■■■■■■■■■■ ■■■■■■■■■■■■■□□□□■■■□□□□
M. tuberculosis 134/03	BRAZIL	58	T5_MAD2	3	+	-	-	Human	■■■■■■■■■■■■■■■■■■■ □■■□■■■■■■■■■□□□□■■■■■■■
M. tuberculosis 1526/03	BRAZIL	nd	nd	3	+	-	-	Human	nd
M. tuberculosis 132/03	BRAZIL	58	T5_MAD2	3	+	-	-	Human	■■■■■■■■■■■■■■■■■■■ □■■□■■■■■■■■■□□□□■■■■■■■
M. tuberculosis 599/03	BRAZIL	nd	nd	3	+	+	-	Human	nd
M. tuberculosis BF16 12776	UNITED STATES	53	T1	3	+	-	-	Human	■■■■■■■■■■■■■■■■■■■ ■■■■■■■■■■■■■□□□□■■■■■■■
M. tuberculosis BJ 13067	UNITED STATES	1223	S	3	+	-	-	Human	■■■■■■■■□□□□■■■■■■■ ■■■■■■■■■■■■■□□□□■■■■■■■
M. tuberculosis M33 8562	RUSSIA	orphan	orphan	3	+	-	-	Human	■■■■■■□□□□□□□□□□□■■ ■■■■■■■■■■■■■□□□□■■■■■■■
M. tuberculosis AF 9139	UNITED STATES	53	T1	3	+	-	-	Human	■■■■■■■■■■■■■■■■■■■ ■■■■■■■■■■■■■□□□□■■■■■■■
M. tuberculosis AF52 11074	UNITED STATES	53	T1	3	+	-	-	Human	■■■■■■■■■■■■■■■■■■■ ■■■■■■■■■■■■■□□□□■■■■■■■
M. tuberculosis AF50 11043	UNITED STATES	53	T1	3	+	-	-	Human	■■■■■■■■■■■■■■■■■■■ ■■■■■■■■■■■■■□□□□■■■■■■■
M. tuberculosis AF57 11914	UNITED STATES	53	T1	3	+	-	-	Human	■■■■■■■■■■■■■■■■■■■ ■■■■■■■■■■■■■□□□□■■■■■■■
M. tuberculosis 13022	UNITED STATES	52	T2	3	+	-	-	Human	■■■■■■■■■■■■■■■■■■■ ■■■■■■■■■■■■■□□□□■■■□■■■
M. tuberculosis BJ51 12878	UNITED STATES	53	T1	3	+	-	-	Human	■■■■■■■■■■■■■■■■■■■ ■■■■■■■■■■■■■□□□□■■■■■■■
M. tuberculosis 1547/03	BRAZIL	33	LAM3	3	+	-	-	Human	■■■■■■■■□□□■■■■■■■■ ■□□□□■■■■■■■■□□□□■■■■■■■

M. tuberculosis 474/03	BRAZIL	53	T1	3	+	-	-	Human	■■■■■■■■■■■■■■■■■■■ ■■■■■■■■■■■■■□□□□■■■■■■■
M. tuberculosis 332/03	BRAZIL	53	T1	3	+	-	-	Human	■■■■■■■■■■■■■■■■■■■ ■■■■■■■■■■■■■□□□□■■■■■■■
M. tuberculosis 1525/03	BRAZIL	53	T1	3	+	-	-	Human	■■■■■■■■■■■■■■■■■■■ ■■■■■■■■■■■■■□□□□■■■■■■■
M. tuberculosis 39/02	BRAZIL	53	T1	3	+	-	-	Human	■■■■■■■■■■■■■■■■■■■ ■■■■■■■■■■■■■□□□□■■■■■■■
M. tuberculosis 1102/02	BRAZIL	51	T1	3	+	-	-	Human	■■■■■■■■■■■■■■■■■■■ ■■■■■■■■■■■■■□□□□■■■□□□□
M. tuberculosis 678/03	BRAZIL	2872	T5_MAD2	3	+	-	-	Human	■■■■■■■■■■■■■■■■■■■ □■■□■■■■■■■■■□□□□■■■□□□□
M. tuberculosis 108/03	BRAZIL	2872	T5_MAD2	3	+	-	-	Human	■■■■■■■■■■■■■■■■■■■ □■■□■■■■■■■■■□□□□■■■□□□□
M. tuberculosis 134/03	BRAZIL	58	T5_MAD2	3	+	-	-	Human	■■■■■■■■■■■■■■■■■■■ □■■□■■■■■■■■■□□□□■■■■■■■
M. tuberculosis 555/03	BRAZIL	nd	nd	3	+	-	-	Human	nd
M. tuberculosis 1381/03	BRAZIL	205	T1	3	+	-	-	Human	■■■□■■■■■■■■■■■■■■■ ■■■■■■■■■■■■■□□□□■■■■■■■
M. tuberculosis 1575/03	BRAZIL	42	LAM9	3	+	-	-	Human	■■■■■■■■■■■■■■■■■■■ ■□□□□■■■■■■■■□□□□■■■■■■■
M. tuberculosis 979/02	BRAZIL	orphan	X3	3	+	+	-	Human	■■■■□□□□□□□□□■■■■■■ ■■■■■■■■■■■■■□□□□■■■□□□□
M. bovis 2122/97	UNITED KINGDOM	685	BOV	1	+	-	-	Cow	■■□■ ■□■□□□□□■■■□■■■■■■■■■■■ ■■■■■■■■■■■□□□□□
M. bovis 950065	FRANCE. METROPOLITAN	482	Bovis1_BCG	1	+	-	+	Human	■■□■■■■■□■■■■■■□■■■ ■■■■■■■■■■■■■■■■■■■□□□□□
M. bovis 950643	FRANCE. METROPOLITAN	orphan	orphan	1	+	-	+	Human	■■□□□■■■□□□■■■□□■■■ ■■■■■■■■■■■■■■■■□■■□□□□□
M. bovis 950774	FRANCE. METROPOLITAN	665	Bovis1	1	+	-	+	Human	■■□□□■■■□■■■■■■□■■■ ■■■■■■■■■■■■■■■■■■■□□□□□
M. bovis 951025	FRANCE. METROPOLITAN	481	Bovis1_BCG	1	+	-	+	Human	■■□■■■■■□■■■■■■□■■■ ■□■■■■■■■■■■■■■■■■■□□□□□
M. bovis 951065	FRANCE. METROPOLITAN	orphan	orphan	1	+	-	+	Human	■■□■■■■■□■■■■■■□■■□ □□□□□□■□□□□□□■■■■■■□□□□□
M. bovis 951186	FRANCE. METROPOLITAN	482	Bovis1	1	+	-	+	Human	■■□■■■■■□■■■■■■□■■■ ■■■■■■■■■■■■■■■■■■■□□□□□
M. bovis 951193	FRANCE. METROPOLITAN	nd	nd	1	+	-	+	Human	nd
M. bovis 951194	ALGERIA	481	Bovis1_BCG	1	+	-	+	Human	■■□■■■■■□■■■■■■□■■■ ■□■■■■■■■■■■■■■■■■■□□□□□
M. bovis 951333	FRANCE. METROPOLITAN	orphan	orphan	1	+	-	+	Human	■■□■□□□□□□□□□□□□■■■ ■■■■■■■■■■■■□■■■■■■□□□□□
M. bovis 951366	FRANCE. METROPOLITAN	482	Bovis1_BCG	1	+	-	+	Human	■■□■■■■■□■■■■■■□■■■ ■■■■■■■■■■■■■■■■■■■□□□□□
M. bovis 951670	UNKNOWN	orphan	orphan	1	+	-	+	Human	■■□□□■■■□■■■■■□□■■■ ■■■■■■■■■■■■■■■■■□■□□□□□
M. bovis 960273	FRANCE. METROPOLITAN	665	Bovis1	1	+	-	+	Human	■■□□□■■■□■■■■■■□■■■ ■■■■■■■■■■■■■■■■■■■□□□□□
M. bovis 961123	FRANCE. METROPOLITAN	482	Bovis1_BCG	1	+	-	+	Human	■■□■■■■■□■■■■■■□■■■ ■■■■■■■■■■■■■■■■■■■□□□□□
M. bovis 961350	FRANCE. METROPOLITAN	orphan	orphan	1	+	-	+	Human	□■□■■■■■□■■■■■□□■■■ ■■■■■■■■■■■■■■■■■■■□□□□□
M. bovis 970069	MOROCCO	684	Bovis1	1	+	-	+	Human	■■□■■□■■□■■■■■■□■■■ ■□■■■■■■■■■■■■■■■■■□□□□□
M. bovis 970071	MOROCCO	orphan	orphan	1	+	-	+	Human	■■□■■■□■□■■■■■□□■■■ ■■■■■■■■□□■■■■■■■■■□□□□□

M. bovis 970072	UNKNOWN	482	Bovis1_BCG	1	+	-	+	Human	■■□■■■■■□■■■■■■□■■■ ■■■■■■■■■■■■■■■■■■■□□□□□
M. bovis 970101	FRANCE. METROPOLITAN	orphan	orphan	1	+	-	+	Human	■■□■■■■■□■■■■■□□■■■ ■■■■■■■■■■■■□□□■■■■□□□□□
M. bovis 0041/02	TUNISIA	orphan	orphan	1	+	-	+	Human	■■□■■■■■■■■■■■■■■■■ ■■■■■■■■■■■■■■■■■■■□□□□□
M. bovis ATCC19210	UNITED STATES	691	Bovis1	1	+	-	+	Cow	■■□■■■■■□■□■■■■□■■■ ■■■■■■■■■■■■■■■■■■■□□□□□
M. bovis ATCC35725	UNKNOWN	691	Bovis1	1	+	-	+	Cow	■■□■■■■■□■□■■■■□■■■ ■■■■■■■■■■■■■■■■■■■□□□□□
M. bovis ATCC35726	UNKNOWN	2873	Bovis1	1	+	-	+	Cow	■■□■■■■■□■■■■■■□■■■ ■■■■■■■■■■■□□□□□□□■□□□□□
M. bovis ATCC35730	UNKNOWN	691	Bovis1	1	+	-	+	Cow	■■□■■■■■□■□■■■■□■■■ ■■■■■■■■■■■■■■■■■■■□□□□□
M. bovis BV0061	UNKNOWN	2874	Bovis1	1	+	-	+	nd	□■□■■□■□□□□□■■■□■■■ ■■□■■■■■■■■■■■■■■■■□□□□□
M. bovis BV0063	UNKNOWN	2874	Bovis1	1	+	-	+	nd	□■□■■□■□□□□□■■■□■■■ ■■□■■■■■■■■■■■■■■■■□□□□□
M. bovis M61760	UNITED STATES	2874	Bovis1	1	+	-	+	Human	□■□■■□■□□□□□■■■□■■■ ■■□■■■■■■■■■■■■■■■■□□□□□
M. bovis 71	BRAZIL	nd	nd	1	+	-	+	nd	nd
M. bovis 30	BRAZIL	nd	nd	1	+	-	+	nd	nd
M. bovis 10	BRAZIL	nd	nd	1	+	-	+	nd	nd
M. bovis 74	BRAZIL	nd	nd	1	+	-	+	nd	nd
M. bovis 46	BRAZIL	nd	nd	1	+	-	+	nd	nd
M. bovis 19	BRAZIL	nd	nd	1	+	-	+	nd	nd
M. bovis 11	BRAZIL	nd	nd	1	+	-	+	nd	nd
M. bovis 48	BRAZIL	nd	nd	1	+	-	+	nd	nd
M. bovis 3	BRAZIL	nd	nd	1	+	-	+	nd	nd
M. bovis 38	BRAZIL	nd	nd	1	+	-	+	nd	nd
M. bovis BCG r4-93	UNKNOWN	482	Bovis1_BCG	1	+	-	+	nd	■■□■■■■■□■■■■■■□■■■ ■■■■■■■■■■■■■■■■■■■□□□□□
M. bovis BCG BCGt	TUNISIA	482	Bovis1_BCG	1	+	-	+	nd	■■□■■■■■□■■■■■■□■■■ ■■■■■■■■■■■■■■■■■■■□□□□□
M. bovis BCG ATCC27290	DENMARK	482	Bovis1_BCG	1	+	-	+	nd	■■□■■■■■□■■■■■■□■■■ ■■■■■■■■■■■■■■■■■■■□□□□□
M. bovis BCG ATCC35732	SWEDEN	482	Bovis1_BCG	1	+	-	+	nd	■■□■■■■■□■■■■■■□■■■ ■■■■■■■■■■■■■■■■■■■□□□□□
M. bovis BCG	DENMARK	482	Bovis1_BCG	1	+	-	+	nd	■■□■■■■■□■■■■■■□■■■ ■■■■■■■■■■■■■■■■■■■□□□□□
M. africanum 951223	FRANCE. METROPOLITAN	orphan	orphan	1	+	-	+	Human	■■■■■■□□□■■■■■■■■■■ □□□□□■■■■■■■■■■■■■■□■■■■
M. africanum 960688	FRANCE. METROPOLITAN	187	AFRI_1	1	+	-	+	Human	■■■■■■□□□■■■■■■■■■■ ■■■■■■■■■■■■■□■■■■■□■■■■
M. africanum 940961	FRANCE. METROPOLITAN	181	AFRI_1	1	+	-	+	Human	■■■■■■□□□■■■■■■■■■■ ■■■■■■■■■■■■■■■■■■■□■■■■
M. africanum 951936	FRANCE. METROPOLITAN	326	AFRI_1	1	+	-	+	Human	■■■■■■□□□■■■■■■■■■■ ■■□□□■■■■■■■■■■■■■■□■■■■

M. africanum 961262	FRANCE. METROPOLITAN	orphan	orphan	1	+	-	+	Human	■■■■■■□□□■■■■■■■■■■ ■■■■■■■■■■■■■■■■■□□□■■■■
M. africanum 970133	FRANCE. METROPOLITAN	326	AFRI_1	1	+	-	+	Human	■■■■■■□□□■■■■■■■■■■ ■■□□□■■■■■■■■■■■■■■□■■■■
M. africanum 970877	COTE D IVOIRE	orphan	orphan	1	+	-	+	Human	■□■■■■□□□■■■■■■□■■■ ■■■■■■■■■■■■■■■■■■■□■■■■
M. africanum 14003009	SENEGAL	2875	AFRI_2	1	+	-	+	Human	■■■■■■■□□□□□■■□■■■■ ■□□□□■■■■■■■■□□□■□□□□■■■
M. africanum 961389	FRANCE. METROPOLITAN	orphan	orphan	1	+	-	+	Human	■■■■■■■□□□□□■■□■■■■ □□□□□■■■■■■■■■■■■□□□□■■■
M. africanum 970869	COTE D IVOIRE	orphan	orphan	1	+	-	+	Human	■□■■■■■□□□□□■■■■■■■ ■□□□□■■■■■■■■■■■■□□□□■■■
M. africanum 970872	COTE D IVOIRE	330	AFRI	1	+	-	+	Human	■■■■■■■□□□□□■■■■■■■ ■□□□□■■■■■■■■■■■■□□□□■■■
M. africanum 970874	COTE D IVOIRE	orphan	orphan	1	+	-	+	Human	■□□■■■■□□□□□■■■■■■■ ■□□□□■■■□□■■■■■■■□□□□■■■
M. africanum 980698	CAMEROON	2876	AFRI_2	1	+	-	+	Human	■■■■■■■□□□□□■■□■■■■ ■□□□□■■□■■■■■□■■■□□□□■■■
M. africanum 14003041	CAMEROON	orphan	orphan	1	+	-	+	Human	□□□□□□□□□□□□□□□□□□□ □□□□□□■■■■■□■■■■■□□□□■■■
M. africanum 970866	COTE D IVOIRE	orphan	orphan	1	+	-	+	Human	■■■■■■■□□□□□■■■■■■■ ■□□□□□■□□□□□□□□□□□□□□□□□
M. africanum 980338	CAMEROON	856	AFRI_3	1	+	-	+	Human	■■■■■■■□□□□□■■■■■■■ ■■■■■□□□□□□□□□■□■□□□■■■■
M. africanum 980699	CAMEROON	orphan	orphan	1	+	-	+	Human	■■■■■■■□□□□□■■□■■■■ ■■■■■■■□□□□□□□□□■■■□■■■■
M. africanum 980702	CAMEROON	2877	AFRI_3	1	+	-	+	Human	■□■■■■■□□□□□■■□□■■■ ■□■■■□□□□□□□□□■■■□□□■■■■
M. africanum 981444	CAMEROON	orphan	orphan	1	+	-	+	Human	■■■■■■□□□□□□□□□□■■■ ■□□□□■□□□■■■■■■■■■■□■■■■
M. africanum 981446	CAMEROON	858	AFRI_3	1	+	-	+	Human	■■■■■■■□□□□□■■□■■■■ ■■■■■□□□□□□□□□■□■□□□■■■■
M. africanum 981538	BENIN	orphan	orphan	1	+	-	+	Human	■■■■■■■□□□□□■□□□□□□ □□■■■■■■■■■■■■■■■□□□□■■■
M. africanum ATCC25420	UNKNOWN	187	AFRI_1	1	+	-	+	nd	■■■■■■□□□■■■■■■■■■■ ■■■■■■■■■■■■■□■■■■■□■■■■
M. africanum 1255/93	SIERRA LEONE	187	AFRI_1	1	+	-	+	nd	■■■■■■□□□■■■■■■■■■■ ■■■■■■■■■■■■■□■■■■■□■■■■
M. africanum 1567/93	SIERRA LEONE	187	AFRI_1	1	+	-	+	nd	■■■■■■□□□■■■■■■■■■■ ■■■■■■■■■■■■■□■■■■■□■■■■
M. africanum 1457/93	SIERRA LEONE	orphan	orphan	1	+	-	+	nd	■■■■■■□□□■■■■■□□□□□ □□□□□■■■■■■■■□■■■■■□■■■■
M. africanum 1565/93	SIERRA LEONE	187	AFR_1	1	+	-	+	nd	■■■■■■□□□■■■■■■■■■■ ■■■■■■■■■■■■■□■■■■■□■■■■
M. africanum AF0236	UNKNOWN	nd	nd	1	+	-	+	nd	nd
M. africanum AF0271	UNKNOWN	nd	nd	1	+	-	+	nd	nd
M. africanum NR 13604	LIBERIA	orphan	orphan	1	+	-	+	Human	■■■■■■□□□■■■■■■■■■■ ■■■■■■■■■■■□■■■■■■■□■■■■
M. africanum 15133 MC1	UNITED STATES	326	AFRI_1	1	+	-	-	Human	■■■■■■□□□■■■■■■■■■■ ■■□□□■■■■■■■■■■■■■■□■■■■
M. microti 140050001	UNKNOWN	641	MICROTI	1	+	-	+	Llama	□□□■■■■□□□□□□□□□□□□ □□□■■□□□□□□□□□□□□■■□□□□□
M. microti 140050002	UNKNOWN	539	MICROTI	1	+	-	+	Vole	□□□□□□□□□□□□□□□□□□□ □□□□□□□□□□□□□□□□□■■□□□□□
M. microti 140050003	UNKNOWN	641	MICROTI	1	+	-	+	Llama	□□□■■■■□□□□□□□□□□□□ □□□■■□□□□□□□□□□□□■■□□□□□

M. microti 140050004	UNKNOWN	539	MICROTI	1	+	-	+	Cat	□□□□□□□□□□□□□□□□□□□ □□□□□□□□□□□□□□□□□■■□□□□□
M. microti 140050005	UNKNOWN	539	MICROTI	1	+	-	+	Pig	□□□□□□□□□□□□□□□□□□□ □□□□□□□□□□□□□□□□□■■□□□□□
M. microti 140050006	UNKNOWN	539	MICROTI	1	+	-	+	Vole	□□□□□□□□□□□□□□□□□□□ □□□□□□□□□□□□□□□□□■■□□□□□
M. microti 140050007	UNKNOWN	539	MICROTI	1	+	-	+	Vole	□□□□□□□□□□□□□□□□□□□ □□□□□□□□□□□□□□□□□■■□□□□□
M. microti 140050008	UNKNOWN	539	MICROTI	1	+	-	+	Vole	□□□□□□□□□□□□□□□□□□□ □□□□□□□□□□□□□□□□□■■□□□□□
M. microti 140050010	UNKNOWN	641	MICROTI	1	+	-	+	Cat	□□□■■■■□□□□□□□□□□□□ □□□■■□□□□□□□□□□□□■■□□□□□
M. microti ATCC19422	UNKNOWN	2878	MICROTI	1	+	-	+	Vole	□□□■■■■□□□□□□□□□□□■ □□□■■□□□□□□□□□□□□■■□□□□□
M. microti ATCC35782	UNKNOWN	2878	MICROTI	1	+	-	+	Vole	□□□■■■■□□□□□□□□□□□■ □□□■■□□□□□□□□□□□□■■□□□□□
M. microti 9402272	NETHERLANDS	539	MICROTI	1	+	-	+	Human	□□□□□□□□□□□□□□□□□□□ □□□□□□□□□□□□□□□□□■■□□□□□
M. microti 9702257	NETHERLANDS	539	MICROTI	1	+	-	+	Human	□□□□□□□□□□□□□□□□□□□ □□□□□□□□□□□□□□□□□■■□□□□□
M. microti 15496	UNKNOWN	539	MICROTI	1	+	-	+	Vole	□□□□□□□□□□□□□□□□□□□ □□□□□□□□□□□□□□□□□■■□□□□□
M. microti 15498	UNITED KINGDOM	539	MICROTI	1	+	-	+	Vole	□□□□□□□□□□□□□□□□□□□ □□□□□□□□□□□□□□□□□■■□□□□□
M. microti 16240	UNKNOWN	2878	MICROTI	1	+	-	+	Vole	□□□■■■■□□□□□□□□□□□■ □□□■■□□□□□□□□□□□□■■□□□□□
M. microti 15912	BELGIUM	641	MICROTI	1	+	-	+	Llama	□□□■■■■□□□□□□□□□□□□ □□□■■□□□□□□□□□□□□■■□□□□□
M. pinnipedii 41	FRANCE. METROPOLITAN	593	PIN	1	+	-	+	Seal	□□□■■■■□□□□□□□□□□□□ □□□■■■■■■■■■■■■■■■■□□□□□
M. pinnipedii FCC69	AUSTRALIA	2879	PIN	1	+	-	+	Seal	□□□■■■■□□□□□□□□□□□■ □□□■■■■■■■■■■■■■■■■□□□□□
M. pinnipedii FCC70	AUSTRALIA	2879	PIN	1	+	-	+	Seal	□□□■■■■□□□□□□□□□□□■ □□□■■■■■■■■■■■■■■■■□□□□□
M. pinnipedii FCC72	AUSTRALIA	2879	PIN	1	+	-	+	Seal	□□□■■■■□□□□□□□□□□□■ □□□■■■■■■■■■■■■■■■■□□□□□
M.caprae 140020064	FRANCE. METROPOLITAN	orphan	orphan	1	+	-	+	Human	■■□■■■■■□□□□□□□□□□■ ■■■□■■■■■■■■■■■■■■■□□□□□
M.caprae CIP 105776	SPAIN	nd	nd	1	+	-	+	Goat	nd
Dassie Bacillus 68/7171	AUSTRALIA	2880	Unknown	1	+	-	+	Dassie	■■■■■■□■□■■■■■□□■■□ □□□□□□□□□□□□□□□□■■■□■■■■
Dassie Bacillus FCC49	AUSTRALIA	2880	Unknown	1	+	-	+	Dassie	■■■■■■□■□■■■■■□□■■□ □□□□□□□□□□□□□□□□■■■□■■■■
Dassie Bacillus140050009	UNKNOWN	2881	Unknown	1	+	-	+	Hyrax	■■■■■■□■□■■■■■□□■■■ □□□□□□□□□□□□□□□□■■■□■■■■
M. canettii 140050009	FRANCE. METROPOLITAN	592	CANETTII	1	-	-	+	Human	□□□□□□□□□□□□□□□□□□□ □□□□□□□□□□■□□□□□■□□□□□□□
M. canettii 19970130	FRANCE. METROPOLITAN	592	CANETTII	1	-	-	+	Human	□□□□□□□□□□□□□□□□□□□ □□□□□□□□□□■□□□□□■□□□□□□□
M. canettii 19990121	SWITZERLAND	592	CANETTII	1	-	-	+	Human	□□□□□□□□□□□□□□□□□□□ □□□□□□□□□□■□□□□□■□□□□□□□
M. canettii 19981514	DJIBOUTI	592	CANETTII	1	-	-	+	Human	□□□□□□□□□□□□□□□□□□□ □□□□□□□□□□■□□□□□■□□□□□□□
M. canettii 19990589	DJIBOUTI	592	CANETTII	1	-	-	+	Human	□□□□□□□□□□□□□□□□□□□ □□□□□□□□□□■□□□□□■□□□□□□□
M. canettii 19991708	DJIBOUTI	592	CANETTII	1	-	-	+	Human	□□□□□□□□□□□□□□□□□□□ □□□□□□□□□□■□□□□□■□□□□□□□

STB140070001	DJIBOUTI	nd	ST group B[§]	1	Absent	-	+	Human	□□□□ □□□□□□□□□□□□□□□□□□□□□□□ □□□□□□□□□□□□□□□□
STB140070007	DJIBOUTI	nd	ST group I[§]	1	-	-	+	Human	□□□□□□□□□□□□□□□□□□□ □□□□□□□□□□■□□□□□■□□□□□□□
