# Supplementary material for: Insights into the evolutionary history of tubercle bacilli as disclosed by genetic rearrangements within a PE_PGRS duplicated gene pair
Source: BMC Evol Biol. 2006 Dec 12;6:107. doi: 10.1186/1471-2148-6-107 (PMC1762029; doi:10.1186/1471-2148-6-107)
Supplement: Additional file 5 — Summary statistics of the genetic diversity within PE_PGRS17 and PE_PGRS18 sequenced regions. The statistics were generated using the software programs Arlequin v.2.0 [57] and DNASP [58]. M. tb: M. tuberculosis; M. afr: M. africanum; M. mic: M. microti; M. bov: M. bovis. The subspecies include M. caprae, M. pinnipedii, and dassie bacillus. M. can: M. canettii; STB: smooth tubercle bacillus. s: synonymous; a: nonsynonymous; NA: not applicable. [file 1471-2148-6-107-S5.pdf]

	M. tb (n=41)		*M. afr-M. mic-M. bov and subspecies* (n=56)		*M. can* (n=2) and STB (n=2)	
	**PE_PGRS17**	**PE_PGRS18**	**PE_PGRS17**	**PE_PGRS18**	**PE_PGRS17**	**PE_PGRS18**
No. of gene copies	41	41	56	56	3	4
No. haplotypes	3	13	7	5	1	3
No. of polymorphic sites	2	18	8	9	21	14
Number of observed transitions	2	9	5	5	10	7
Number of observed transversions	0	9	3	3	10	6
Pi(s)	0,00429	0,02656	0,003	0,008	0	0.02035
Pi(a)	0,00171	0,00612	0,003	0,003	0	0.00528
Mean Ka (Range)	0.0013 (0 to 0.0026)	0,006 (0 to 0,012)	0,00321 (0 to 0,008)	0,003 (0 to 0,006)	0	0.0053 (0.004 to 0.008)
Mean Ks (Range)	0.0065 (0.0065)	0,027 (0,006 to 0,06)	0,00306 (0 to 0,005)	0,008 (0 to 0,015)	0	0.02063 (0.0206 to 0.0207)
Mean Ka/Ks (Range)	0.2 (0 to 0.4)	0,273 (0 to 1,569)	1,019 (0 to 1,588)	0,293 (0 to 0,386)	NA	0.258 (0.193 to 0.388)
