# Supplementary material for: Insights into the evolutionary history of tubercle bacilli as disclosed by genetic rearrangements within a PE_PGRS duplicated gene pair
Source: BMC Evol Biol. 2006 Dec 12;6:107. doi: 10.1186/1471-2148-6-107 (PMC1762029; doi:10.1186/1471-2148-6-107)
Supplement: Additional file 6 — Statistical analysis of ANOVA and a Tukey's test used to test for significant difference of the GC content and substitution rates within the three codon positions for both PE_PGRS17 and PE_PGRS18 genes. For this purpose, each unique sequence for each gene was assigned a different number (ST1 to ST10 and ST1 to ST19 for PE_PGRS17 and PE_PGRS 18, respectively). [file 1471-2148-6-107-S6.pdf]

Analysis of Rv0978c	GC content %			Substitutions/Site		
	Pos1	Pos2	**Pos3**	Pos1	Pos2	**Pos3**
ST1 (*M. tuberculosis*)	71.9	65.9	**85.7**	1	0	**0**
ST2 (*M. tuberculosis*)	72.5	65.9	**86.3**	0	0	**1**
ST3 (*M. caprae*)	72.5	65.4	**86.8**	2	1	**2**
ST4 (*M. bovis*)	72.5	65.4	**86.2**	1	1	**2**
ST5 (*M. bovis*)	72.5	65.4	**86.2**	0	1	**2**
ST6 (*M. bovis* BCG)	72.5	65.4	**86.2**	0	1	**2**
ST7 (*M. pinnipedii*)	72.5	65.4	**86.8**	1	1	**3**
ST8 (*M. africanum*)	72	65.9	**87.3**	2	0	**4**
ST9 (*M. canettii*)	73.9	66.1	**87.3**	5	2	**12**
ST10 (*M. microti*)	68.1	60.4	**85.4**	1	0	**1**

Analysis of the GC content position by position

One-way analysis of variance

P value	*P*<0.0001
P value summary	Yes

Tukey's Multiple Comparison Test

	Mean Diff.	*P* value	95% CI of diff
pos1 vs pos2	6,970	*P* < 0.001	1.756 to 4.334
pos1 vs pos3	-14,33	*P* < 0.001	-8.894 to -6.316
pos2 vs pos3	-21,30	*P* < 0.001	-11.94 to -9.361

Analysis of the distribution of substitutions

One-way analysis of variance

P value	0.0820
P value summary	NS

Tukey's Multiple Comparison Test

	Mean Diff.	*P* value	95% CI of diff
pos1 vs pos2	0.6000	*P* > 0.05	-1.808 to 3.008
pos1 vs pos3	-1.600	*P* > 0.05	-4.008 to 0.8075
pos2 vs pos3	-2.200	*P* > 0.05	-4.608 to 0.2075

**Analysis of Rv0980c**	**GC content %**			**Substitutions/Site**		
	Pos1	Pos2	**Pos3**	Pos1	Pos2	**Pos3**
ST1 (*M. tuberculosis*)	75	66.9	**79.4**	0	0	**5**
ST2 (*M. tuberculosis*)	75	66.3	**79.9**	0	1	**4**
ST3 (*M. tuberculosis*)	75	66.9	**84.7**	0	0	**1**
ST4 (*M. tuberculosis*)	75	66.9	**80.4**	0	0	**1**
ST5 (*M. tuberculosis*)	75	66.9	**79.4**	0	0	**1**
ST6 (*M. tuberculosis*)	75.6	66.9	**78.3**	2	0	**4**
ST7 (*M. tuberculosis*)	75	66.9	**78.8**	0	0	**3**
ST8 (*M. tuberculosis*)	77.4	66.9	**79.3**	1	0	**9**
ST9 (*M. tuberculosis*)	74.5	66.9	**79.8**	2	1	**8**
ST10 (*M. tuberculosis*)	73.9	66.9	**80.4**	2	0	**6**
ST11 (*M. tuberculosis*)	74.4	66.9	**80.9**	1	0	**5**
ST12 (*M. tuberculosis*)	75	66.9	**78.8**	1	1	**6**
ST13 (*M. africanum)*	75.6	66.9	**78.8**	2	0	**3**
ST14 (*M. bovis* BCG)	75	66.9	**79.4**	0	0	**1**
ST15 (*M. microti*)	75	66.9	**78.8**	0	0	**0**
ST16 (*M. pinnipedii*)	73.7	75.6	**78.8**	2	0	**3**
ST17 (*M. caprae*)	75	66.9	**79.4**	0	0	**1**
ST18 (*M. canetti*)	70.3	71.2	**79.9**	5	0	**1**
ST19 (*M. bovis*)	75	66.9	**79.4**	0	0	**1**

Analysis of the GC content position by position

One-way analysis of variance

P value	*P*<0.0001
P value summary	Yes

Tukey's Multiple Comparison Test

	Mean Diff.	*P* value	95% CI of diff
pos1 vs pos2	4	*P* < 0.001	2.421 to 4.398
pos1 vs pos3	-4.632	*P* < 0.001	-3.304 to -1.327
pos2 vs pos3	-11.45	*P* < 0.001	-6.714 to -4.736

Analysis of the distribution of substitutions

One-way analysis of variance

P value	*P*<0.0001
P value summary	Yes

Tukey's Multiple Comparison Test

	Mean Diff.	*P* value	95% CI of diff
pos1 vs pos2	0.6250	*P* > 0.05	-0.7751 to 2.025
pos1 vs pos3	-2.938	*P* < 0.001	-4.338 to -1.537
pos2 vs pos3	-3.563	*P* < 0.001	-4.963 to -2.162
