# Supplementary figures and images for: Insights into the evolutionary history of tubercle bacilli as disclosed by genetic rearrangements within a PE_PGRS duplicated gene pair
Source: BMC Evol Biol. 2006 Dec 12;6:107. doi: 10.1186/1471-2148-6-107 (PMC1762029; doi:10.1186/1471-2148-6-107)

A

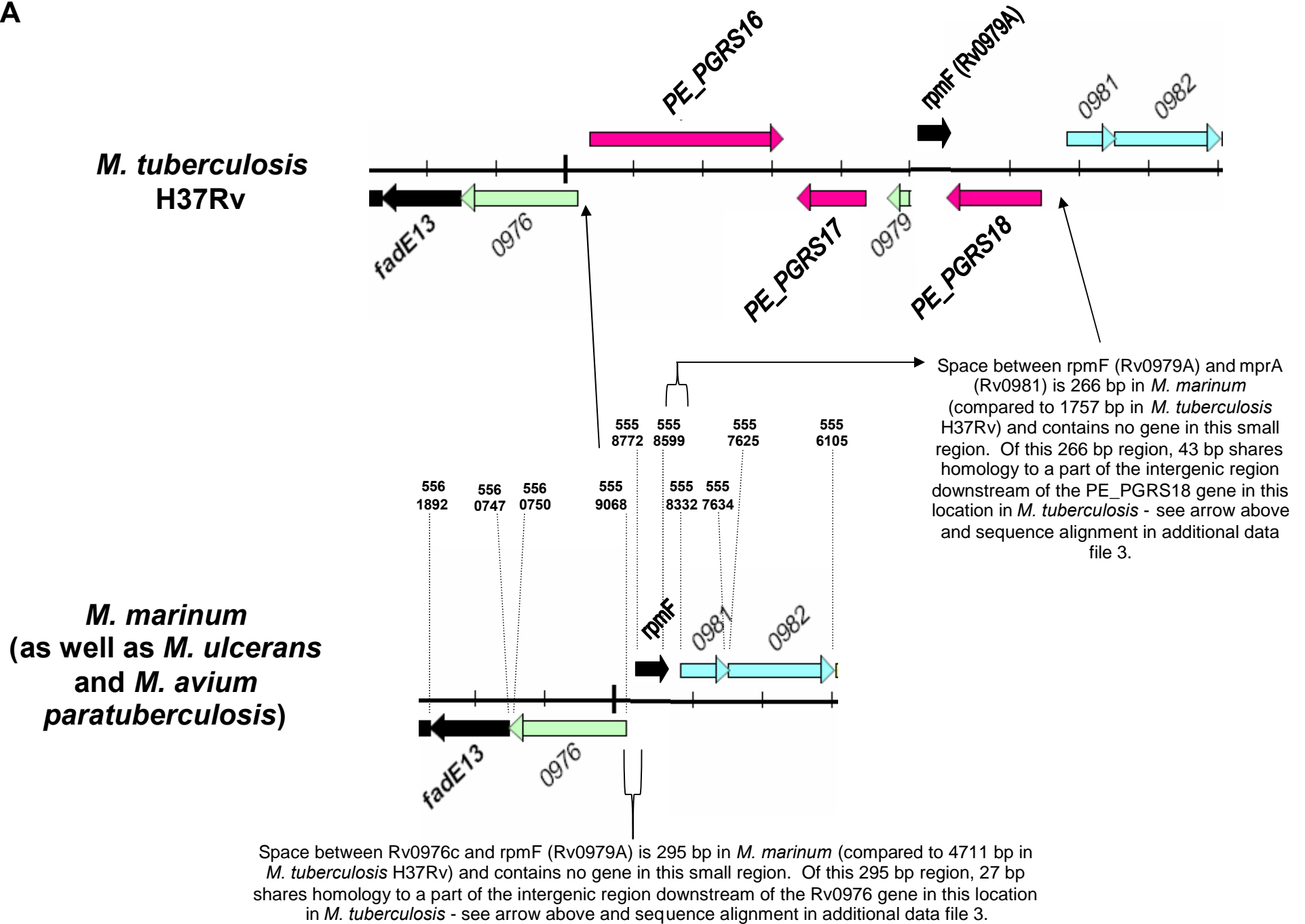

**B***M. tuberculosis* strain H37Rv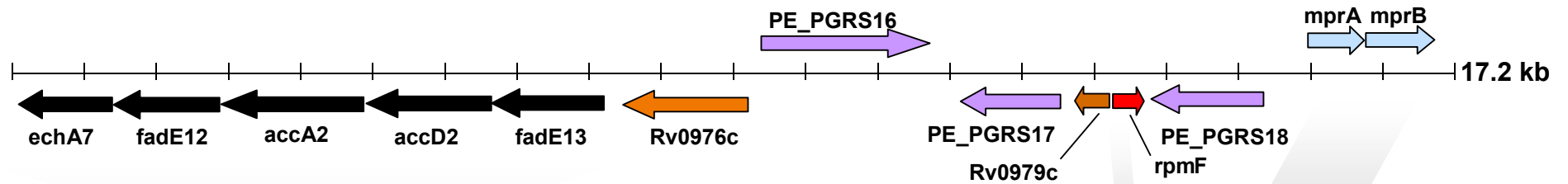*M. leprae* strain TN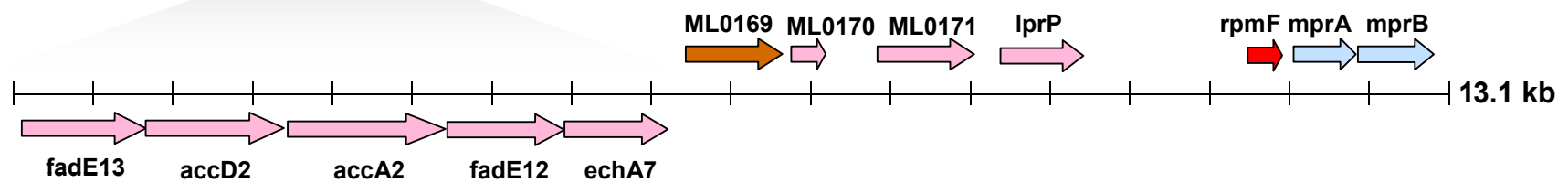

Supplement: Additional file 2 — Schematic representation of the genes (shown by arrows) and gene order of the genomic region containing PE_GRS17 and PE_PGRS18 genes. (A) Comaprison of M. tuberculosis with M. marinum, M. ulcerans, and M. avium subspecies paratuberculosis. (B) Comparison between M. tuberculosis and M. leprae. Shaded areas indicate homologous regions. Note the inversion of the region encompassing echA7 to fadE13 between the two latter species. [file 1471-2148-6-107-S2.pdf]
